# Greater pollen-mediated gene flow among than within populations of a bumblebee-pollinated forest herb despite habitat fragmentation

**DOI:** 10.1186/s40462-026-00664-8

**Published:** 2026-05-30

**Authors:** Jannis Till Feigs, Siyu Huang, Katja Kramp, Stephanie I. J. Holzhauer, Tobias Naaf

**Affiliations:** 1 Leibniz Centre for Agricultural Landscape Research (ZALF) e. V., Eberswalder Strasse 84, D-15374 Müncheberg, Germany; 2 Thünen Institute of Biodiversity, Johann Heinrich von Thünen Institute, Forestry and Fisheries, Federal Research Institute for Rural Areas, 38116 Braunschweig, Germany

**Keywords:** Agricultural landscapes, *Bombus pascuorum*, *Bombus pratorum*, Floral resources, Linear landscape elements, Paternity analysis, *Polygonatum multiflorum*

## Abstract

**Background:**

Pollen flow within and among spatially isolated forest herb populations in agricultural landscapes depends on the movement activity of their pollinators. For central-place and traplining foragers, such as bumblebees, this movement activity is expected to reflect how they exploit floral resources across the landscape. However, it remains unclear how the bumblebees’ foraging behavior actually shapes the mating patterns of forest herbs within and among forest patches.

**Methods:**

Here, we conducted a landscape genetic analysis of two generations of the forest herb *P. multiflorum* using microsatellites to investigate (A) the proportions of pollen-mediated gene flow within and among forest patches, (B) how the spatial position and size of shoot clusters influence the pollen-mediated gene flow within forest patches, and (C) how landscape composition affects pollen immigration rates into forest patches.

**Results:**

We found that pollen immigration rates per pollen receptor plant were high, with only a few shoot clusters within a forest patch serving as pollen donors. Larger shoot clusters had higher probabilities of donating pollen. Both pollen immigration rates per pollen receptor and the genetic diversity of the offspring were significantly affected by maize cover and linear landscape elements in the surrounding landscape. We found that in landscapes dominated by land-use types with low floral-resource availability, higher pollen immigration rates into wild plant populations do not necessarily lead to higher genetic diversity.

**Conclusions:**

Our results suggest that trapline foraging promotes greater pollen flow among forest patches than within them, and that shoot clusters within a forest patch contribute unequally to sexual reproduction. In the studied agricultural landscape, semi-natural habitat patches with high floral-resource availability may either compete for or facilitate pollinator activity and the resulting gene flow among forest patches, while smaller patches act rather as stepping stone habitats, larger ones may retain pollinators and distract them from less attractive forest herb populations. Similarly, linear landscape elements oriented towards pollen receptors increased both the amount and allelic richness of incoming pollen, whereas those oriented orthogonally had the opposite effect. The study also emphasized that self-incompatible bumblebee-pollinated plant species in resource-poor landscapes may form metapopulation with regular pollen exchange which does not automatically lead to high levels of introduced new alleles.

**Supplementary information:**

The online version contains supplementary material available at 10.1186/s40462-026-00664-8.

## Background

Globally, animal-pollinated forest plants are among the organism groups most affected by habitat fragmentation and loss [1]. One major consequence of fragmentation is altered abundance and foraging behavior of these animals [2–8], which play an important role as genetic linkers by transferring pollen between spatially isolated populations [9, 10]. In Europe, a large proportion of forest herb populations occurs in small, spatially isolated forest patches embedded within agricultural landscapes [11, 12]. The extent to which fragmentation affects these populations depends partly on specific plant traits [13, 14]. For example, floral morphology may restrict flower visitors to particular pollinator groups, and different pollinator types are expected to respond differently to habitat fragmentation [8, 15], with large-bodied species being more likely to overcome spatial isolation. In addition, plant traits related to the mating system [16, 17] or clonality [18, 19] influence how pollinator foraging patterns translate into mating patterns, that is, which pairs of individuals produce offspring [20, 21]. However, it remains unclear how mating patterns of spatially isolated forest herb populations are structured across agricultural landscapes and how this shapes their future genetic structure.

In bumblebee-pollinated forest herb species, workers’ foraging likely shapes mating patterns within and among isolated herb populations. Bumblebees are among the most important pollinators of wild plant species [22, 23], including forest herb populations embedded within agricultural landscapes [24, 25]. The foraging patterns of bumblebee workers comprises local, intermediate, and distant spatial scales [23, 26], corresponding to mating among flowers of the same plant individual, among individuals within a habitat patch, and among different habitat patches [23]. Much of the foraging activity is expected to occur at local scales, as workers tend to forage efficiently and minimize flight distances, visiting multiple flowers within a single inflorescence or nearby neighbors [23, 27, 28]. However, for various bumblebee species, foraging ranges of several hundred meters to several kilometers have been shown [23, 29–31]. The workers may visit multiple habitat patches during a single foraging bout [32, 33], potentially carrying pollen among distinct plant populations. Analysis of contemporary pollen-mediated gene flow within a single year is therefore particularly informative for understanding pollinator-driven mating patterns. For example, in *Heliconia tortuosa*, pollen analysis revealed that hummingbird pollinators using traplining, i.e., repeatable, multi-destination foraging routes, strongly shape contemporary pollen-mediated gene flow, resulting in high connectivity among isolated forest patches [34, 35]. Although traplining in bumblebees has been demonstrated in laboratory and field studies [36–40], it remains unclear whether similar mating patterns are established by the foraging activity of bumblebee workers visiting forest herbs within agricultural landscapes. Clarifying this is essential for assessing forest herb populations’ functional connectivity, defined as the effective dispersal of pollen or propagules among habitat patches within a specific landscape context [41].

Within a forest patch, the foraging behavior of bumblebees, and thus plant mating patterns, will be influenced by differences in attributes among plant individuals and populations, such as population size and life-history traits. Habitat fragmentation typically results in smaller populations [3, 42, 43]. Such populations face an increased risk of sexual extinction, i.e., a situation in which the number of remaining alleles and genotypes is so low that sexual reproduction becomes nearly impossible [19, 44]. Besides biparental inbreeding (which is restricted in self-incompatible species) [44, 45], the dominance of a few pollen donors can substantially reduce offspring genetic diversity [44, 46, 47]. Such dominance may be linked to traits of the pollen donors. The majority of temperate forest herbs reproduce both sexually and vegetatively, with approximately one-third being strictly self-incompatible [48]. The clonal growth of a substantial proportion of these species forms patches or shoot clusters within a small area [17]. Combined with their perennial life history, this leads to differences in the number of flowers among shoot clusters of varying size [49]. Larger clones are therefore expected to benefit from their higher number of flowers [20, 50–52] and may dominate reproduction within a habitat patch [53]. The larger numbers of flowers of these clones might increase their chances of mating not only through pure quantity, but also through a greater likelihood of attracting more pollinators [8, 20, 49, 54]. In addition, spatial position within a forest fragment immediate flowering neighborhood may also influence mating success [47]. Most insect pollinators, including bumblebees, preferentially visit nearby plants to maximize foraging efficiency [14, 47, 55, 56]. As many bumblebee species nest along forest edges or linear landscape elements [57], edge-located shoot clusters may receive more visits ([58, 59]; but see [60]). If a few large or edge-positioned clusters dominate pollen donation, the effective population size may decline, thereby constraining the evolutionary potential of small populations. Fluorescent dye studies, sometimes combined with molecular paternity analysis, have provided insights into mating patterns in forest herbs pollinated, among others, by bumblebees [28, 61]. However, comprehensive paternity analyses covering multiple entire populations of species specializing in bumblebee pollination within agricultural landscape are still lacking. Such analyses are essential for quantifying effective population size and identifying potential causes of asymmetric contributions among different pollen donors.

Landscape composition at a larger spatial scale influences bumblebee foraging patterns and may thereby shape pollen-mediated gene flow among spatially isolated forest herb populations. The strength of this gene flow can be quantified using pollen immigration rates, defined as the proportion of total measured pollen-mediated gene flow that originates from donors outside the receptors’ own population [62]. Reported immigration rates vary widely among species and populations (low with values below 0.2 in [43, 63; 64; 65]: intermediate with values between 0.2 and 0.4 in [66]: and high with values up to 0.77 in [56]). Not only do pollen immigration rates vary among populations, but they can also vary considerably among individual receptors within a population [67]. These pollen immigration rates are not solely a function of geographic distance; they also reflect the landscape composition and structure within specific distance classes [14]. Because many bumblebee species are habitat generalists in agricultural landscapes [30, 68], their effectiveness as genetic linkers likely depends strongly on the landscape composition. In particular, floral resource distribution and landmarks that guide navigation influence the movement patterns [69]. Pollinators are more likely to move among habitat patches separated by land-use types with limited floral resources [70], suggesting higher pollen immigration when forest patches are surrounded by crops with limited floral resources, such as maize [71]. In contrast, land-use types that provide abundant floral resources in spring, such as semi-natural grassland or rapeseed fields, may distract pollinators from moving among forest herb populations [72, 73]. An important further aspect is that in landscapes with fewer floral resources, bumblebees will probably show higher flower constancy, as their flower constancy strongly depends on the remaining available resources [74, 75]. Linear landscape elements, such as woody elements, roads, or water courses, may further guide or constrain bumblebee movement, functioning as corridors depending on their configuration [76–79]. Despite these known facts, the link between landscape composition and pollinator-mediated gene flow in temperate forest herb populations remains poorly understood. Analyses based on established adult individuals largely reflect past landscape compositions [80–83] and cumulative gene flow over multiple years [84]. In contrast, assessing contemporary pollen-mediated gene flow provides direct insights into current landscape effects.

In this study, we aim to characterize the contemporary mating patterns of an outcrossing forest herb mediated by bumblebee foraging within and among forest patches by using paternity analysis, with particular emphasis on within-patch mating patterns and the influence of landscape composition and structure on among-patch mating. This work will contribute to our general understanding of the functional connectivity of bumblebee pollinated wild plant populations within agricultural landscapes.

Specifically, we address the following four hypotheses:

### H1:

Most of the pollen-mediated gene flow occurs within forest patches due to spatial isolation, but a detectable portion of pollen-mediated gene flow occurs among forest patches as a result of bumblebee foraging in multiple forest patches.

### H2:

The chance of being an active pollen donor within the own forest patch increases with (A) larger shoot cluster size and (B) shorter distance to the forest edge.

### H3:

The pollen immigration rate and genetic diversity largely depend on the distribution of floral resources in the surrounding landscape. Specifically, we expect them to be high with a higher proportion of land-use types poor in floral resources, such as maize fields, and low with a higher proportion of land-use types rich in floral resources, such as semi-natural grassland and oilseed rape fields.

### H4:

The pollen immigration rate and genetic diversity also depend on the likelihood that pollinators are guided towards the pollen receptors. We anticipate that this guidance will be stronger with more linear landscape elements that are directed towards the pollen receptor.

## Materials & methods

### Study species

The species *Polygonatum multiflorum* (L.) ALL. is a slow-colonizing forest specialist [85, 86] that reproduces both sexually and vegetatively [87]. It grows patchily in shoot clusters that increase in number of shoots over time (Figure S1A, B). We defined a shoot cluster as a group of at least partly flowering shoots no more than 30 cm apart. The species blooms in spring, is strictly outcrossing, and depends on insect pollination [88]. The flowers are mainly hermaphroditic [87]. The species produces up to 15 inflorescences per shoot, each bearing two to five flowers. Flowering lasts from 10 days to several weeks. During this time, the flowers open sequentially from the bottom to the top, with usually three open flowers at a time [89]. In our landscape window, the main flowering period occurred during the first two weeks of May, with the first flowers opening around one week earlier and the last ones about one week later. The long corolla makes long-tongued bumblebees the most important pollinators of this species [10, 87, 90]. For the closely related forest herb species *Polygonatum odoratum*, it has been described that bumblebee pollinators systematically visit multiple flowers along an inflorescence [27] and during field work we observed a similar behaviour. The bumblebee species *Bombus pascuorum* (SCOPOLI, 1763) and *Bombus pratorum* (LINNAEUS, 1761) were identified as the most common flower visitors of *P. multiflorum* populations in small forest patches within northern German agricultural landscapes [90], Figures S1C, D; Table S1). These species are nest-builders with central place-foraging behaviour. Forest and forest edges serve as both potential nesting and foraging habitats [57, 91, 92]. For workers of *B. pascuorum* and *B. pratorum*, foraging ranges of several hundred meters are typical [23, 30]. While *B. pascuorum* is considered a “doorstep forager”, with workers foraging as close to the nest as possible [93], foraging distances of a few kilometres have also been reported [30]. Of the five bumblebee species that we observed as flower visitors of *P. multiflorum *Supplement Table S1, workers of *B. pascuorum* have been shown to visit both plant shoots occurring at high and low densities, whereas the other species prefer plant patches with high densities [94]. The identity of seed vectors of *P. multiflorum* is unclear, but its few, heavy fruits indicate a low seed-dispersal potential [86]. Long-distance dispersal of its toxic fleshy berries by birds or mid-sized carnivores is considered rare [95–97], while short-distance dispersal by rodents might occur more frequently [98].

### Sampling and germination

**Fig. 1:**
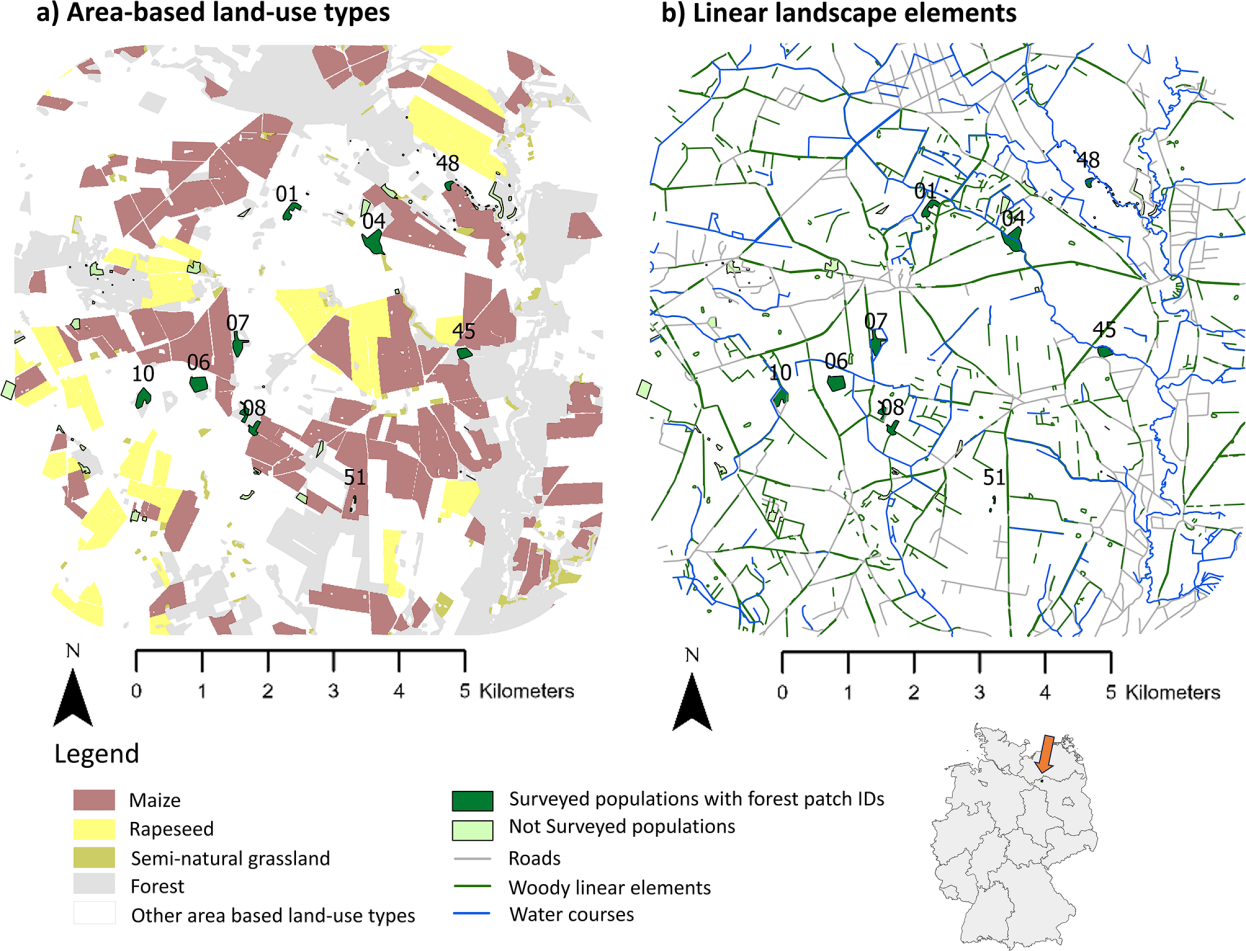
Maps of the studied 5 km × 5 km landscape window plus a 2 km buffer displaying the distribution of *P. multiflorum* populations (a and b), area-based land-use types including the crop cover in 2018 (**a**) and linear landscape elements (**b**). The position of the study site in Germany is shown in the lower right corner. Most of the other area-based land-use types were cereal fields, intensive grassland, and settlements

We conducted this study within a 5 km × 5 km landscape window in the *Prignitz* region, eastern Germany (Fig. 1). This region is predominantly characterized by agricultural land use. In May 2018, we sampled leaf material from all findable shoot clusters with flowering shoots of *P. multiflorum* across nine forest patches (Figs. 1). The number of sampled shoot clusters per forest patch ranged from 24 to 689 (Table S2.1). Most shoot clusters contained less than 10 shoots (85% of total shoot clusters; Table S2.1). However, in all forest patches except one (F45), there were also large shoot clusters comprising 20 to 269 flowering shoots (Table S2.1). We took one leaf sample per shoot cluster under the assumption that the majority of the shoots within a cluster would be clones ([99; 100]; see next section).

In September 2018, we sampled berries from 6 to 14 flowering shoots per forest patch (Table S2.1, Figure S2, Supplement 3), which had been selected as pollen receptor plants based on two criteria: an even spatial distribution within the population area and a sufficient number of berries. From each selected shoot, we collected both berries and leaf material, with up to 1–46 berries per pollen receptor (mean = 17). All berries of a pollen receptor were collected from a single shoot. In one forest patch (F51), the number of pollen receptors with enough fruits was too low in 2018. Therefore, we supplemented the dataset by collecting additional berries from this forest patch in 2019.

**Fig. 2:**
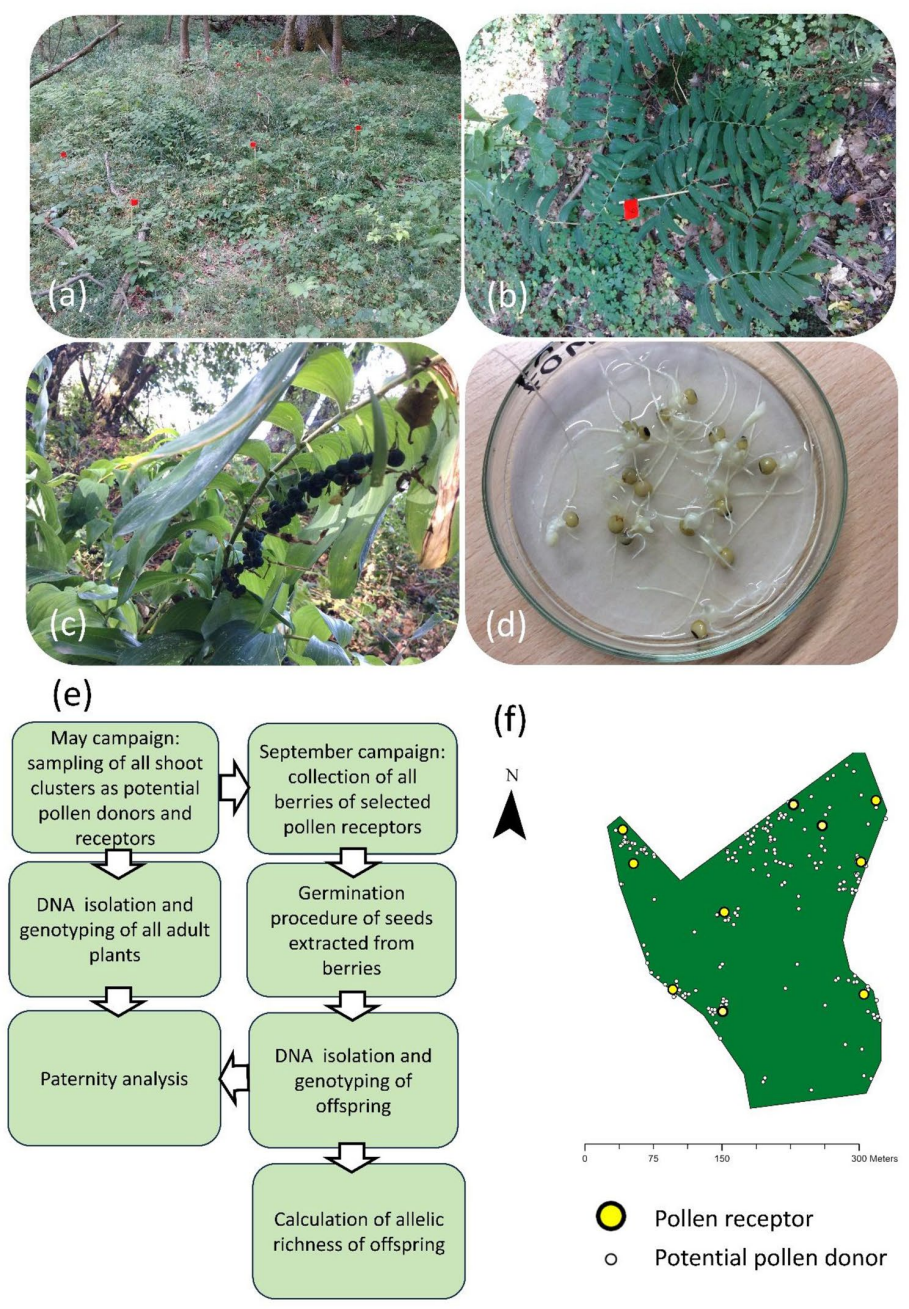
Overview of the data generation procedure. (**a**) Photograph showing a typical situation in a forest patch in the study region with multiple *P. multiflorum* shoot clusters. Each shoot cluster is marked with a red flag. (**b**) Detail of a shoot cluster composed of multiple shoots. (**c**) a selected pollen receptor plant with berries in September. (**d**) Rhizomes from extracted seeds after germination, (**e**) schematic of the data generation procedure. (**f**) Example of distribution of the sampled shoot clusters within a forest patch (shown is the forest patch 04 in Fig. 1). Small white dots represent all sampled shoot cluster, and larger yellow dots indicate those additionally used as pollen receptors. Detail maps of the sampling for all nine forest patches are provided in Figure S2

In the laboratory, we extracted the seeds from the collected berries and treated them to induce germination (Supplement 3). They were kept in a climate chamber at 5 °C for six weeks under wet conditions. After this stratification period, the seeds were kept in the dark at approximately 20 °C for seven additional weeks until a small rhizome had formed (Supplement 3, Fig. 2d).

### DNA extraction and genotyping

We extracted DNA from leaves of 2359 shoots of adult plant individuals, of which 91 were pollen receptor individuals (mean per forest patch: 10.1, min: 5 in F45, max: 14 in F51), and from the rhizomes of 881 offspring individuals (mean: 97.8, min: 23 in F45, max: 157 in F51) using the Innupret Plant Kit (Analytik Jena AG, Germany). We genotyped DNA samples employing seven polymorphic microsatellite markers. Three of these had been previously used in studies on *P. multiflorum* [90], while four had been newly developed specifically for this study (Table S4.1). These markers collectively yielded 97 different alleles and exhibited genotyping error rates ranging from 0 to 1.22% based on a repetition of 10% of the samples (Table S4.1). With most combinations of only six loci, nearly all multi-locus genotypes (MLGs) could be distinguished (Figure S4.1).

Using this marker set, we detected 288 repeated MLGs (Table S2.1). We removed samples with duplicate MLG following specific criteria: we prioritized the exclusion of samples that were not assigned as pollen receptors. Offspring samples were removed only in the case of apparent twins (i.e., multiple offspring samples with identical MLGs sampled from the same pollen receptor), but not when offspring individuals shared the same MLG as their parent plants. Consequently, after removing repeated MLGs, the dataset still contained repeated MLGs, particularly in offspring-parent pairs.

In order to validate the assumption that most shoot clusters consist of one or only a few genotypes and to estimate how many potential pollen donors might not be represented, we determined the proportion of monoclonal clusters across 59 shoot clusters of varying size, that were more intensively sampled during the campaign in May 2018. For clusters with up to three shoots, leaves from all shoots were sampled, while for larger clusters a representative subset of shoots was sampled (with a distance < 30 cm among sampled shoots) (Table S4.3 and S4.4). Overall, 44% of the shoot clusters were composed of a single genotype. However, the proportion of monoclonal clusters decreased with increasing cluster size: 62% in clusters with fewer than 5 shoots, 46% in clusters with 5 to 20 shoots, and 28% in clusters with more than 20 shoots (Table S4.4). The 87 additional multilocus genotypes that were identified in the assessment of clonality per shoot cluster were also included as potential pollen donors in the assignment analysis (Table S2.1). These genotypes could contribute as pollen donors, allowing us to evaluate the importance of including multiple shoots per shoot cluster.

In total, 2085 genotypes were included as potential pollen donors (including all pollen receptors; mean per forest patch: 231.7, min: 27 in F51, max: 685 in F48; Table S2.1), 90 genotypes as pollen receptors (mean: 10, min: 5 in F45, max: 14 in F51; Table S2.1), and 862 genotypes as offspring (mean per forest patch: 96, min: 20 in F45, max: 150 in F51; mean per pollen receptor: 9.6; min: 1; max: 41; Table S2.1).

### Paternity analysis

We used the software Colony 2 to assign potential pollen donors to the offspring of known pollen receptors [101, 102]. The paternity analysis settings were configured as follows: Both sexes were set to be “polygamous”, and the “without inbreeding” option was selected, as *P. multiflorum* is self-incompatible [87]. All offspring originated from collected fruits and could thus not be clones of each other. Given that we had already removed any repeated MLG, we configured the analysis as “without clones”. We did not use the “updating allele frequency” option, as we did not expect our number of offspring per pollen donor to be very large and highly variable. Since most flowers of *P. multiflorum* are hermaphroditic [103], we set it as a “monoecious species”. Due to the lack of prior knowledge on sibship size, we set sibship scaling to “no”. To ensure the highest possible resolution and reproducibility of results, we used a “very long” run length with the full-likelihood method. Longer runs enable the simulated annealing algorithm to converge on the highest likelihood, thus increasing the probability of finding the most accurate configuration. As this process is computationally intensive, we ran Colony 2 on a high-performance cluster. The “maternity exc threshold” was set to “2”.

In five cases where offspring genotypes mismatched their specific pollen receptors at one locus, the offspring’s alleles at these loci were set to “0 0”, indicating missing data. Paternity analysis was performed separately for each forest patch, as the algorithm assumes random mating [102].

We observed variations in offspring-pollen donor pairs across different runs. To address this, we conducted multiple runs as recommended in the Colony 2 User Guide [104] and in Wang and Santure [105]. Specifically, we performed five independent runs (Table S5.1). An offspring was considered assigned to a pollen donor, if the pair was identified in at least two out of five runs. This relatively conservative criterion, which could potentially produce a false signal of pollen flow within populations, was chosen to minimize the risk of overestimating the amount of pollen originating from outside the population.

### Measures of pollen-mediated gene flow

We used the output from the offspring-pollen donor assignments to determine the number of assigned offspring per shoot cluster for testing effects on within patch mating patterns (H2) and the proportion of offspring per pollen receptor that could be assigned to pollen donors from the own forest patch, which will be referred to as *PF*_*within*_ in the following text for testing among patch mating patterns (H1, H3, and H4). Accordingly, the proportion of pollen not assigned to donors from the same forest patch, here referred to as 1 - *PF*_*within*_, expresses the pollen immigration rate. However, this measure does not provide information about the number or diversity of pollen sources. To assess this diversity, we also calculated the allelic richness (*A*_*r*_) of offspring per pollen receptor [106], which referred to the rarefied number of alleles in the offspring of a pollen receptor. We rarefied this measure to five offspring individuals per pollen receptor. Eleven pollen receptors with less than five berries were excluded from this calculation and further analysis.

### Landscape analysis

We analysed the landscape composition within 50 m, 250 m, and 1000 m radii to represent short-, median- and long-distance pollen-mediated gene flow surrounding each of the remaining 79 pollen receptor plants. To do this, we created a digital land-use map (Fig. 1) based on recent orthophotos according to Naaf et al. [107]. Additionally, we used crop data collected within the European Integrated Administration and Control System (IACS) [108], which allowed us to differentiate crop types, i.e., oilseed rape and maize. We used the IACS data of 2018 or 2019, depending on the sampling year. We calculated the percentage cover of three area-based land-use types and the length of three linear landscape elements (Table 1, Supplement 6). Rapeseed in the study region Brandenburg was in blossom from April 19 to May 17 in 2018 and from April 16 to May 20 in 2019. Maize emergence occurred on May 17 in 2018 and May 06 in 2019 [109]. Regarding the flowering of semi-natural grasslands, we do not have specific records for our landscape windows. However, an increasing provision of floral resources from early May to July is typical for grassland habitat types [110]. Plant species that provide particularly high amounts of floral resources used by bumblebees in grassland habitats begin flowering in Germany in April (e.g. *Taraxacum officinale*, *Ranunculus acris*), May (*Trifolium repens*), or June to July (*Trifolium pratense*, *Cirsium arvense*, *Heracleum sphondylium*) [111, 112]. Additionally, the three linear landscape elements studied here can also serve as habitats for flowering plants [113].

**Table 1:** Landscape metrics used to quantify the landscape composition in buffers of three different sizes (50 m, 250 m, and 1000 m) around pollen receptor plants

Area-based metrics	Percent cover of …	Buffer radii
Rich in floral resources		
SEMNATGRASS	Semi-natural grassland	1000
RAPESEED	Oilseed rape	1000
Poor in floral resources		
MAIZE	Maize	50, 250, 1000
Linear landscape elements	**Length in meters of …**	**Buffer radii**
L_ROAD	Roads (including dirt roads)	50, 250, 1000
L_WATER	Water courses (including water courses and drainage ditches)	50, 250, 1000
L_WOOD	Woody linear elements (including hedgerows and treelines)	250, 1000
O:P	Orthogonal-to-parallel length ratio (predominant orientation separately for each type)	50, 250, 1000
N_P	Total number of *P. multiflorum* populations	250, 1000

In the 50 m and 250 m buffers, not all land-use types were present (Table 1). To account for the potential guiding effect of linear landscape elements, we also calculated their orientation relative to the spatial position of the specific pollen receptor by calculating the orthogonal and parallel length component of each element. A parallel or orthogonal component runs parallel or orthogonally, respectively, to the line connecting the pollen receptor and the midpoint of the linear landscape element (Figure S6.1; see [107] for details on the method). The predominant spatial orientation of all linear elements of a given type in a buffer zone was summarized as the orthogonal-to-parallel length ratio, which was later used as conditioning variable in the statistical models. Since the presence of other populations within the forager’s flight range is a prerequisite for pollen-mediated gene flow among populations, we also counted the total number of *P. multiflorum* populations within the 250 m and 1000 m buffers.

### Data analysis

The data analysis was conducted with R version 4.2.2. For testing effects on the mating pattern within forest patches (H2) and among them (H3 and H4), we used linear mixed models (LMM) with the lme ()-function from the nlme R-package [114], or generalized linear mixed models (GLMM) with the glmmTMB ()- function from the glmmTMB R-package [115, 116] if the response variable had a very high proportion of zero values. In addition to the continuous response variable distribution (the conditional part of the model), this type of model incorporates a point mass at zero to account for excess zeros in the data [115]. It is important to note that this part of the model estimates the probability of an extra zero; thus, higher coefficient values indicate a higher likelihood of a zero, which is opposite in direction to the effect in the conditional part [115].

Prior to modeling, we applied a Box-Cox transformation to all landscape metrics to improve their symmetry. All explanatory variables were then centered and scaled to have a mean of 0 and standard deviation of 1, allowing for the comparison of regression coefficients.

To test how features of the shoot clusters affected within-patch mating patterns (H2), we modeled the number of assigned offspring of 2021 shoot clusters as a function of shoot cluster size and distance to the forest edge. Since most shoot clusters did not serve as effective pollen donors, we used the glmmTMB ()- function as described above. This means the model consisted of both a conditional (cond) and a zero-inflation (zi) component. For the conditional part of the model, we used a Poisson distribution because the response variable was count data. A higher coefficient value in the conditional part indicates that the number of assigned offspring increases with the predictor, suggesting that this predictor promotes the contribution of the specific pollen donor to gene flow. In contrast, for the zero-inflation component, positive effects indicate a higher probability that the shoot cluster did not serve as a pollen donor. We assumed that both explanatory variables influence pollinators’ foraging behavior and may also interact with each other. To account for this, we incorporated an interaction term in both the conditional and zero-inflation component of the model. Forest patches were included as a random factor in both components of the model.

To test the effects of the landscape composition on the quantity and quality of pollen influx (H3 and H4), we modeled *PF*_*within*_ and *A*_*r*_ for 79 pollen receptors as a function of area-based landscape metrics (H3) and linear landscape elements (H4), separately for each of the three buffer radii. For around 47% of receptor plants, no offspring was assigned to any pollen donor from the same forest patch. To account for this zero-inflation in *PF*_*within*_, we used the glmmTMB framework as described above. Since the *PF*_*within*_ was modeled as proportion data, we applied a binomial distribution to the conditional part of the model. In this part, a positive effect indicates a lower pollen immigration rate. The zero-inflation component of the model represents the probability that a pollen receptor received no pollen from donors within the same forest patch. In contrast to the conditional part, a positive effect in the zero-inflation component indicates higher pollen immigration rates. Because *A*_*r*_ approximated a normal distribution, we used linear mixed models to analyze it, with positive effects indicating pollen from more genetically diverse sources. Forest patches were included as a random effect in all models for testing effects of the landscape composition (H3 and H4), including both the conditional and zero-inflation components of models for *PF*_*within*_. Statistical modelling was conducted in three steps to preselect the most relevant predictors and to avoid collinearity. In the first step, we identified candidate explanatory variables by fitting single-metric models for each landscape metric. Metrics were selected if the corresponding model yielded a *p*-value ≤ 0.15. For the GLMMs with a zero-inflation term, we tested the metrics separately for the conditional and zero-inflation component. For area-based landscape elements, we also considered potential curvilinear or unimodal relationships by including a quadratic term when it resulted in a lower *AICc (*Akaike Information Criterion corrected for small sample size [117]). Quadratic terms were included in the models only if the corresponding linear term were also included. For linear landscape elements, we additionally tested whether interactions with the orthogonal-to-parallel length ratio had a significant effect. In the second step, we checked all candidate metrics for collinearity. Variables with a correlation coefficient greater than 0.7 were not included within the same model to avoid collinearity (Supplement S9). In the third step, we ran a global model for each combination of pollen-mediated gene flow measure (*PF*_*within*_ and *A*_*r*_) and buffer radii, including all previously selected metrics as predictors. They were fitted with maximum likelihood (ML) to allow model selection and multi-model inference using the R package MuMIn [118]. From all possible subsets of predictors, we selected subsets based on a *ΔAICc* threshold of ≤2. Quadratic and interaction terms were included only if the corresponding main effect was also present. Finally, we conditionally averaged all selected candidate models [119, 120]. The LMMs were refitted using Restricted Maximum Likelihood (REML) for this purpose, which was not applicable for the GLMMs.

## Results

### Pollen-mediated gene flow within and among forest patches

In total 174 offspring individuals out of 862 (20%) were assigned to pollen donors from their own forest. The median *PF*_*within*_ per pollen receptor was 0.09 (25^th^ percentile = 0 and 75^th^ percentile = 0.33; Figure S7.1a), indicating a prevalent among-forest patch pollen-mediated gene flow. The median *A*_*r*_ per pollen receptor was 2.9 (25^th^ percentile = 2.7 and 75^th^ percentile = 3.1; Figure S7.1b).

### Mating patterns within forest patches

Only 36 out of 2021 (2.7%) shoot clusters included in testing the effects of features of the shoot clusters on within-forest patch mating patters (H2) were identified as effective pollen donors (Figure S7.2). Two of these were also selected as pollen receptors (only in F08). For some shoot clusters, multiple genotypes were included in the assignment procedure; however, no shoot cluster provided more than one effective pollen donor. The median number of offspring per effective pollen donor was 4 (25^th^ quantile: 3 offspring, 75^th^ quantile: 6.25 offspring; Figure S7.2). The median distance between effective pollen donors and pollen receptors within a forest patch was 29 m (range: 0.4–316 m; Figure S7.3). The median distance of all included shoot clusters from the forest edge was 24 m (range: 0.4–112 m), while the median distance of effective pollen donors from the forest edge was 31 m (range: 4.3–96 m). The median shoot cluster size for all clusters was 2 shoots (range: 1–269 shoots), while for effective pollen donors, it was 5.5 shoots (range 1–138 shoots).

**Fig. 3:**
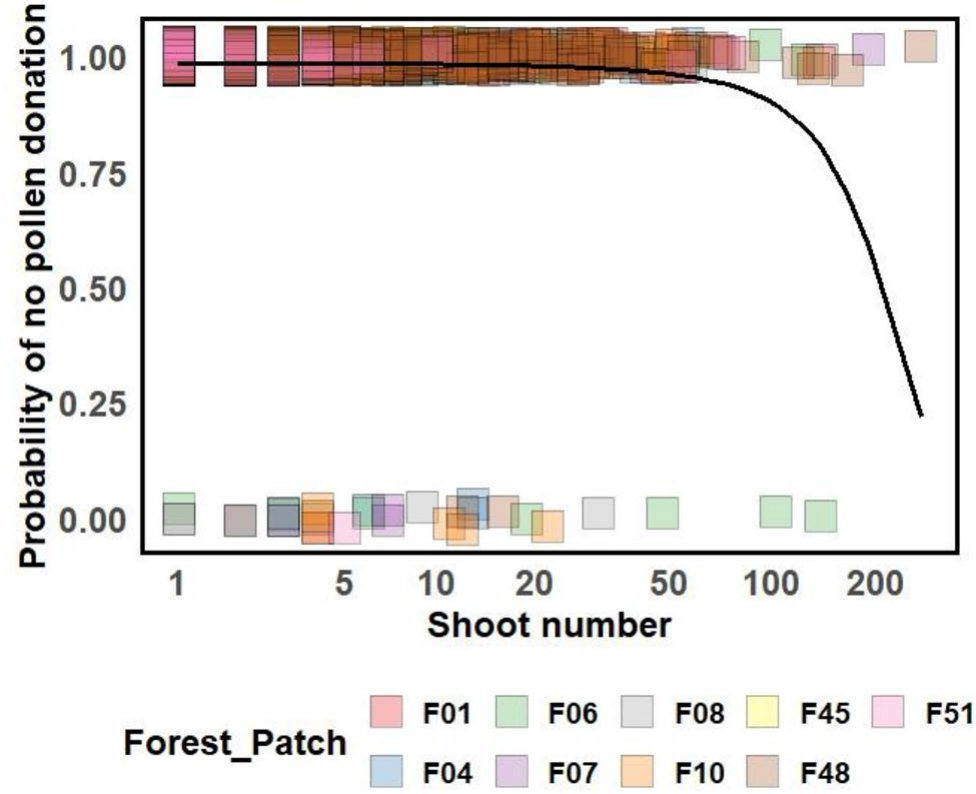
Effect of *P. multiflorum* shoot cluster size on the probability that a shoot cluster does not contribute pollen (predicted by the zero-inflation part of the GLMM; see Supplement 8 for the model of cluster size and distance to forest edge on pollen donation). The black line indicates the partial effect predicted by the model. Colored squares show whether a shoot cluster donated pollen (0) or not (1), with colors indicating the forest patch of origin. Squares are jittered vertically to show overlapping points. The x-axis represents shoot cluster size on a log-transformed scale (natural logarithm of 1 + shoot count, i.e., log1p)

The probability of a shoot cluster being an effective pollen donor increased with cluster size (Fig. 3, Supplement S8). There was a marginally significant interaction between shoot cluster size and distance to the forest edge (*p* = 0.088; Figure S8.1, Supplement S8). Specifically, for larger shoot clusters the number of assigned offspring was higher when these clusters were farther away from the forest edge, whereas this pattern was not observed for smaller shoot clusters.

### Landscape effects on *PF*_*within*_ and *A*_*r*_

**Fig. 4:**
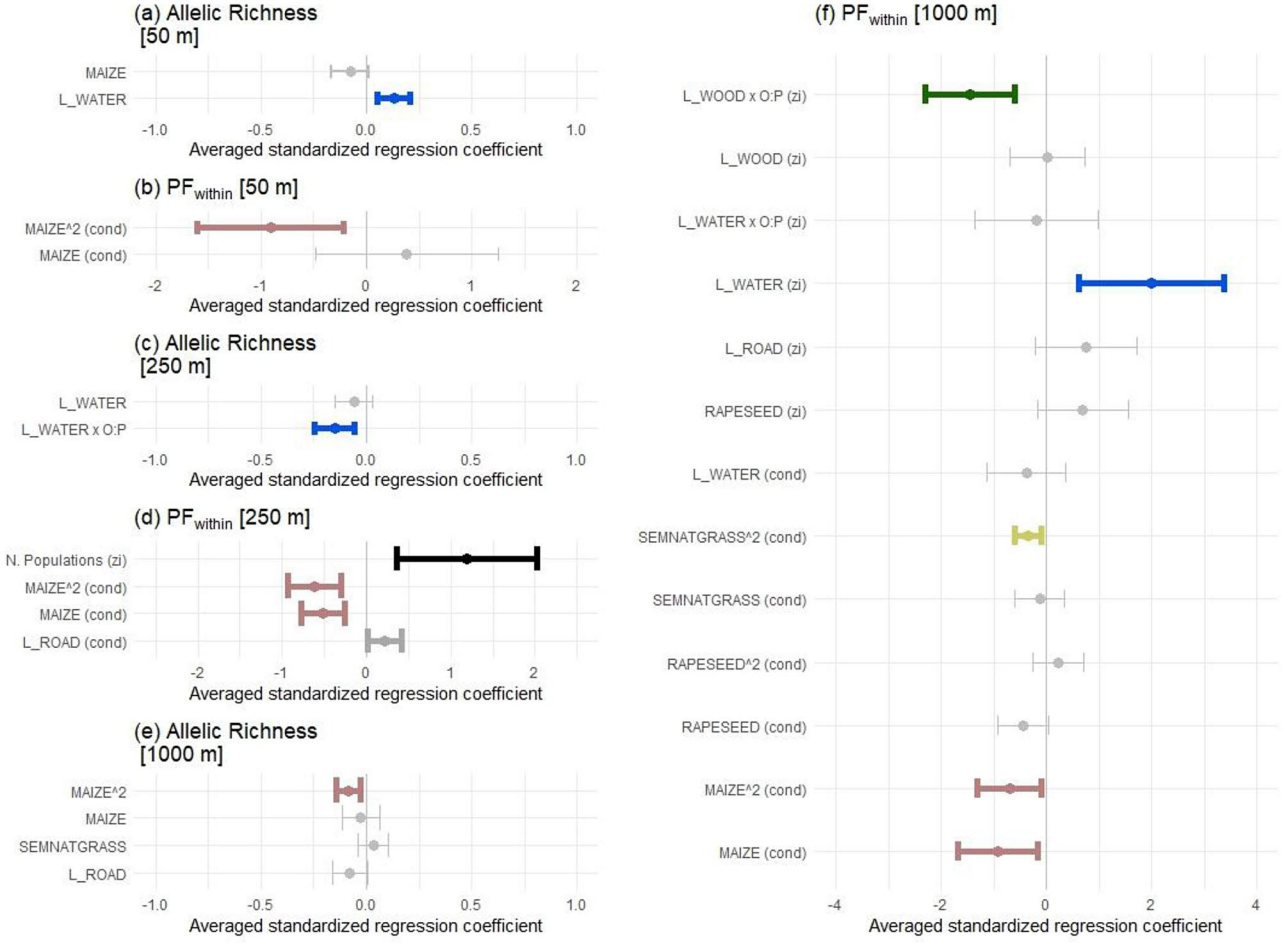
Landscape-composition effects on allelic richness (*A*_*r*_) (a, c, e) and *PF*_*within*_ (b, d, f) of *P. multiflorum* across three buffer radii (50 m, 250 m, 1000 m). Effects on *A*_*r*_ were modeled with LMM and on *PF*_*within*_ with GLMM with a conditional (cond) and zero-inflation part (zi); zi: probability of *PF*_*within*_ = 0; cond: strength of *PF*_*within*_. Shown are averaged standardized regression coefficients and their 95% confidence interval. Confidence intervals are colored if they do not overlap zero: brown for maize cover, yellow for semi-natural grassland, blue for water courses, green for woody linear elements, dark grey for roads and black for the number of *P. multiflorum* populations. Confidence intervals overlapping zero are shown in grey. For some linear landscape elements, interactions with their spatial orientation are indicated by “x O:P”. Overviews of the model averaging can be found in Supplement S10

**Fig. 5:**
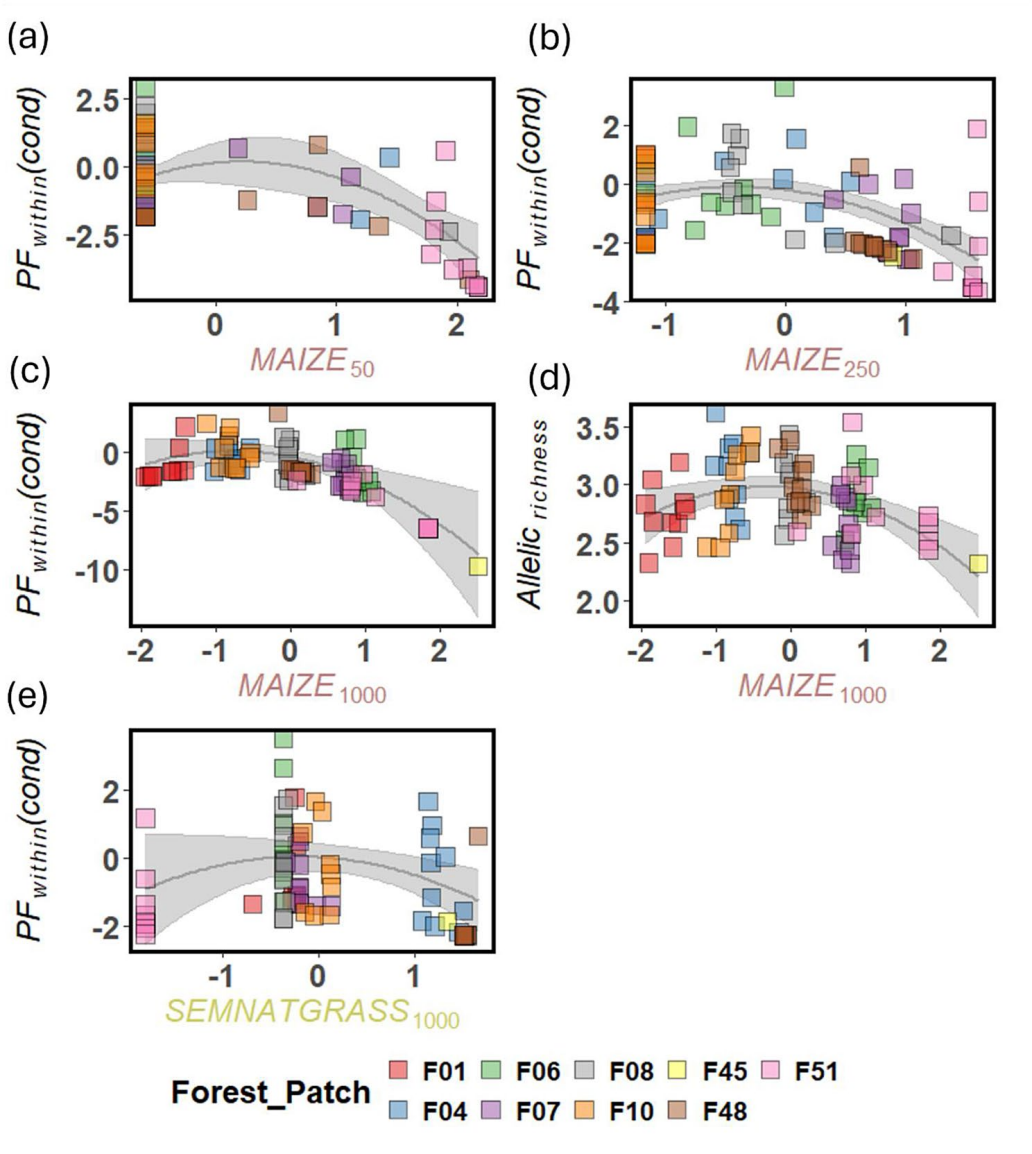
Linear-quadratic and unimodal effects of landscape metrics on *PF*_*within*_ and *A*_*r*_ of the forest herb *P. multiflorum*. Effects on *A*_*r*_ were modeled with LMM and on *PF*_*within*_ with GLMM with a conditional (cond) and zero-inflation part (zi). Shown are the partial effects of the specific explanatory variable extrecated from the model were it had its strongest detected effect. Filled squares represent residuals. The colors of the squares indicate the forest patch from which the pollen receptors originated. The complete models can be found in Table S11

The proportion of maize had significant effects on *PF*_*within*_ across all buffer radii, and on *A*_*r*_ at the 1000 m buffer radius (Fig. 4, Supplement 10). The relationship between maize cover and *PF*_*within*_ was unimodal at 50 m (Figs. 4b; Fig. 5a), and curvilinear at 250 m and 1000 m (Fig. 4d and f; Fig. 5b and c). In all three cases, *PF*_*within*_ initially increased slightly but then decreased as maize cover exceeded a certain threshold. For *A*_*r*_, maize cover had a unimodal effect at 1000 m buffer radius (Fig. 4e; Fig. 5d). The only significant effect of land-use types rich in floral resources was a unimodal effect of semi-natural grassland on *PF*_*within*_ at 1000 m buffer radius (Fig. 4f; Fig. 5e).

**Fig. 6:**
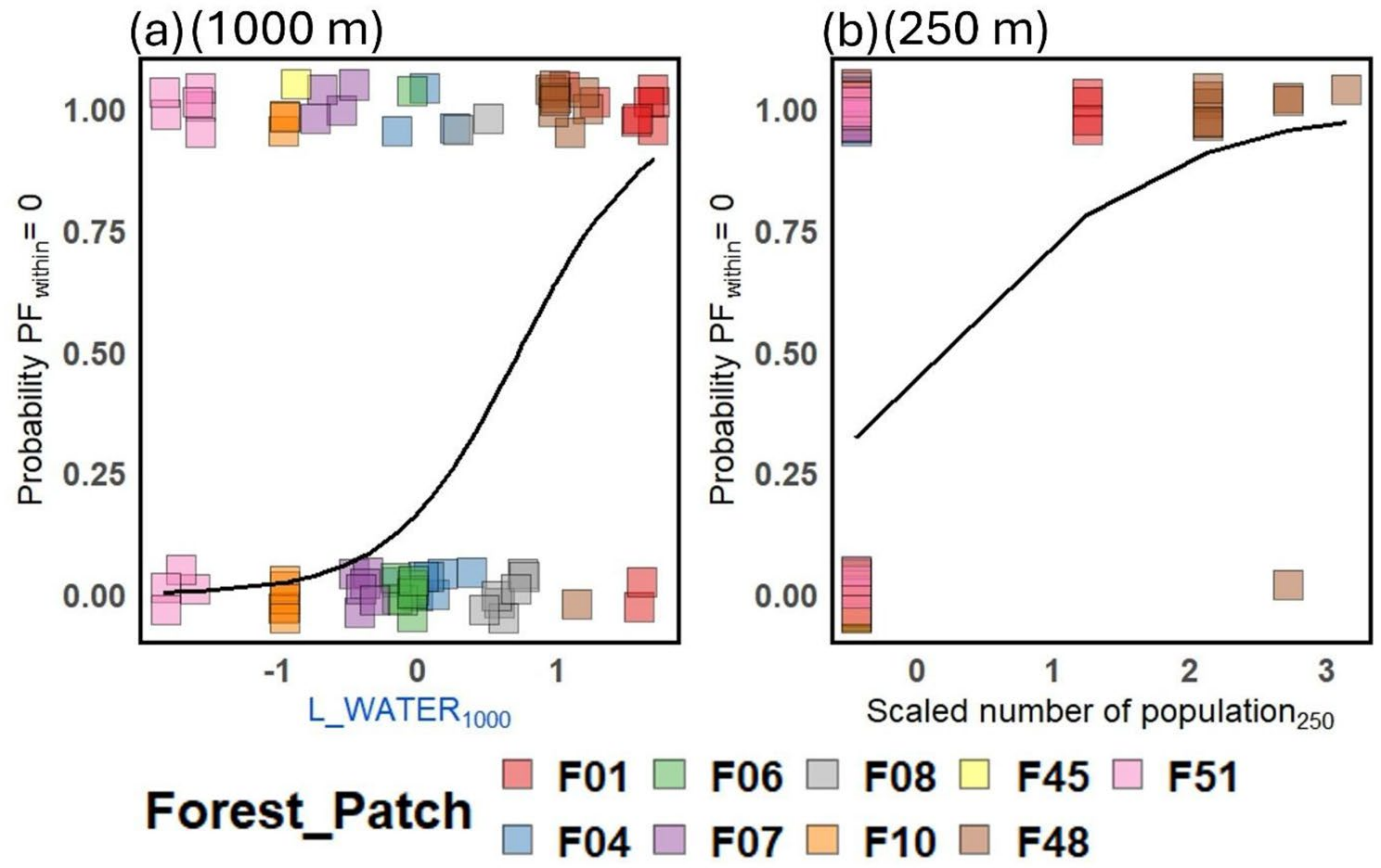
Effects on the probability of *PF*_*within*_ = 0 per *P. multiflorum* pollen receptor modeled by GLMM with a conditional (cond) and a zero-inflation (zi) part; (**a**) of the length of linear water courses at 1000 m buffer radius and (**b**) of the number of *P. multiflorum* populations at 250 m buffer radius. The black lines indicate the partial effect predicted by the model. Colored squares show whether a pollen receptor received pollen only from outside the forest patch (1) or partially from inside (0), with colors indicating the forest patches from which the shoot cluster originated. Squares are jittered vertically to show overlapping points. The complete models can be found in Table S11

**Fig. 7:**
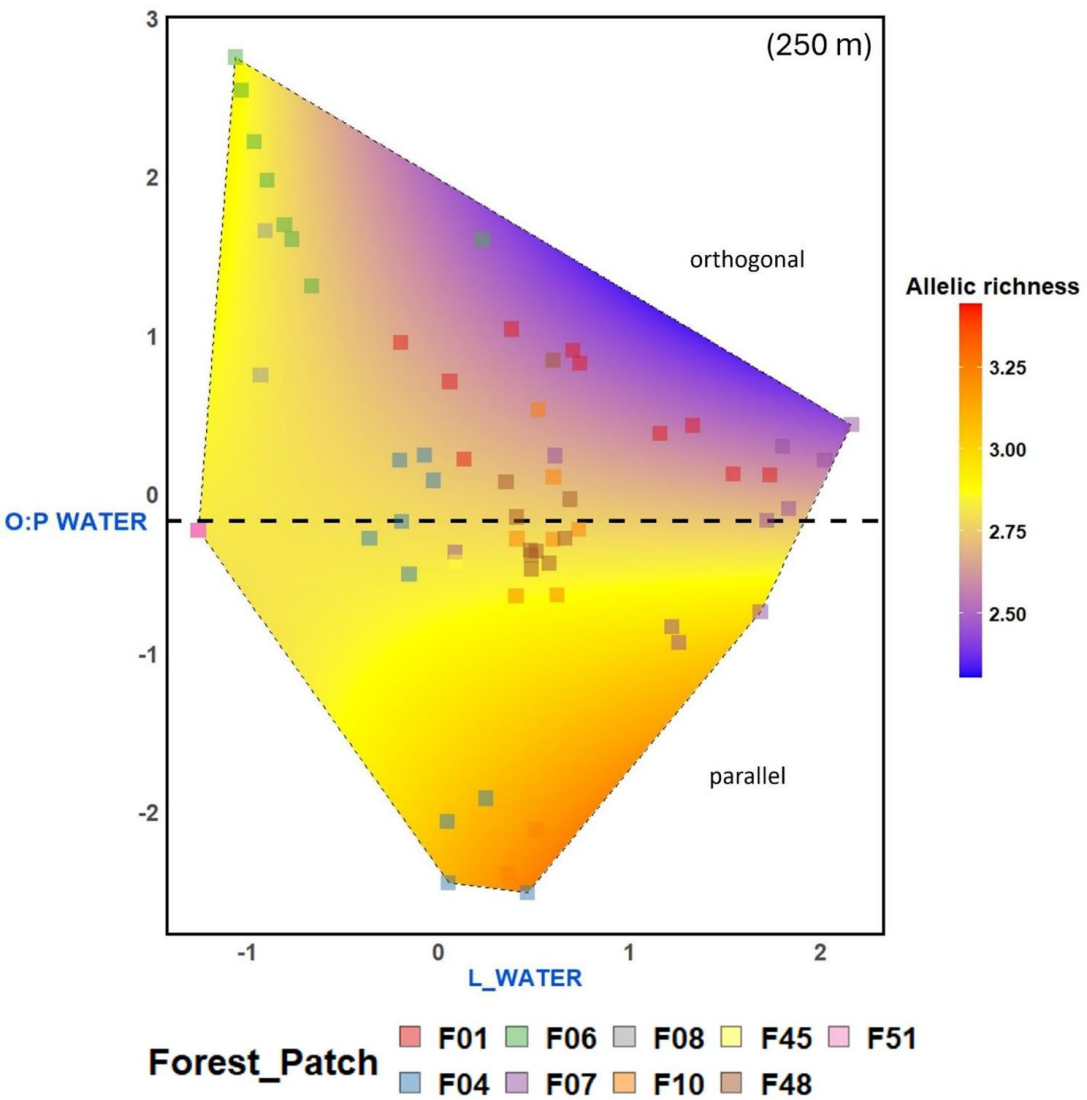
Interaction effect of linear landscape metrics and their spatial orientation in LMM on allelic richness (*A*_*r*_) of the offspring of *P. multiflorum* pollen receptor plants at 250 m buffer radius. The plot shows the partial effects of the specific explanatory variable extracted from the candidate model where it had its strongest effect. Filled squares represent raw data and colors indicate the forest patch from which the pollen receptors originated. The complete model can be found in Table S11

Linear landscape elements significantly affected both *PF*_*within*_ and *A*_*r*_ across all three buffer radii (Fig. 4). The total length of water courses had a positive effect on *A*_*r*_ at 50 m buffer radius (Fig. 4a) and increased the probability of *PF*_*within*_ = 0 per pollen receptor at 1000 m buffer radius (Fig. 4c; Fig. 6a). The total length of roads positively affected *PF*_*within*_ at 250 m buffer radius and marginally negatively affected *A*_*r*_ at 1000 m buffer radius (*p* = 0.069). In addition to these main effects, interactions between the length of linear elements and their spatial orientation were significant. At the 250 m buffer radius, *A*_*r*_ increased with the length of watercourses running parallel but decreased with the length of orthogonal ones (Figs. 4c, 7). At the 1000 m buffer radius, woody linear elements running parallel increased the probability that a pollen receptor received pollen exclusively from donors of other forest patches (zero-inflation part: Fig. 4f).

Finally, the number of *P. multiflorum* populations within the buffer zone significantly increased the probability of *PF*_*within*_ = 0 per pollen receptor (Fig. 4d; Fig. 6b).

## Discussion

Our results revealed a relatively low *PF*_*within*_, which indicates a high degree of pollen-mediated gene flow among *P. multiflorum* shoot clusters across forest patches, higher than anticipated in H1, where we expected that the majority of mating events happen within forest patches. We also found that shoot cluster size may lead to unequal contributions to pollen-mediated gene flow from different shoot clusters within a forest patch, which generally aligns with our expectations in H2. Furthermore, we identified both area-based land-use types and linear landscape elements as affecting *PF*_*within*_ and *A*_*r*_, broadly supporting our expectations in H3 and H4. However, our findings also suggest that the underlying mechanisms are more complex than initially hypothesized.

### Higher pollen-mediated gene flow among than within forest patches (H1)

Our results indicate that realized pollen-mediated gene flow events occur four times more frequently among forest patches than within them. In general, theories on fragmented plant populations predict that most mating occurs within habitat patches [3, 121]. However, considering the combination of the landscape-level foraging behavior of bumblebees and strict self-incompatibility of the forest herb species, this assumption does not necessarily hold true [75]. A likely explanation for the strong signal of among forest patch pollen-mediated gene flow from the pollinator perspective is the interpretation that the relevant bumblebee species establish traplines that include multiple forest patches within the agricultural landscape. Traplining behavior has been documented for different bumblebee species under both laboratory and field conditions [36–40]. Torres-Vanegas et al. [34] highlighted the contrasting effects of territorial versus traplining pollinator behaviors on pollen-mediated gene flow of *Helicona tortuosa*, with traplining pollinators predominantly mediating gene flow among forest patches. In our *P. multiflorum* populations, most observed relevant pollinators are bumblebees (Supplement Table S1), which are likely to exhibit some form of traplining behavior. Workers of *B. pascuorum*, which are among the most frequent flower visitors of *P. multiflorum* [90], forage equally across open landscapes and forests [91]. A single worker’s trapline might include movements both among and within forest patches. If traplines consists of 10 to 30 flower visits from a central location [38], as observed in *Bombus terrestris* [122], it is likely that foragers visit multiple forest patches, making only a few stops in each. This means that pollen receptors located at the end of a trapline receive an accumulated pollen load from all previously visited pollen donors [35, 123]. When a trapline includes multiple forest patches, the resulting mixed pollen load contains a substantial proportion of pollen originating from other forest patches [35]. Consequently, beginning with the second forest patch, every within-patch movement event among *P. multiflorum* individuals also contributes to pollen immigration from other forest patches.

The mechanism to avoid selfing in *P. multiflorum* likely reduces the number of potential mating patterns within forest patches. Traplining foraging by bumblebees alone would not be sufficient to explain the weak signal of within pollen-mediated gene flow that we detected for individuals in most of the forest patches. Near-neighbor foraging, which typically leads to fine-scale genetic structure, has been observed in other bumblebee-pollinated plants [54, 123]. Based on our field observations, in which foraging workers of *B. pascuorum* frequently moved among flowers of the same shoot or nearby plants, we would have expected a stronger signal of short-distance pollen movement. Indeed, the fact that offspring from the same pollen receptor regularly shared the same pollen donor (Figure S7.2) indicates that the worker movement between flowers of the same shoot is reflected in the mating patterns. The observation that multiple offspring from the same pollen receptors were sired by the same pollen donors suggests relatively high pollen carryover by the bumblebee workers, which is another important aspect influencing how foraging patterns shape plant mating pattern [124]. However, the self-incompatibility system of *P. multiflorum* appears to reduce the likelihood of successful seed set from pollen originating within a forest patch. This is supported by our findings that overall, all offspring were assigned to only 1.7% of the genotypes included as potential pollen donors, which is lower than expected. Moreover, an unexpectedly high proportion (47%) of pollen receptors had no offspring originating from pollen donors from their own forest patch. This suggests that, as a strictly outcrossing species, *P. multiflorum* should possess additional mechanisms that limit mating between spatially proximate and more closely related individuals within the same forest patch, a pattern also reported in other plant species [34]. For instance, the presence of self-incompatibility (S) alleles, as suggested by Wiberg et al. [125] or Wagenius et al. [126], and demonstrated in fragmented populations of *Rutidosis leptorrhynchoides* [127], may limit mating between genetically related individuals. Alternative mechanisms, such as pollen competition or female choice, which confer an advantage to pollen from outside the forest patch, might also operate in this system [128, 129]. This influence of self-incompatibility on the observed mating patterns is further supported by the finding that a substantial proportion (23%) of within-forest patch pollen-mediated gene flow events occurred over distances greater than 50 meters (Figure S7.3). Therefore, further research into the self-incompatibility mechanism of *P. multiflorum* is needed to more accurately estimate its effective population size, which may be substantially smaller than the number of flowering shoots would suggest.

Nevertheless, we need to be cautious when interpreting pollen immigration rates based on 1-*PF*_*within*_, as our method likely underestimates *PF*_*within*_. This is because the method is based on positive assignments of pollen donors located within the forest patch. As stated in the Materials and Methods section, the probability of a shoot cluster being monoclonal decreased to approximately 28% as the cluster size exceeded 20 shoots. Our sampling included a total of 118 out of 2134 shoot clusters with more than 20 shoots (Table S2.1), which were unevenly distributed across forest patches (mean number of shoot clusters with more than 20 shoots per forest patch: 13; min: 0 in F45; max: 45 in F48). We acknowledge that this methodological limitation may have resulted in some genotypes being missed within the sampled shoot clusters. This could lead to a slight overestimation of pollen influx and highlights the need for a better understanding of the ecology of shoot cluster-forming clonal forest herb species, including the relative proportions of shoots of sexual and vegetative origin. Complete genotyping of very large shoot clusters in future research would be particularly informative. However, even in cases where these clusters are not monoclonal, they typically consist of only a few different genotypes, far fewer than the number of shoots per cluster (Table S4.3 and S4.4). Moreover, among the 33 shoot clusters with more than one genotype that were included in the paternity analysis, no shoot cluster was assigned with more than one genotype. This suggests that increasing the sampling of genotypes per shoot cluster is unlikely to have resulted in the assignment of multiple effective pollen donors per shoot cluster. Therefore, despite the potential bias due to unsampled pollen donors and the implementation of five independent repetitions of offspring-pollen donor assignments to avoid overestimating pollen immigration rates, the observed pollen immigration rates still appear high. This is especially notable when compared to paternity analyses of closely related species such as the leaky self-incompatible *Convallaria majalis*, where 74.5% of offspring were assigned to pollen donors from their own populations [130].

### Non-random mating patterns within forest patches (H2)

There was evidence that mating patterns within a forest patch were non-random and likely influenced by the number of flowers provided by each *P. multiflorum* shoot cluster and its effect on bumblebee foraging behavior.

We found that larger shoot clusters had a higher probability of functioning as effective pollen donors (Fig. 3). As discussed in the section above, the larger shoot clusters contained some genotypes that were not covered by our sampling, indicating that the detected effect of the shoot cluster size might be even stronger. Similar effects of shoot cluster size have been reported in other clonal plant species, such as *Saggitaria latifolia* [131] or *Convallaria majalis* [130]. The greater number of flowers in larger clones might also result in a longer flowering period further increasing their chances of mating with more shoot clusters [46, 132]. Furthermore, bumblebee workers from a greater number of species might visit shoot clusters if they increase in size [94]. The number of assigned pollen donors in insect-pollinated plants tends to remain relatively stable across years [133]. This suggests that if a few large shoot clusters repeatedly dominate mating each year, their influence will be traceable in the genetic structure of the species over time [52]. In previous studies on *P. multiflorum*, we found a significant heterozygote excess, which could be explained by this dominance of few pollen donors [10, 90].

### Influences of different types of semi-natural habitats on pollen-mediated gene flow within landscapes with low floral-resource availability (H3)

Our study indicated that in maize-dominated landscapes, small semi-natural grassland patches influence mating patterns of forest plants with mechanisms other than simply distracting pollinators. The curvilinear, negative effects of maize cover on *PF*_*within*_ at both the 250 m and 1000 m scales align with our expectation in H3 that land-use types with few floral resources increase the relative value of forest herbs, thereby enhancing gene flow among their populations (Fig. 5b and c). As a dominant crop covering large areas (Fig. 1, Table S6), maize reduces overall floral resource availability.

In contrast, for land-use types rich in floral resources, we generally expected the opposite effect under H3, i.e., that they would distract pollinators from moving among forest patches. The unimodal effect of semi-natural grassland at the 1000 m buffer radius only partially supported this hypothesis. While *PF*_*within*_ initially increased with greater semi-natural grasslands cover (indicating reduced pollen immigration rates), it then declined at higher levels of cover (Fig. 5e). Whether plant species from different habitats compete for or facilitate pollination services depends on their quantity and spatial distribution [134]. In a previous study including three landscape windows, we observed variation in pollinator movement among forest patches that likely reflects different levels of distraction depending on the extent of semi-natural grassland [71]. That study featured greater semi-natural grassland cover variation and higher overall cover than the current one, which focused on the German landscape window, where semi-natural grasslands were mostly small and scattered (Fig. 1, Table S6). Smaller patches likely offer fewer floral resources, requiring pollinators to visit multiple patches [6, 135]. Here, spillover among semi-natural grassland and forest patches may outweigh the distraction effect. Bumblebees will prefer foraging in landscapes with high floral-resource availability [30], explaining the initial *PF*_*within*_ increase in forest patches surrounded by few semi-natural grassland patches compared to those surrounded by none in a 1000 m radius (Fig. 5e). Furthermore, if bumblebee workers visit both grassland and forest, this would add additional plant species to their pollen load, potentially reducing the probability of effectively transporting pollen between two forest patches due to an increase in heterospecific pollen [74]. For workers of *Bombus terrestris*, an average pollen diversity of 2.5 species has been reported [75]. While evidence suggests that pollen loads of *B. pascuorum* workers are more diverse than those of *B. terrestris* [136], they remain relatively flower-constant [137]. This means that a single worker is unlikely to add a high number of additional species during a single foraging flight, but will reach a certain, moderate level of pollen diversity. At higher semi-natural grassland cover, the higher number of small semi-natural grassland patches may increase the chances of acting as stepping stones among forest patches, facilitating pollen-mediated gene flow among forest patches and reducing *PF*_*within*_.

Beyond floral resources, semi-natural grasslands also offer nesting habitat for many bumblebee species, often increasing forager abundances in the landscape [23, 138] and enhancing pollination services for adjacent wild plant populations [139]. This spillover could further explain the initial rise in *PF*_*within*_, as more workers may contribute to the pollen-mediated gene flow within a forest patch. However, whether higher pollinator abundances lead to increased pollen-mediated gene flow among forest patches depends on whether the workers forage across multiple semi-natural habitat patches as discussed above [5, 140]. Overall, our findings on the effects of semi-natural grassland highlight the importance of understanding the conditions under which semi-natural elements in agricultural landscapes support or potentially compete for pollinators involved in pollination and pollen-mediated gene flow [134, 141, 142].

### Decoupled effects of maize cover on pollen immigration rates and genetic diversity of the offspring (H3)

We also found that higher pollen-immigration rates in landscapes dominated by land-use types poor in floral resources do not necessarily increase the genetic diversity of the offspring per pollen receptor. At the 1000 m buffer radius, maize cover significantly affected both *PF*_*within*_ and *A*_*r*_ (Figs. 4e and 4f), yet these metrics were uncorrelated (|*r*| = 0.08). While higher pollen immigration rates are generally expected to promote offspring genetic diversity [3, 34], our findings suggests that their responses to maize cover may diverge. The pollen immigration rate reflects the quantity of pollen influx, but not its quality. In contrast, the genetic diversity of the offspring depends on both the number and diversity of contributing pollen donors. This number can be high even for mating within a habitat patch [143], but allelic richness should be particularly sensitive to pollen-mediated gene flow over longer distances, where the probability of introducing novel alleles into the gene pool is greater [144]. Following the logic of traplining behavior in bumblebees, as discussed above, 1-*PF*_*within*_ captures the overall pollen immigration rate, but it does not reflect the number of traplines, the genetic diversity or the number of individual donors within a trapline, or the stability of the trapline, i.e., how frequently foraging routes change [35, 145]. In resource-poor landscapes, bumblebees may initially respond by establishing more frequent, longer and more targeted traplines among forest patches [68, 146, 147], thereby increasing both pollen immigration rates and genetic diversity of the offspring. However, beyond a certain threshold, landscapes may become too unattractive, and bumblebees may avoid flying across large expanses of resource-poor areas. As a result, fewer traplines with fewer donors may be established, reducing genetic diversity even if pollen immigration rates increase due to the higher relative value of the forest herbs [47]. A similar decoupling of pollen immigration rates and genetic diversity has been observed in *Swietenia humilis*, where spatial isolation led to lower genetic diversity but did not affect pollen immigration rates [106].

### Role of spatial configuration for effects of linear landscape elements on pollen immigration rates (H4)

Our results indicate that the spatial configuration of specific linear landscape elements affects bumblebee navigation and, consequently, pollen immigration rates of *P. multiflorum* populations. Overall, the observed effects of linear elements align with our expectations stated in H4 and can be interpreted as influences of these landscape elements on bumblebees’ navigation [61]. We found that the orientation of water courses at the 250 m buffer radius (Fig. 4c) and of woody corridors at the 1000 m buffer radius on the probability of *PF*_*within*_ = 0 (Fig. 4f) determined whether these elements facilitated or hindered pollen immigration into *P. multiflorum* populations. As expected, elements oriented toward the pollen receptors increased *A*_*r*_ (Water courses at 250 m; Fig. 7) or decreased *PF*_*within*_ (Woody corridors at 1000 m), while those oriented orthogonally had the opposite effects. This pattern aligns with findings from a previous study, in which genetic diversity increased with the length of water courses oriented towards populations and decreased with the length of orthogonally oriented water courses across 36 populations of *P. multiflorum* [107]. At the 1000 m landscape scale, Klaus et al. [78] demonstrated the dual function of hedgerows as either channel or barrier for the movement of pollinators using fluorescent dye analysis in the bee-pollinated *Centaurea cyanus*. We hypothesized that all three types of linear landscape elements considered would act as navigational aids, potentially exhibiting both barrier and channeling effects. Despite the general trend, we observed distinct effects among the element types, likely because their spatial configuration within the agricultural landscape was not random but followed certain rules. For instance, longer roads typically connected settlements and did not point towards forest patches (Figure S6.2b and c), which could explain the positive effect of road length on *PF*_*within*_ at the 250 m buffer radius (Fig. 4d). This barrier effect of orthogonally oriented roads on bumblebee foraging has been reported in earlier studies [148]. The positive effect of water course length on the probability *PF*_*within*_ = 0 (Fig. 4f; Fig. 6b) may be related to the positive correlation between the water course length and the number of *P. multiflorum* populations within a 1000 m buffer (|r| = 0.63) (Table S9.5), reflecting that several forest patches occupied by *P. multiflorum* were located along continuous water courses (Fig. 1). Stronger pollen-mediated gene flow among spatially isolated plant populations located along rivers has been demonstrated before [149]. The positive effect of water courses on allelic richness within the 50 m buffer also suggests a similar mechanism (Fig. 4a). This effect, however, should be interpreted with caution and primarily as an indication of the presence or absence of linear water courses within a 50 m distance of the pollen receptor, rather than an effect of their length, as 52 out of 79 pollen receptors had a value of zero. Future analyses of the effects of linear landscape elements should incorporate aspects of their configuration, such as whether these elements are oriented towards forest patches and whether they pass by at greater distances.

## Conclusion

Our study suggests that strictly outcrossing wild plant populations embedded within agricultural landscapes can exhibit high levels of gene exchange among populations. This pattern is likely driven by the traplining behavior of bumblebee pollinators, which visit multiple populations during a single foraging bout, in combination with the plants’ strict self-incompatibility. For both the studied forest herb species and their bumblebee pollinators, future research should investigate the underlying mechanisms in more detail. As our study was restricted to a single plant species in a single landscape due to the intensity of sampling, investigating the mating patterns of additional forest herb species in agricultural landscape, particularly those with contrasting mating systems, would further broaden our understanding of the functional connectivity of wild plants species embedded in these environments.

At the landscape scale, our results indicate that bumblebee-pollinated plant populations form networks connected through pollen-mediated gene flow, suggesting a relatively high resilience to fragmentation at the spatial scale studied here. However, self-incompatibility mechanisms may strongly restrict the number of effective pollen donors within populations, which could become problematic if forest habitats become further isolated due to ongoing habitat loss and fragmentation.

Our findings further highlight that the distribution of floral resources and the spatial orientation of linear landscape elements are important drivers of functional connectivity in bumblebee-pollinated wild plants. Conservation programs aiming to enhance functional connectivity of wild plant populations in floral-resource-poor agricultural landscapes should therefore consider both the amount and orientation of linear water courses, as well as the positive and negative interactions between semi-natural habitats that influence pollinator foraging.

Finally, our study underscores the importance of considering both quantitative and qualitative aspects of pollen influx when analyzing landscape effects. Landscape-effects may increase gene exchange without necessarily introducing new alleles.

## Electronic supplementary material

Below is the link to the electronic supplementary material.

Supplementary Material 1

Supplementary Material 2

Supplementary Material 3

Supplementary Material 4

Supplementary Material 5

Supplementary Material 6

## Data Availability

The allele table of P. multiflorum individuals (Table S4.2 Allele_table.xlsx), the information on the positions of the shoot clusters (Table S2.3 All_pollen_donors.xlsx), and the landscape metrics (Landscape_Metrics.R) used can be found in the supplementary material. All steps of the R analysis are reported in the Methods section and can be provided on request.

## Abbreviations

H1-H4: Hypotheses 1 to 4
MLG: Multi-locus genotypes
PF_within_: Proportion of offspring per pollen receptor that could be assigned to pollen donors from the own forest patch.
A_r_: Allelic richness
IACS: European Integrated Administration and Control System
LMM: Linear mixed models
GLMM: Generalized linear mixed models
Cond component: Conditional component
Zi component: Zero-inflation component
AICc: Akaike Information Criterion corrected for small sample size
ML: Maximum likelihood
