# Supplementary material for: Greater pollen-mediated gene flow among than within populations of a bumblebee-pollinated forest herb despite habitat fragmentation
Source: Mov Ecol. 2026 May 30;14:36. doi: 10.1186/s40462-026-00664-8 (PMC13224658; doi:10.1186/s40462-026-00664-8)
Supplement: Supplementary file 1 — Supplementary Material 1 [file 40462_2026_664_MOESM1_ESM.docx]

**Supplement: Greater pollen flow among than within populations of a bumblebee-pollinated forest herb despite habitat fragmentation**

[Supplement 1: Shoot clusters of *Polygonatum multiflorum* and their bumblebee pollinators 2](#_Toc202882416)

[Supplement 2: Overview over included samples 4](#_Toc202882417)

[Supplement 3: Stratification and germination 9](#_Toc202882418)

[Supplement 4: Genotyping, quality and assessment of clonality 10](#_Toc202882419)

[Supplement 5: Information on quality of paternity analysis 14](#_Toc202882420)

[Supplement 6: Landscape metrics 15](#_Toc202882421)

[Supplement 7: Distribution of pollen flow measures 18](#_Toc202882422)

[Supplement 8: Model outcome H2 21](#_Toc202882423)

[Supplement 9: Collinearity among included variables 22](#_Toc202882424)

[Supplement 10: Outcome of Model averaging for H3 and H4 25](#_Toc202882425)

# Supplement 1: Shoot clusters of *Polygonatum multiflorum* and their bumblebee pollinators

| **A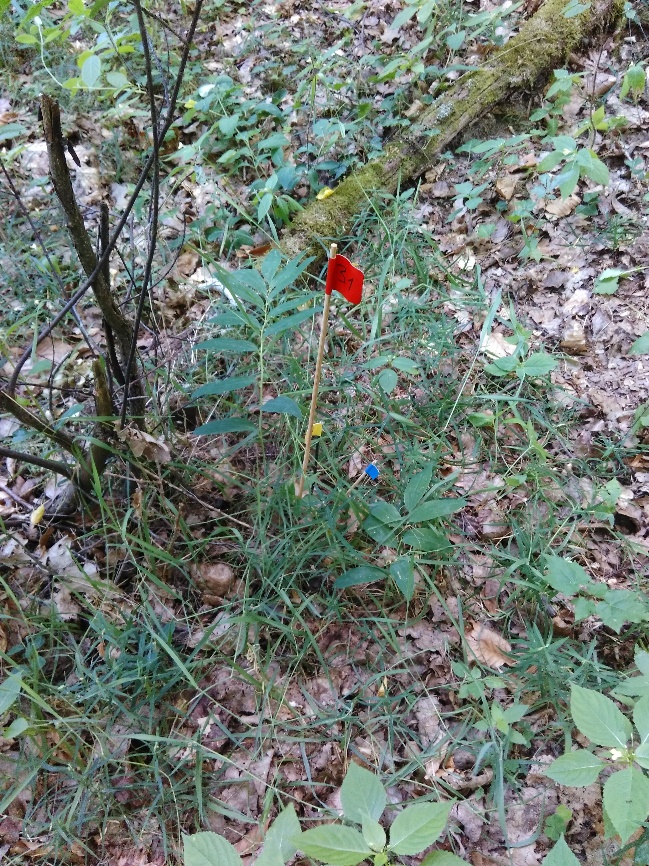** | **B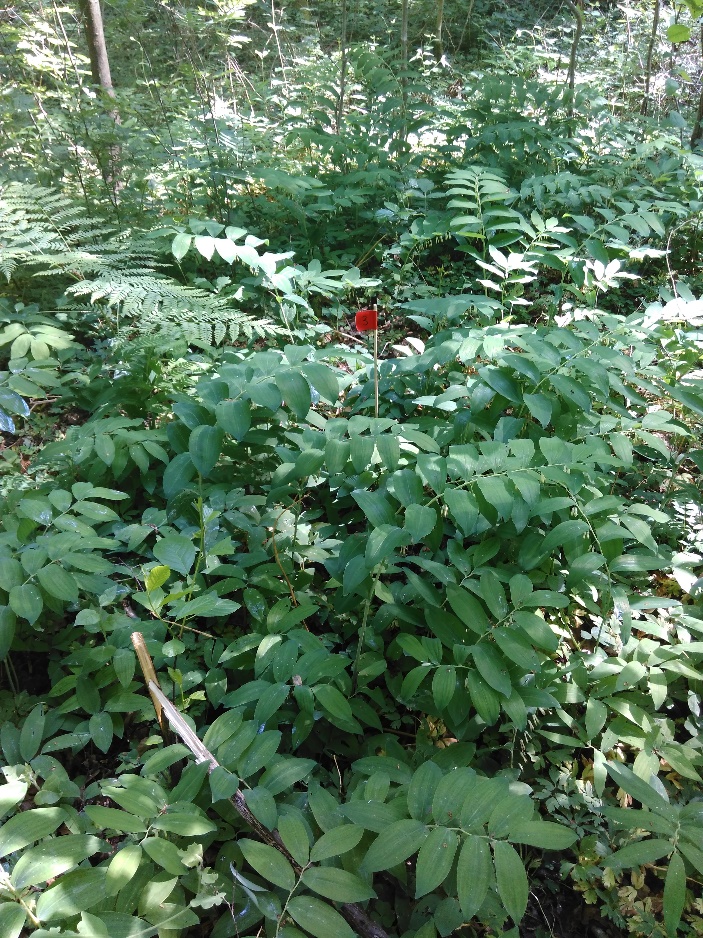** |
| --- | --- |
| **C** | **D** |
| **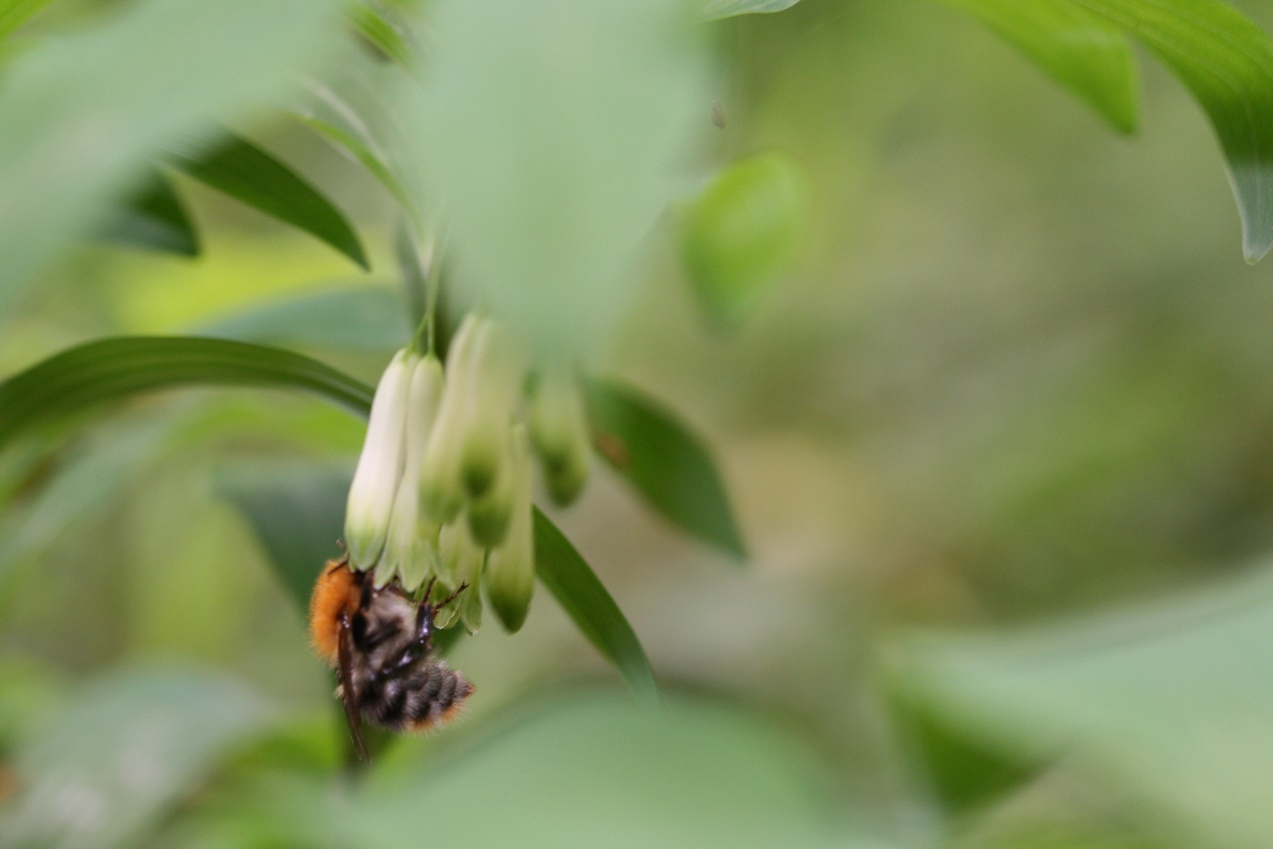** | **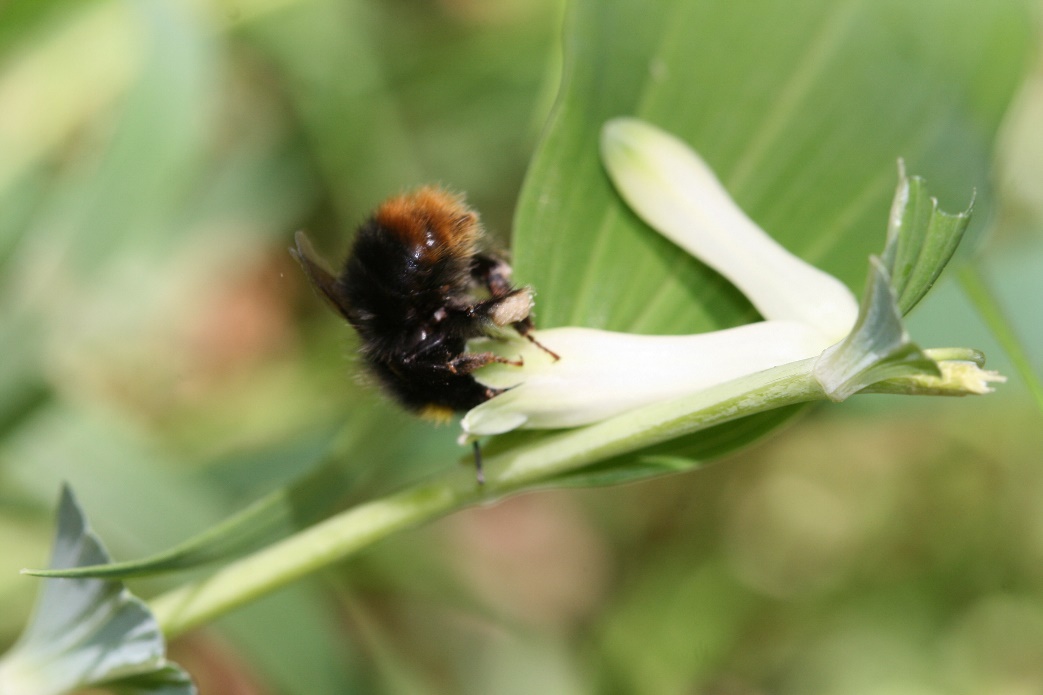** |

**Figure S1:** Pictures A and B: Shoot clusters of *P. multiflorum* with only a few flowering shoots (A) and multiple shoots (B); pictures C and D: Flower visits of *Bombus pascuorum* (C) and *Bombus pratorum* (D).

**Table S1:** During 52 hours of flower observation of *P. multiflorum*, we observed between one and four different bumblebee species per forest patch, out of a total of five species. Additionally, we also recorded other wild bee flower visitors from the genus *Anthophora*.

| *Forest patch* | Number individuals  *B. pascuorum* | Number individuals  *B. pratorum* | Number individuals  *B. hortorum* | Number individuals  *B. lapidarius* | Number individuals  *B. hypnorum* | *Number Bombus species* | *Anthophora spec.* |
| --- | --- | --- | --- | --- | --- | --- | --- |
| F01 | 5 | 5 | 0 | 1 | 0 | 3 | 0 |
| F04 | 4 | 0 | 0 | 0 | 0 | 1 | 0 |
| F06 | 7 | 3 | 1 | 0 | 0 | 3 | 1 |
| F07 | 13 | 8 | 0 | 1 | 0 | 3 | 0 |
| F08 | 7 | 4 | 0 | 0 | 0 | 2 | 0 |
| F10 | 5 | 9 | 0 | 1 | 1 | 4 | 0 |
| F45 | 1 | 0 | 0 | 0 | 0 | 1 | 0 |
| F48 | 6 | 9 | 3 | 1 | 0 | 4 | 1 |
| F51 | 5 | 0 | 0 | 0 | 0 | 1 | 0 |

# Supplement 2: Overview over included samples


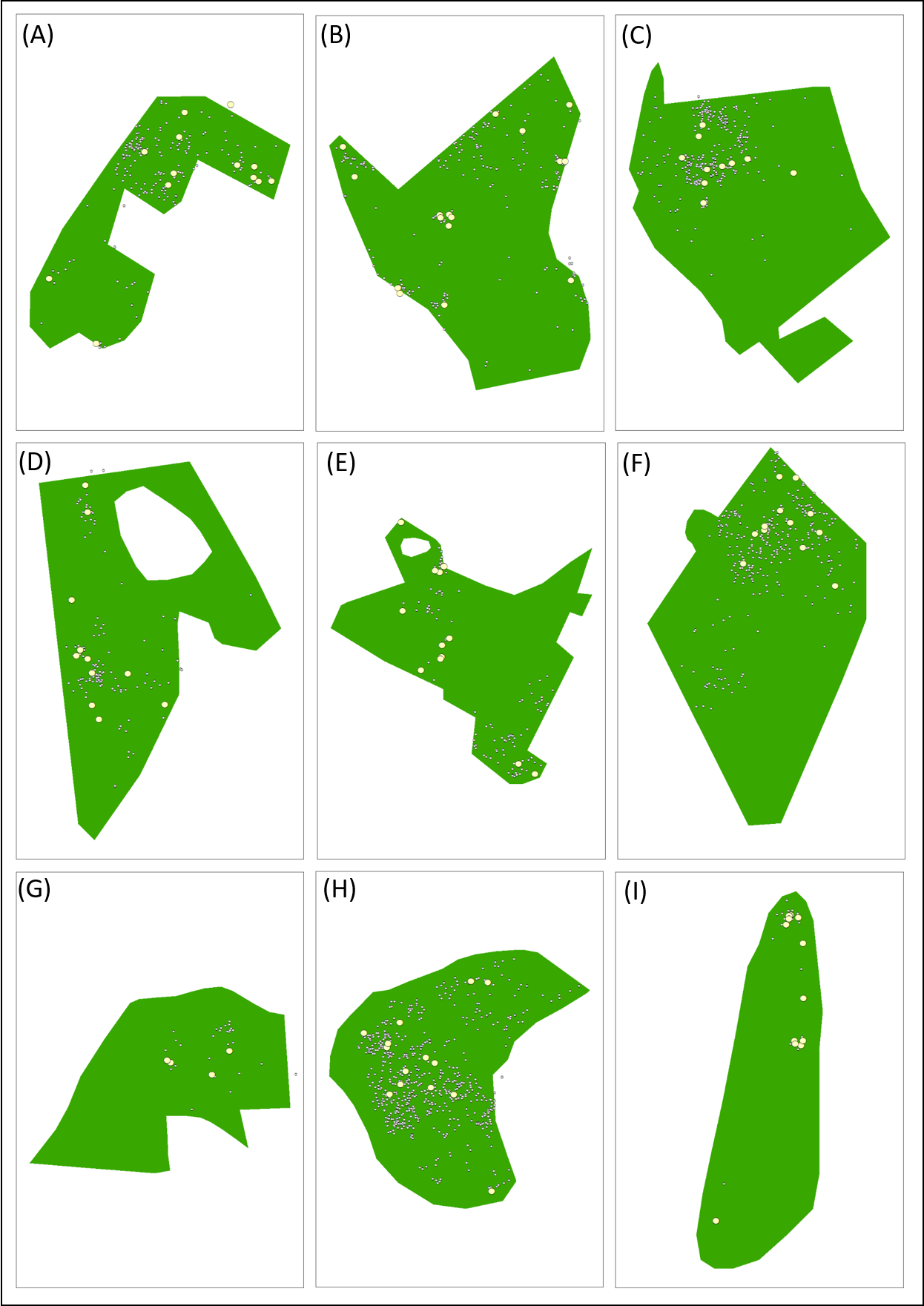


**Figure S2:** This figure shows maps of the nine forest patches (depicted in green) a-l: F01, F04, F06, F07, F08, F10, F45, F48, F51 included in this study. Yellow dots represent the plants selected as pollen receptors, while grey dots indicate all potential pollen donors.

**Table S2 Column descriptions:** The attached Excel file, “Table S2”, provides the exact number of samples involved at each step of the analysis. For clarity, we divided the study process into four steps, organized as follows: **Step 1:** Sampling and germination; **Step 2:** Quality and clonality checks, **Step 3:** Paternity analysis, **Step 4:** Samples used for testing H1, H3 and H4. Below, the column-descriptions table explains the content of each column of Table S2.

| Column name | Description |
| --- | --- |
| Step 1: Sampling | This step is based on the dataset AllFatherPlantsData.xlsx, which is available on Dryad via (will be added before publication) |
| n sampled shoot cluster | The total number of shoots within each forest patch. All sampled in 2018. For each cluster, the number of shoots was documented. See Figure S2 for the spatial distribution of the shoot clusters. |
| n shoot cluster = 1 shoot | Number of all single shoots from each forest patch that were sampled in spring 2018. |
| n shoot cluster 2-9 shoots | Number of all shoot clusters with two to nine shoots from each forest patch sampled in spring 2018. |
| n shoot cluster 10-19 shoots | Number of all shoot clusters with ten to 19 shoots from each forest patch sampled in spring 2018. |
| n shoot cluster >= 20 shoots | Number of all shoot clusters with more than 19 shoots sampled from each forest patch sampled in spring 2018. |
| Proportion of shoot cluster = 1 shoot | Proportion of single shoots among the n sampled shoot clusters per forest patch. |
| Proportion of shoot cluster 2-9 shoots | Proportion of clusters with two to nine shoots among the n sampled shoot clusters per forest patch. |
| Proportion of shoot cluster 9-19 shoots | Proportion of shoot clusters with ten to 19 shoots among the n sampled shoot clusters per forest patch. |
| Proportion of shoot cluster >= 20 shoots | Proportion of shoot clusters with more than 19 shoots among the n sampled shoot clusters per forest patch. |
| Step 2 Quality and clonality checks | This step is based on the dataset Allele_table.xlsx, which is available on Dryad via …. |
| n successfully genotyped samples | Number of leaf and rhizome samples successfully genotypes after DNA isolation. |
| n successfully genotyped leaf samples as pollen donors | Number of leaf samples, successfully genotyped after DNA isolation, that were considered as pollen donors. |
| n successfully genotyped leaf samples for within shoot clusters | Number of leaf samples, successfully genotyped after DNA isolation, that were used for the clonality test within the shoot clusters. |
| n successfully genotyped leaf samples as pollen receptors | Number of leaf samples, successfully genotyped after DNA isolation, that were considered as pollen receptors. |
| n successfully genotyped rhizome samples genotyped as offspring | Number of rhizome samples, successfully genotyped after DNA isolation, that were considered as pollen receptors. |
| n samples removed from n successfully genotyped samples because of missing values <1 | Number of leaf and rhizome samples that were removed because of to many missing values. |
| n samples included in clonality test among shoot clusters | Number of leaf and rhizome samples included in the test of clonality among shoot clusters. |
| n distinct MLGs | Number of distinct multilocus genotypes identified in the clonality test among shoot clusters. |
| n identified clones among shoot clusters | Number of clones identified in the clonality test among shoot clusters. |
| n shoot clusters included in clonality test within shoot clusters | Number of shoot clusters that were included for the clonality test within shoot clusters (see Table S4.2 and S4.3 for details). |
| n genotyped leaf samples included in clonality test within shoot clusters | Number of all multilocus genotypes included in the clonality test within shoot clusters (see Table S4.2 and S4.3 for details). |
| n distinct identified MLGs in clonality test within shoot clusters | Number of distinct multilocus genotypes included in the clonality test within shoot clusters (see Table S4.2 and S4.3 for details). |
| n additional genotypes from clonality test within shoot clusters | Number of additional distinct multilocus genotypes identified through the within shoot cluster clonality test. |
| n all samples after Step 2 | Number of remaining genotypes after the checks of step 2^1-9^. |
| Step 3 Paternity analysis |  |
| n MLGs included in paternity analysis | Total number of all multilocus genotypes that were included in the paternity analysis. |
| n potential pollen donor MLGs | Number of all multilocus genotypes that were included in the paternity analysis as potential pollen donors (including pollen receptors). |
| n pollen receptors MLGs | Number of all multilocus genotypes that were included in the paternity analysis as pollen receptors. |
| n offspring MLGs | Number of all multilocus genotypes that were included in the paternity analysis as offspring with known pollen receptors. |
| Step 4 Data analysis |  |
| n pollen receptors included in testing H1, H3, H4 | Number of pollen receptors with more than five offspring, for which *PF_within_* and allelic richness were calculated. |
| n shoot clusters included in testing H2 | Number of included shoot clusters with known shoot numbers. |

**Samples removed in Step 2: Quality and clonality checks**

^1^ **F01:** **Removed because of missing values > 1:** 5 pollen donors, **removed due to clonality test among shoot clusters:** 18 pollen donors

^2^ **F04:** **Removed due to clonality test among shoot clusters:** 46 pollen donors, 1 offspring,

^3^ **F06:** **Removed because of missing values > 1:** 2 pollen donors, 1 offspring, **removed due to clonality test among shoot clusters:** 55 pollen donors, **removed due to mismatches between pollen receptor and offspring > 2:** 1 offspring

^4^ **F07: Removed because of missing values > 1:** 1 offspring, **removed due to clonality test among shoot clusters:** 16 pollen donors

^5^ **F08: Removed due to clonality test among shoot clusters:** 8 pollen donors, 1 offspring

^6^ **F10: Removed because of missing values > 1:** 2 offspring, 1 pollen donors, **removed due to clonality test among shoot clusters:** 40 pollen donors, 1 offspring

^7^ **F45: Removed because of missing values > 1:** 1 offspring, **removed due to clonality test among shoot clusters:** 4 pollen donors, 2 offspring

^8^ **F48: Removed due to clonality test among shoot clusters:** 73 pollen donors, 1 offspring

^9^ **F51: Removed because of missing values > 1:** 1 offspring, **removed due to clonality test among shoot clusters**: 5 pollen donors, 1 mother, 5 offspring, **removed due to mismatches between pollen receptor and offspring > 2:** 1 offspring

# Supplement 3: Stratification and germination

| **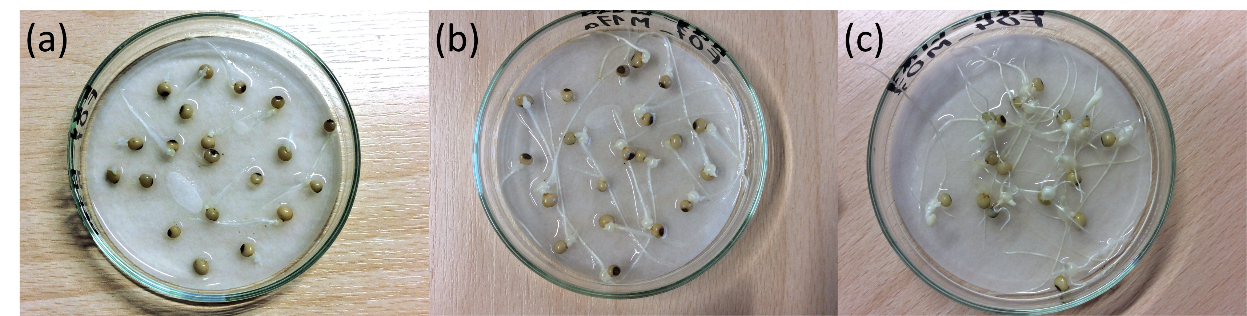** |  |  |
| --- | --- | --- |

**Figure S3:** Photographs after stratification: (a) 3.5 weeks, (b) 5 weeks, and (c) 10 weeks.

**Protocol:**

1. We extracted seeds from the fruits and thoroughly cleaned them from the fruit pulp.
2. We included in total 1442 seeds in the stratification and germination procedure. They originated from 91 different pollen receptors (6 – 14 pollen receptors per forest patch; 1 to 46 seeds per pollen receptor; mean: 17)
3. We placed up to 20 seeds per pollen receptor plant on wet filter paper (soaked with distilled water) on Petri dishes with a diameter of 88 mm. The Petri dishes were covered with a lid to reduce evaporation.
4. We exposed the seeds to a cold-wet stratification, i.e., we placed the Petri dishes in a climate chamber at 5°C for six weeks and kept the filter paper wet during this time.
5. After six weeks, the Petri dishes were kept in a closed box at room temperature (around 20°C) for another seven weeks. The filter paper was kept moist during this time.
6. We obtained a total number of 1006 seedlings, which indicates a germination rate of 0.70.

# Supplement 4: Genotyping, quality and assessment of clonality

**Table S4.1:** The table describes the seven different microsatellite primers used in this study. It reports the size ranges in base pairs (bp), the number of alleles and genotypes, the amount of missing values and pollen receptor-offspring mismatches, as well as the genotyping error rates based on 10 % repetition on receptor-offspring mismatches.

| Primer name | Range  [bp] | Alleles  [No.] | Genotypes  [No.] | Error rate based on 10% repetition | Receptor-offspring mismatch or NA  [No.] | Percentage of missing value  [%] | Error rate based on Receptor-offspring mismatches |
| --- | --- | --- | --- | --- | --- | --- | --- |
| Pmu024^1^ | 125-129 | 2 | 2 | 0.61 | 0 | 0 | 0 |
| Pmu091^1^ | 243-276 | 12 | 53 | 0.31 | 2 | 0.19 | 0.21 |
| Pc33^2^ | 233-277 | 22 | 164 | 0.31 | 8 | 0.34 | 0.84 |
| Pc25^2^ | 215-263 | 15 | 67 | 0 | 4 | 0.49 | 0.42 |
| Pmu008^1^ | 187-190 | 2 | 4 | 1.22 | 0 | 0.68 | 0 |
| Pmu373^1^ | 100-106 | 2 | 4 | 0.92 | 0 | 0.15 | 0 |
| Pt09^2^ | 126-216 | 42 | 448 | 1.22 | 9 | 0.52 | 0.95 |

^1^ Newly developed for this study

^2^ Developed for *P. cyrtonema* by Cheng et al. (2010) and for *P. filipes* by Liu et al. (2010).

**References**

Cheng WJ, Liu TT, Wu HL, Zhou SB, Xuan SQ, Zhu GP (2010) Isolation and characterization of twelve polymorphic microsatellite loci in *Polygonatum cyrtonema* and cross-species amplification. Conserv Genet Resour 2:105-107. <https://doi.org/10.1007/s12686-010-9218-1>

Liu TT, Cheng WJ, Zhou SB, Shao JW, Wu HL, Zhu GP (2010) Eleven polymorphic microsatellite loci in Polygonatum filipes and cross-amplification in other congeneric species. Conserv Genet Resour 2:77-79. <https://doi.org/10.1007/s12686-010-9179-4>

**Clonality among shoot clusters**


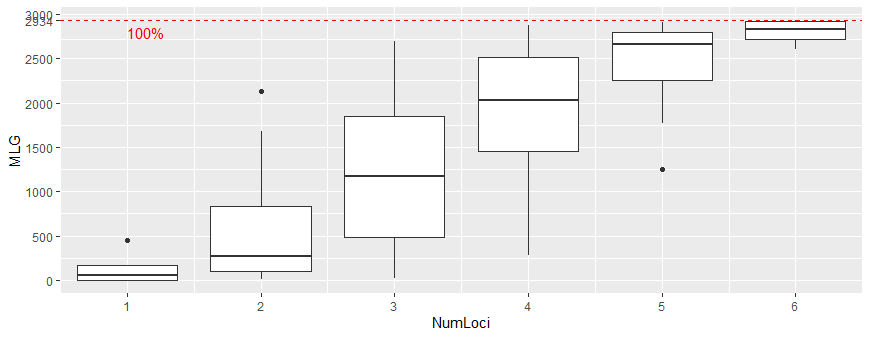


**Figure S4.1:** Multi locus genotype (MLG) accumulation curve based on 200 subsamples of each loci for each number of loci.

**Clonality within shoot clusters**

**Table S4.2:** Detailed overview of the shoots analyzed in the comparisons of shoot clusters of different sizes. Reported are numbers of flowering shoots per cluster, the number of collected samples, the number of samples that were genotyped, and the number of multi locus genotypes identified.

| **Polygonatum patch** | **Flowering shoots** | **Samples collected in the field** | **Genotyped samples** | **MLG** |
| --- | --- | --- | --- | --- |
| F01_P22 | 2 | 2 | 2 | 1 |
| F04_P37 | 2 | 2 | 2 | 1 |
| F04c_P09 | 2 | 2 | 2 | 2 |
| F06b_P37 | 2 | 2 | 2 | 1 |
| F10b_P40 | 2 | 2 | 2 | 2 |
| F04a_P16 | 3 | 3 | 3 | 2 |
| F06_P03 | 3 | 3 | 3 | 1 |
| F48d_P31 | 3 | 3 | 3 | 3 |
| F01a_P39 | 4 | 2 | 2 | 1 |
| F04b_P25 | 4 | 2 | 2 | 2 |
| F06e_P42 | 4 | 2 | 2 | 1 |
| F10a_P22 | 4 | 2 | 2 | 1 |
| F10c_P37 | 4 | 2 | 2 | 1 |
| F04_P05 | 6 | 3 | 3 | 1 |
| F10c_P04 | 6 | 3 | 3 | 2 |
| F48b_P17 | 6 | 2 | 2 | 1 |
| F48j_P24 | 6 | 3 | 3 | 1 |
| F48k_P57 | 6 | 5 | 5 | 5 |
| F07a_P55 | 7 | 2 | 2 | 2 |
| F10_P35 | 7 | 2 | 2 | 1 |
| F48f_P22 | 7 | 2 | 2 | 1 |
| F10c_P45 | 8 | 2 | 2 | 1 |
| F04c_P02 | 9 | 5 | 5 | 3 |
| F10b_P52 | 10 | 2 | 2 | 1 |
| F48_P58 | 10 | 3 | 3 | 2 |
| F48a_P34a | 10 | 2 | 2 | 2 |
| F07b_P17 | 11 | 2 | 2 | 1 |
| F48c_P08 | 11 | 3 | 3 | 2 |
| F01a_P30 | 12 | 5 | 5 | 3 |
| F06a_P43 | 12 | 2 | 2 | 1 |
| F10d_P13 | 12 | 2 | 2 | 1 |
| F48d_P04 | 12 | 3 | 3 | 2 |
| F01a_P53 | 14 | 5 | 5 | 3 |
| F04c_P47a | 15 | 8 | 8 | 4 |
| F08a_P19 | 15 | 2 | 2 | 1 |
| F48e_P30 | 16 | 3 | 3 | 2 |
| F48i_P25 | 16 | 2 | 2 | 2 |
| F10_P51 | 17 | 3 | 3 | 2 |
| F10b_P49b | 17 | 2 | 2 | 1 |
| F04c_P41 | 18 | 3 | 3 | 2 |
| F06e_P48a | 18 | 2 | 2 | 1 |
| F06d_P19 | 20 | 5 | 5 | 1 |
| F48_P42b | 21 | 3 | 3 | 2 |
| F01b_P10 | 22 | 5 | 5 | 1 |
| F10_P43 | 22 | 2 | 2 | 2 |
| F06e_P01 | 24 | 8 | 8 | 1 |
| F10a_P46 | 25 | 3 | 3 | 2 |
| F48c_P43 | 27 | 3 | 3 | 2 |
| F10b_P04 | 33 | 5 | 5 | 2 |
| F48e_P20 | 36 | 5 | 4 | 3 |
| F06b_P39 | 37 | 8 | 8 | 3 |
| F10c_P13 | 46 | 5 | 4 | 1 |
| F04d_P32 | 51 | 8 | 8 | 1 |
| F10c_P44 | 51 | 5 | 5 | 4 |
| F48e_P11 | 67 | 8 | 8 | 7 |
| F48k_P32 | 69 | 10 | 10 | 6 |
| F06e_P38 | 103 | 13 | 11 | 7 |
| F48i_P17 | 119 | 16 | 16 | 14 |
| F07_P03b | 190 | 25 | 25 | 17 |
| Total | 1316 | 249 | 245 | 144 |

**Table S4.3:** The table summarizes how many shoot clusters per size class were included in the assessment of within shoot cluster clonality and the proportion of them that were monoclonal.

|  | Number of included shoot cluster | Monoclonal shoot cluster |
| --- | --- | --- |
| All | 59 | 26 (44%) |
| ≤ 5 flowering shoots | 13 | 8 (62%) |
| 5-20 flowering shoots | 28 | 13 (46%) |
| >20 flowering shoots | 18 | 5 (28%) |

With a higher number of sampled shoots (larger shoot clusters) the percentage of monoclonal shoot clusters is decreasing.

# Supplement 5: Information on quality of paternity analysis

**Table S5.1:** The table shows the reproducibility of the pollen donor-assignment procedure across independent runs. It indicates how many pollen-donor pairs were consistently assigned in n out of five total runs. In cases where different pollen donors were assigned to the same offspring, these were not counted as reproducible unless one donor appeared at least in one run more than the other(s), in which case the dominating was counted as the pollen donor.

| Forest Patch | 1 run | 2 runs | 3 runs | 4 runs | 5 runs | Differing pollen donors | Total | Total of reliable assigned offspring |
| --- | --- | --- | --- | --- | --- | --- | --- | --- |
| F01 |  |  |  | **1** | **8** | **0** | **9** | **9** |
| F04 | **4** | **2** |  | **5** | **18** | **0** | **29** | **25** |
| F06 | **2** | **12** | **3** | **14** | **3** | **0** | **34** | **32** |
| F07 | **17** | **7** | **5** |  | **4** | **3** | **33** | **16** |
| F08 | **3** | **15** | **1** |  | **23** | **0** | **42** | **39** |
| F10 | **1** |  | **3** | **23** | **9** | **1** | **36** | **35** |
| F48 |  |  |  |  | **12** | **0** | **12** | **12** |
| F51 | **1** |  |  | **1** | **5** | **0** | **7** | **6** |
| Total | **28** | **36** | **12** | **44** | **82** | **4** | **202** | **174** |

# Supplement 6: Landscape metrics

**Table S6.1:** Landscape metrics used to quantify the landscape composition in 50 m buffers around pollen receptor plants.

| Area-based metrics | Median, minimum and maximum percentage cover of buffer |
| --- | --- |
| Deciduous Forest | 76 (range: 31.5 - 100) |
| SEMNATGRASS | 0 |
| RAPESEED | 0 |
| MAIZE | 0 (range: 0 - 73) |
| Linear landscape elements | **Median, minimum and maximum length in meters** |
| L_ROAD | 0 (range: 0 - 162.4) |
| L_WATER | 0 (range: 0 - 153.2) |
| L_WOOD | 0 |

**Table S6.2:** Landscape metrics used to quantify the landscape composition in 250 m buffers around pollen receptor plants.

| Area-based metrics | Median, minimum and maximum percentage cover of buffer |
| --- | --- |
| Deciduous Forest | 26.7 (range: 9 - 41.4) |
| SEMNATGRASS | 0 |
| RAPESEED | 0 |
| MAIZE | 7.8 (range: 0 - 71.4) |
| Linear landscape elements | **Median, minimum and maximum length in meters** |
| L_ROAD | 317.3 (range: 0 - 1027.7) |
| L_WATER | 546.5 (range: 0 - 976.2) |
| L_WOOD | 477 (range: 0 - 2100) |

**Table S6.3:** Landscape metrics used to quantify the landscape composition in 1000 m buffers around pollen receptor plants.

| Area-based metrics | Median, minimum and maximum percentage cover of buffer |
| --- | --- |
| Deciduous Forest | 9.5 (range: 5.7 - 16.8) |
| SEMNATGRASS | 0.4 (range: 0 - 1.3) |
| RAPESEED | 0.9 (range: 0 - 21.4) |
| MAIZE | 22.3 (range: 5 - 47.5) |
| Linear landscape elements | **Median, minimum and maximum length in meters** |
| L_ROAD | 5466 (range: 3649 - 9567) |
| L_WATER | 4994.8 (range: 693.5 - 8458.495) |
| L_WOOD | 10139.6 (range: 2275.6 - 19455.1) |


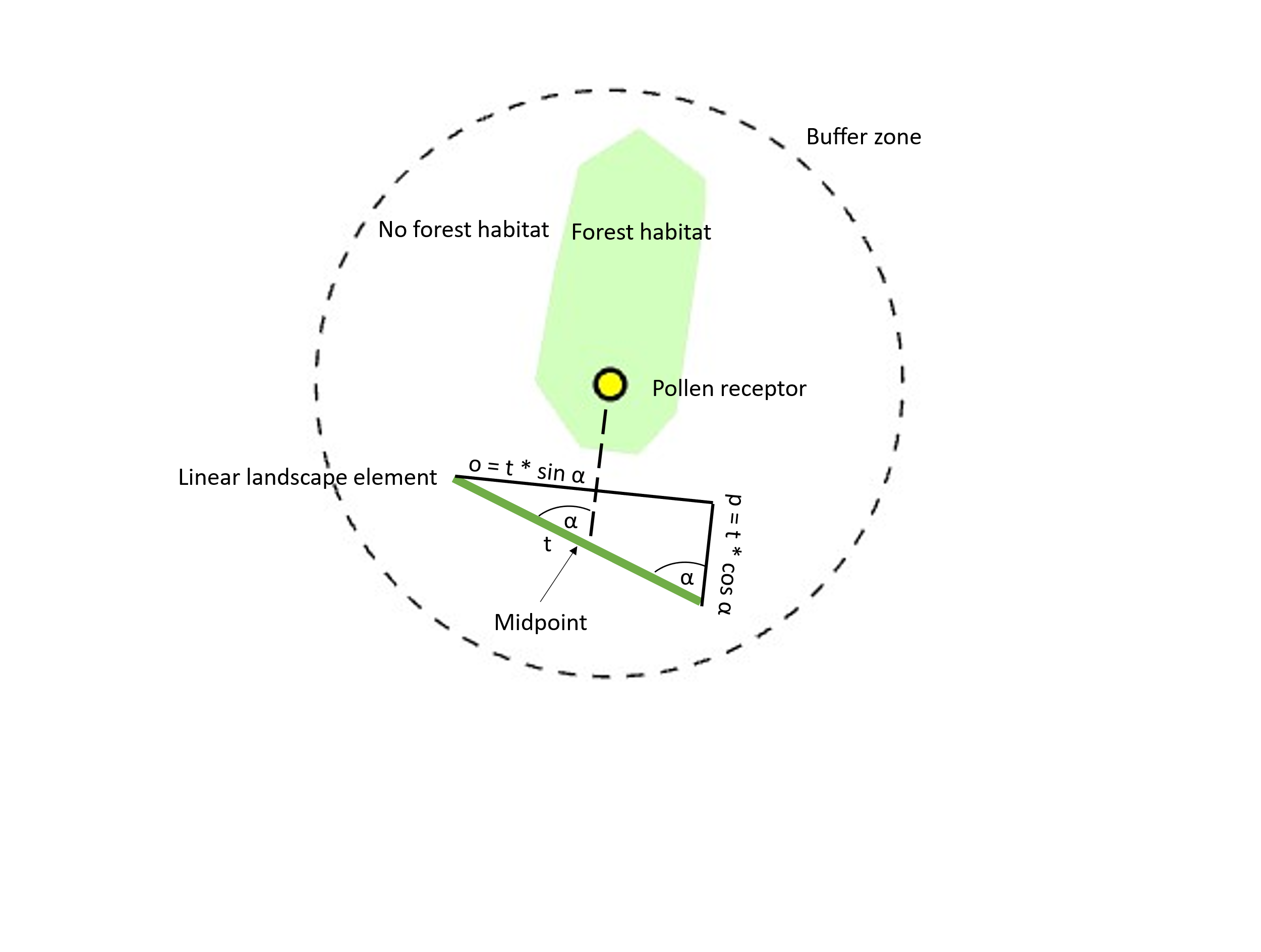


**Figure S6.1:** Schematic visualization of the calculation of parallel (p) and orthogonal (o) length component of each specific linear landscape element.

**
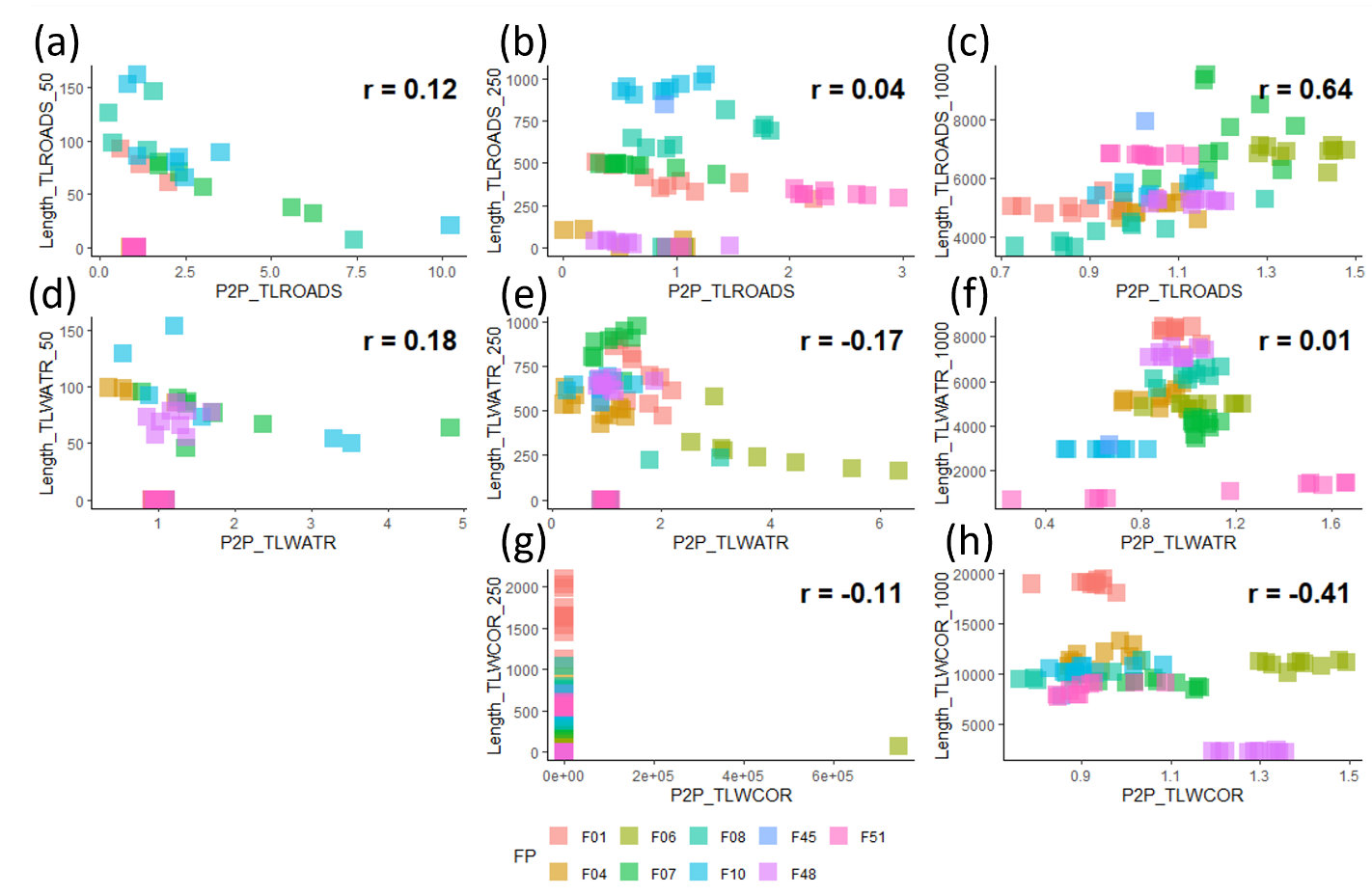
**

**Figure S6.2:** Distribution of the length of linear landscape elements and their orthogonal-parallel ratio. Colors indicate the forest patch from which the pollen receptor originates.

# Supplement 7: Distribution of pollen flow measures

**
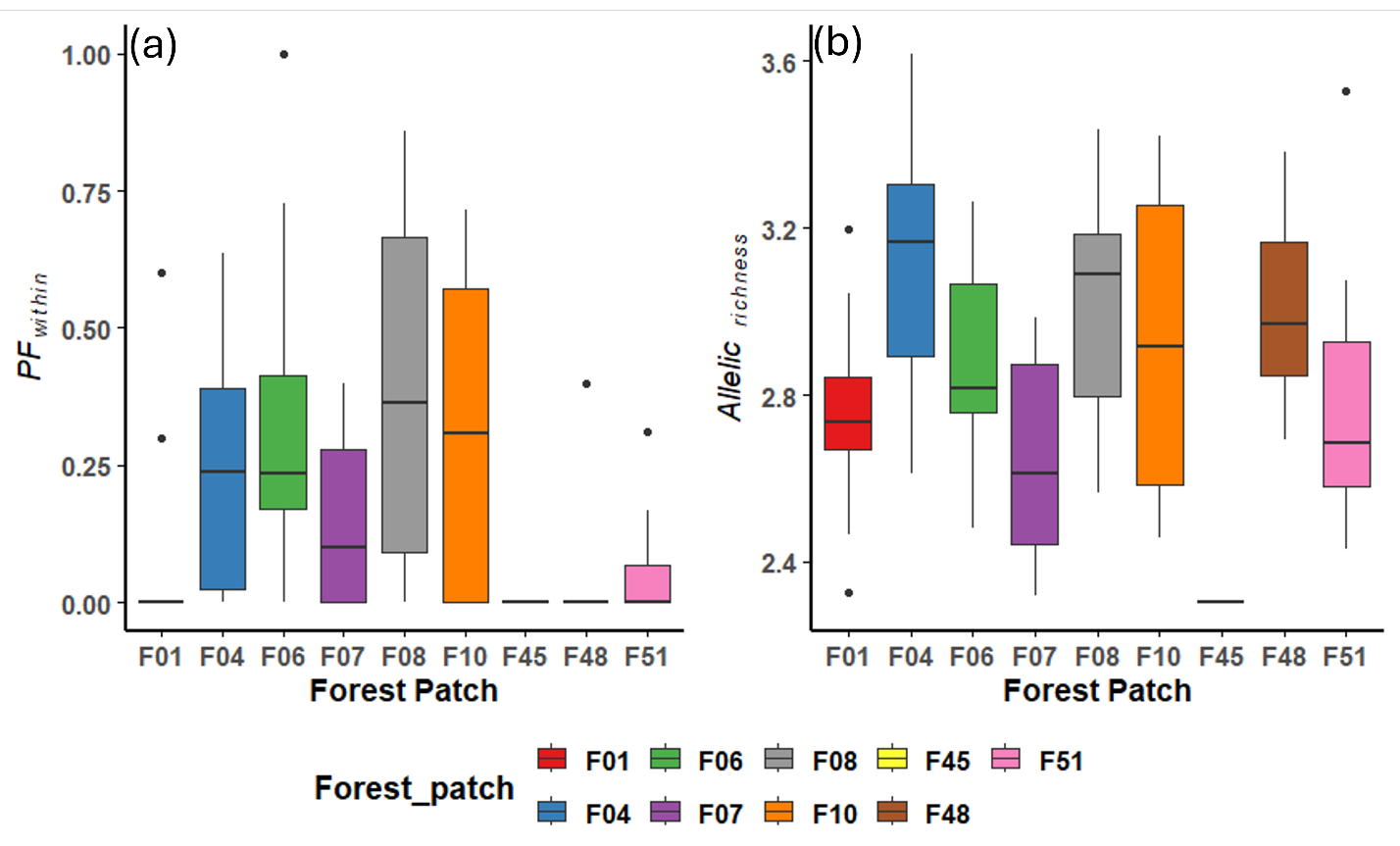
**

**Figure S7.1:** The figure shows the distribution of (a) *PF_within_* and (b) *A_r_* per pollen receptor among forest patches. Colors indicate the forest patch from which the pollen receptor originates.

**
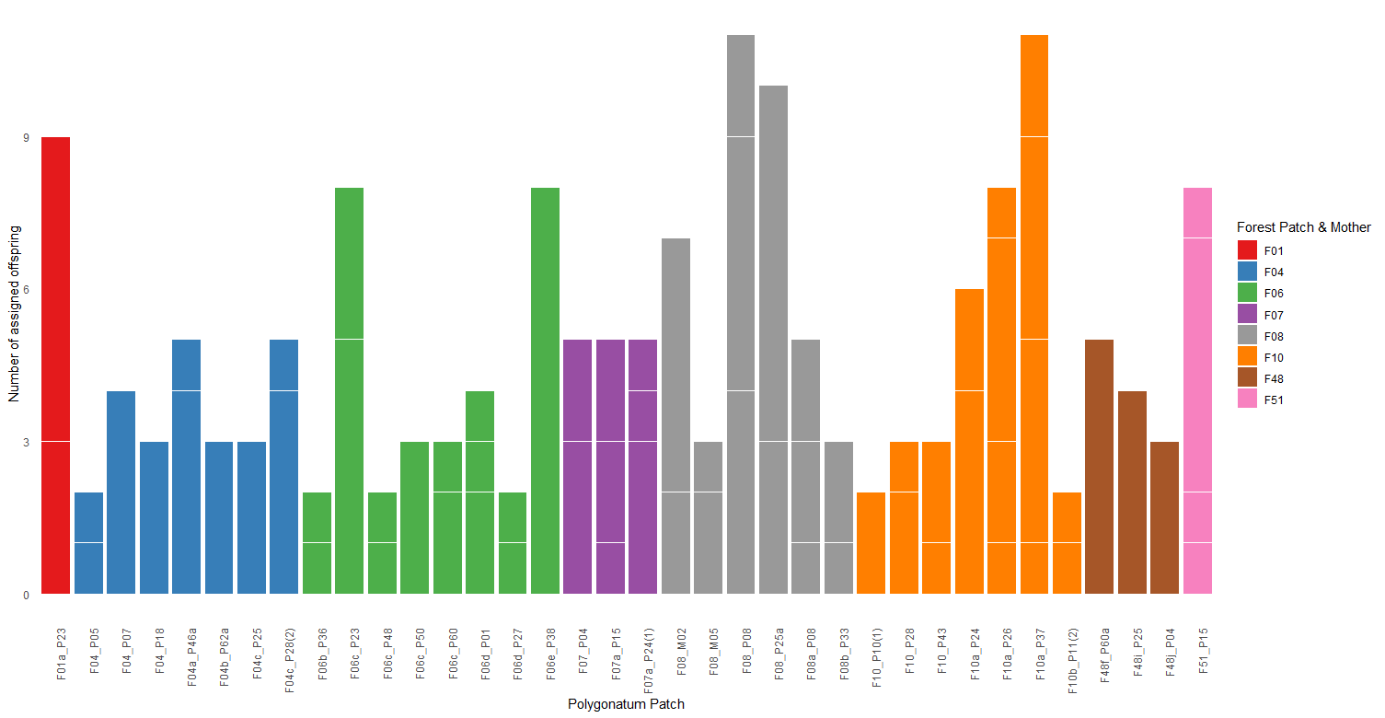
**

**Figure S7.2:** The figure shows the counts of offspring that were assigned to specific pollen donors from the own forest patch. The horizontal lines indicate from how many pollen receptors the offspring originated.

**
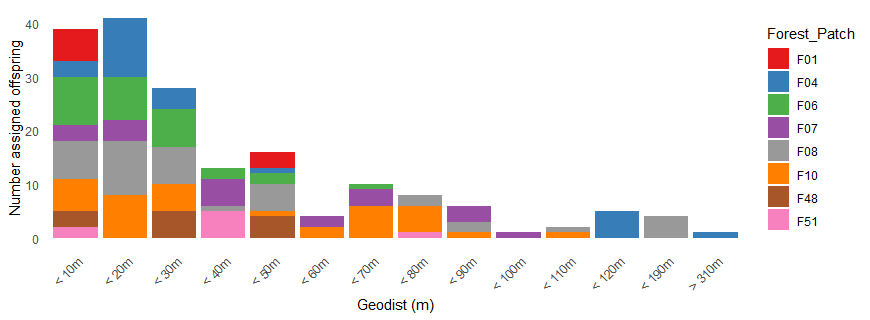
**

**Figure S7.3:** The figure shows the counts of offspring that were assigned to specific pollen donors from the own forest patch at specific distances classes.

**
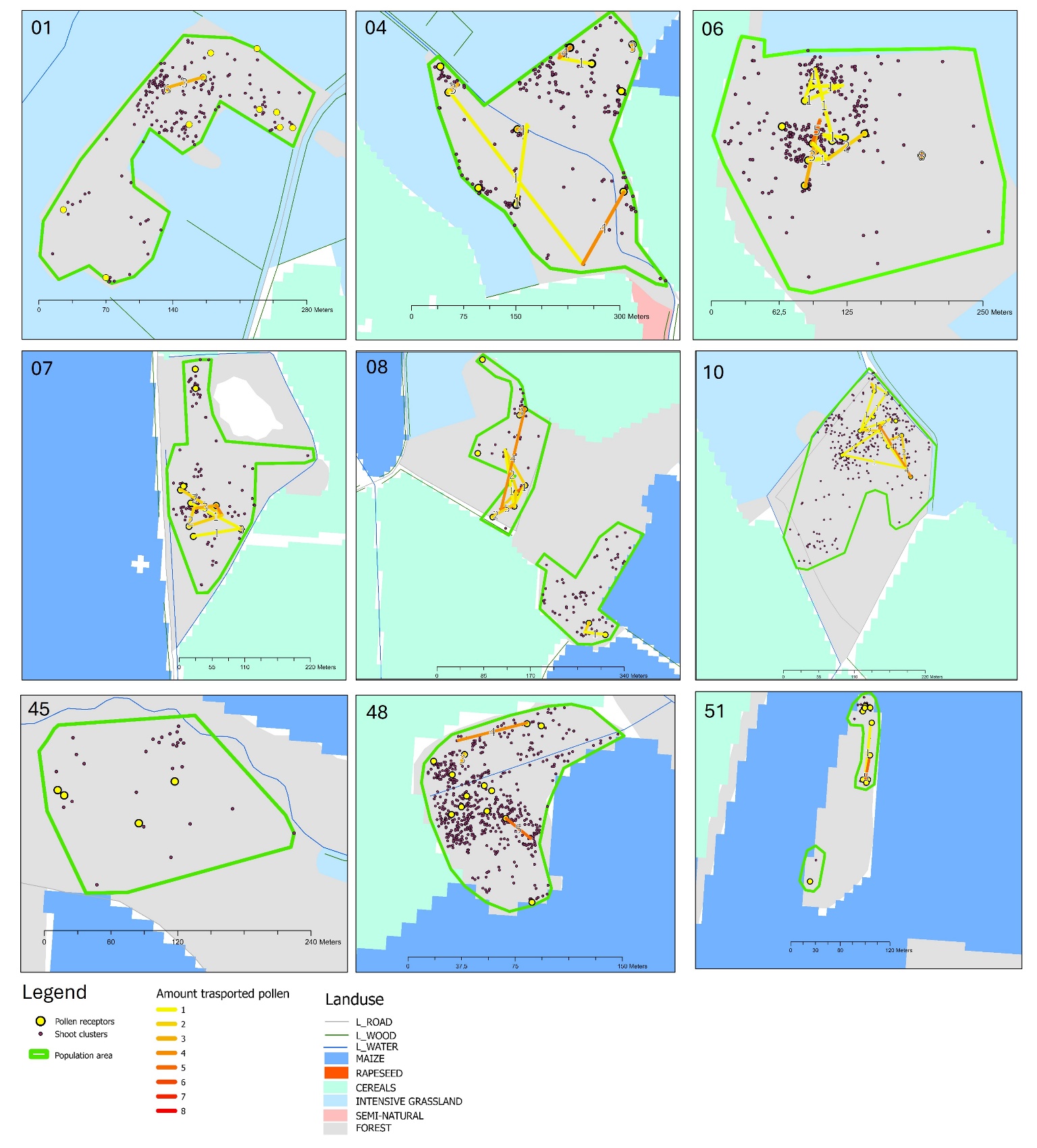
**

**Figure S7.4:** Connection lines between pollen receptors (yellow dots) and identified pollen donors. The numbers on the lines and the color of the lines indicate the number of assigned offspring individuals.

# Supplement 8: Model outcome H2

**Table S8.1:** Model outcome conditional effects for H2.

|  | Estimate | Standard error | *z*-statistic | *p*-value |
| --- | --- | --- | --- | --- |
| (Intercept) | 1.593 | 0.096 | 16.567 | 0.000 |
| Shoot number | -0.048 | 0.059 | -0.811 | 0.418 |
| Distance_to_forest_edge | -0.057 | 0.083 | -0.691 | 0.489 |
| Interaction shoot number and distance_to_forest_edge | 0.065 | 0.038 | 1.704 | 0.088 |

**Table S8.2:** Model outcome zero-inflation effects for H2.

|  | Estimate | Standard error | *z*-statistic | *p*-value |
| --- | --- | --- | --- | --- |
| (Intercept) | 4.118 | 0.287 | 14.344 | 0.000 |
| Shoot number | -0.262 | 0.0938 | -2.796 | 0.005 |
| Distance_to_forest_edge | -0.169 | 0.176 | -0.957 | 0.338 |
| Interaction shoot number and distance_to_forest_edge | -0.045 | 0.085 | -0.532 | 0.594 |

# Supplement 9: Collinearity among included variables

**Table S9.1:** Test for collinearity among all landscape metrics with a *p* < 0.15 in single-metric models for *PF_within_* in the 50 m buffer.

| MAIZE_50 | 1 | -0.20 | -0.07 |
| --- | --- | --- | --- |
| L_ROAD_50 | -0.20 | 1 | 0.23 |
| O:P_ ROAD_50 | -0.07 | 0.23 | 1 |
|  | MAIZE_50 | LROADS_50 | O_P_ LROADS_50 |

**Table S9.2:** Test for collinearity among all landscape metrics with a *p* < 0.15 in single-metric models for *A_r_* in the 50 m buffer.

| MAIZE_50 | 1 | -0.14 |
| --- | --- | --- |
| L_WATER_50 | -0.14 | 1 |
|  | MAIZE_50 | L_WATER_50 |

**Table S9.3:** Test for collinearity among all landscape metrics with a *p* < 0.15 in single-metric models for *PF_within_* in the 250 m buffer.

| MAIZE_250 | 1 | -0.20 | -0.33 |
| --- | --- | --- | --- |
| L_ROAD_250 | -0.20 | 1 | 0.17 |
| N_P_250 | -0.33 | 0.17 | 1 |
|  | MAIZE_250 | L_ROAD_250 | N_P_250 |

**Figure S9.4:** Test for collinearity among all landscape metrics with a *p* < 0.15 in single-metric models for *A_r_* in the 250 m buffer.

| L_WATER_250 | 1 | -0.17 |
| --- | --- | --- |
| O:P_WATER_250 | -0.17 | 1 |
|  | L_WATER_250 | O:P_WATER_250 |

**Table S9.5:** Test for collinearity among all landscape metrics with a *p* < 0.15 in single-metric models for *PF_within_* cond part in the 1000 m buffer. Red colors indicate a Pearson correlation of |r| ≥ 0.7.

| SEMNATGRASS_1000 | 1 | 0.57 | -0.34 | -0.31 | -0.33 | 0.52 | -0.26 | 0.25 | 0.24 |
| --- | --- | --- | --- | --- | --- | --- | --- | --- | --- |
| N_P_1000 | 0.57 | 1 | -0.15 | -0.38 | -0.38 | 0.63 | 0.21 | 0.36 | 0.04 |
| MAIZE_1000 | -0.34 | -0.15 | 1 | -0.54 | 0.60 | -0.56 | 0.43 | 0.39 | 0.32 |
| L_WOOD_1000 | -0.31 | -0.38 | -0.54 | 1 | -0.13 | 0.23 | 0.01 | -0.40 | -0.74 |
| L_ROAD_1000 | -0.33 | -0.38 | 0.60 | -0.13 | 1 | -0.58 | 0.17 | 0.48 | 0.32 |
| L_WATER_1000 | 0.52 | 0.63 | -0.56 | 0.23 | -0.58 | 1 | 0.07 | 0.11 | -0.40 |
| O:P_WATER_1000 | -0.26 | 0.21 | 0.43 | 0.01 | 0.17 | 0.07 | 1 | 0.37 | -0.13 |
| O:P_WOOD_1000 | 0.25 | 0.36 | 0.39 | -0.40 | 0.48 | 0.11 | 0.37 | 1 | 0.28 |
| RAPESEED_1000 | 0.24 | 0.04 | 0.32 | -0.74 | 0.32 | -0.40 | -0.13 | 0.28 | 1 |
|  | SEMNATGRASS_1000 | N_P_1000 | MAIZE_1000 | L_WOOD_1000 | L_ROAD_1000 | L_WATER_1000 | O:P_WATER_1000 | O:P_WOOD_1000 | RAPESEED_1000 |

**Table S9.6:** Test for collinearity among all landscape metrics with a *p* < 0.15 in single-metric models for *PF_within_* zero-inflation part in the 1000 m buffer. Red colors indicate a Pearson correlation of |r| ≥ 0.7.

| L_WATER_1000 | 1 | 0.07 | -0.58 | -0.40 | 0.23 | 0.11 |
| --- | --- | --- | --- | --- | --- | --- |
| O:P_WATER_1000 | 0.07 | 1 | 0.17 | -0.13 | 0.01 | 0.37 |
| L_ROAD_1000 | -0.58 | 0.17 | 1 | 0.32 | -0.13 | 0.48 |
| RAPESEED_1000 | -0.40 | -0.13 | 0.32 | 1 | -0.74 | 0.28 |
| L_WOOD_1000 | 0.23 | 0.01 | -0.13 | -0.74 | 1 | -0.40 |
| O:P_WOOD_1000 | 0.11 | 0.37 | 0.48 | 0.28 | -0.40 | 1 |
|  | L_WATER_1000 | O:P_WATER_1000 | L_ROAD_1000 | RAPESEED_1000 | L_WOOD_1000 | O:P_WOOD_1000 |

**Table S9.7:** Test for collinearity among all landscape metrics with a *p* < 0.15 in single-metric models for *A_r_* in the 1000 m buffer.

| MAIZE_1000 | 1 | -0.15 | -0.34 | 0.60 |
| --- | --- | --- | --- | --- |
| N_P_1000 | -0.15 | 1 | 0.57 | -0.38 |
| SEMNATGRASS_1000 | -0.34 | 0.57 | 1 | -0.33 |
| L_ROAD_1000 | 0.60 | -0.38 | -0.33 | 1 |
|  | MAIZE_1000 | N_P_1000 | SEMNATGRASS_1000 | L_ROAD_1000 |

# Supplement 10: Outcome of Model averaging for H3 and H4

**Table S10.1:** Overview over model averaging.

|  | Buffer size | Variables from single metric models | Number of best models |
| --- | --- | --- | --- |
| *PF_within_* | 50 | Cond:   - MAIZE - L_ROAD - O:P_ROAD   ZI:  --- | 1 |
| *A_r_* | 50 | - MAIZE - L_WATER | 2 |
| *PF_within_* | 250 | Cond:   - MAIZE - L_ROAD   ZI:   - N_P | 1 |
| *A_r_* | 250 | - L_WATER - O:P_WATER | 1 |
| *PF_within_* | 1000 | Cond:   - MAIZE - SEMNATGRASS - RAPESEED - L_ROAD - L_WATER   Zi:   - RAPSEED - L_ROAD - L_WATER - L_WOOD - O:P_WOOD - O:P_ WATER | 14 |
| *A_r_* | 1000 | - MAIZE - SEMNATGRASS - L_ROADS - N_P | 5 |

**Table S10.2:** The table summarizes the outcome of the single best model for *PF_within_* at the 50 m buffer.

|  | Estimate | Standard error | *z*-statistic | *p*-value |
| --- | --- | --- | --- | --- |
| (Intercept) | 0.15 | 0.374 | 0.4 | 0.689 |
| MAIZE_50 | 0.384 | 0.442 | 0.869 | 0.385 |
| I(MAIZE_50^2) | -0.910 | 0.355 | -2.562 | 0.010 |

**Table S10.3:** The table summarizes the outcome of the model averaging procedure for *A_r_* at the 50 m buffer.

|  | Estimate | Standard error | *z*-statistic | *p*-value |
| --- | --- | --- | --- | --- |
| (Intercept) | 2.861 | 0.065 | 42.963 | 0.000 |
| L_WATER_50 | 0.129 | 0.039 | 3.253 | 0.001 |
| MAIZE_50 | -0.077 | 0.045 | 1.689 | 0.091 |

**Table S10.4:** The table summarizes the outcome of the model averaging procedure for *PF_within_* at the 250 m buffer.

|  | Estimate | Standard error | *z*-statistic | *p*-value |
| --- | --- | --- | --- | --- |
| Cond: (Intercept) | -0.196 | 0.170 | -1.150 | 0.250 |
| Cond: L_ROAD_250 | 0.217 | 0.101 | 2.147 | 0.032 |
| Cond: MAIZE_250 | -0.513 | 0.132 | -3.891 | 0.0001 |
| Cond: I(MAIZE_250^2) | -0.618 | 0.159 | -3.89 | 0.0001 |
| ZI: (Intercept) | -0.206 | 0.308 | -0.668 | 0.504 |
| ZI: N_P_250 | 1.189 | 0.425 | 2.794 | 0.005 |

**Table S10.5:** The table summarizes the outcome of the model averaging procedure for *A_r_* at the 250 m buffer.

| column name | Estimate | Standard error | *t*-statistic | *p*-value |
| --- | --- | --- | --- | --- |
| (Intercept) | 2.840 | 0.050 | 56.749 | 0.000 |
| L_WATER_250 | -0.058 | 0.045 | -1.268 | 0.209 |
| O:P_WATER_250 | -0.135 | 0.038 | -3.596 | 0.0006 |
| Interaction L_WATER_250 and O:P_WATER_250 | -0.152 | 0.048 | -3.141 | 0.003 |

**Table S10.6:** The table summarizes the outcome of the model averaging procedure for *PF_within_* at the 1000 m buffer.

|  | Estimate | Standard error | *z*-statistic | *p*-value |
| --- | --- | --- | --- | --- |
| cond((Int)) | -0.241 | 0.303 | 0.784 | 0.433 |
| cond(MAIZE_1000) | -0.922 | 0.385 | 2.364 | 0.018 |
| cond(I(MAIZE_1000^2)) | -0.701 | 0.309 | 2.227 | 0.026 |
| cond(RAPESEED_1000) | -0.444 | 0.244 | 1.795 | 0.073 |
| cond(I(RAPESEED_1000^2)) | 0.224 | 0.242 | 0.909 | 0.364 |
| cond(SEMNATGRASS_1000) | -0.128 | 0.239 | 0.524 | 0.600 |
| cond(I(SEMNATGRASS _1000^2)) | -0.351 | 0.124 | 2.77 | 0.006 |
| cond(L_WATER_1000) | -0.383 | 0.378 | 0.995 | 0.32 |
| zi((Int)) | -1.039 | 0.598 | 1.717 | 0.086 |
| zi(RAPESEED_1000) | 0.694 | 0.440 | 1.554 | 0.120 |
| zi(L_WATER_1000) | 1.987 | 0.706 | 2.773 | 0.006 |
| zi(O:P_WATER_1000) | -1.916 | 1.066 | 1.769 | 0.077 |
| zi(Interaction L_WATER_1000 and O:P_WATER_1000) | -0.183 | 0.599 | 0.300 | 0.764 |
| zi(L_ROAD_1000) | 0.761 | 0.492 | 1.52 | 0.129 |
| zi(L_WOOD_1000) | 0.021 | 0.366 | 0.057 | 0.955 |
| zi(O:P_WOOD_1000) | -0.435 | 0.439 | 0.977 | 0.328 |
| zi(Interaction L_ WOOD _1000 and O:P_ WOOD _1000) | -1.446 | 0.430 | 3.308 | 0.001 |

**Table S10.7:** The table summarizes the outcome of the model averaging procedure for *A_r_* at the 1000 m buffer.

|  | Estimate | Standard error | *z*-statistic | *p*-value |
| --- | --- | --- | --- | --- |
| (Intercept) | 2.961 | 0.045 | 64.964 | 0.000 |
| L_ROADS_1000 | -0.078 | 0.042 | 1.816 | 0.069 |
| MAIZE_1000 | -0.027 | 0.046 | 0.578 | 0.563 |
| I(MAIZE_1000^2) | -0.086 | 0.029 | 2.884 | 0.004 |
| SEMNATGRASS_1000 | 0.032 | 0.036 | 0.851 | 0.395 |
