# Supplementary material for: Greater pollen-mediated gene flow among than within populations of a bumblebee-pollinated forest herb despite habitat fragmentation
Source: Mov Ecol. 2026 May 30;14:36. doi: 10.1186/s40462-026-00664-8 (PMC13224658; doi:10.1186/s40462-026-00664-8)
Supplement: Supplementary file 2 — Supplementary Material 2 [file 40462_2026_664_MOESM2_ESM.docx]

**Supplement: Greater pollen-mediated gene flow among than within populations of a bumblebee-pollinated forest herb despite habitat fragmentation**

[Supplement 1: Shoot clusters of *Polygonatum multiflorum* and their bumblebee pollinators 2](#_Toc226905001)

[Supplement 2: Overview over included samples 4](#_Toc226905002)

[Supplement 3: Stratification and germination 14](#_Toc226905003)

[Supplement 4: Genotyping, quality and assessment of clonality 15](#_Toc226905004)

[Supplement 5: Information on quality of paternity analysis 20](#_Toc226905005)

[Supplement 6: Landscape metrics 21](#_Toc226905006)

[Supplement 7: Distribution of pollen flow measures 23](#_Toc226905007)

[Supplement 8: Model outcome H2 34](#_Toc226905008)

[Supplement 9: Collinearity among included variables 36](#_Toc226905009)

[Supplement 10: Outcome of Model averaging for H3 and H4 39](#_Toc226905010)

[Supplement 11: Models used for figures in the main text 43](#_Toc226905011)

# Supplement 1: Shoot clusters of *Polygonatum multiflorum* and their bumblebee pollinators

| **A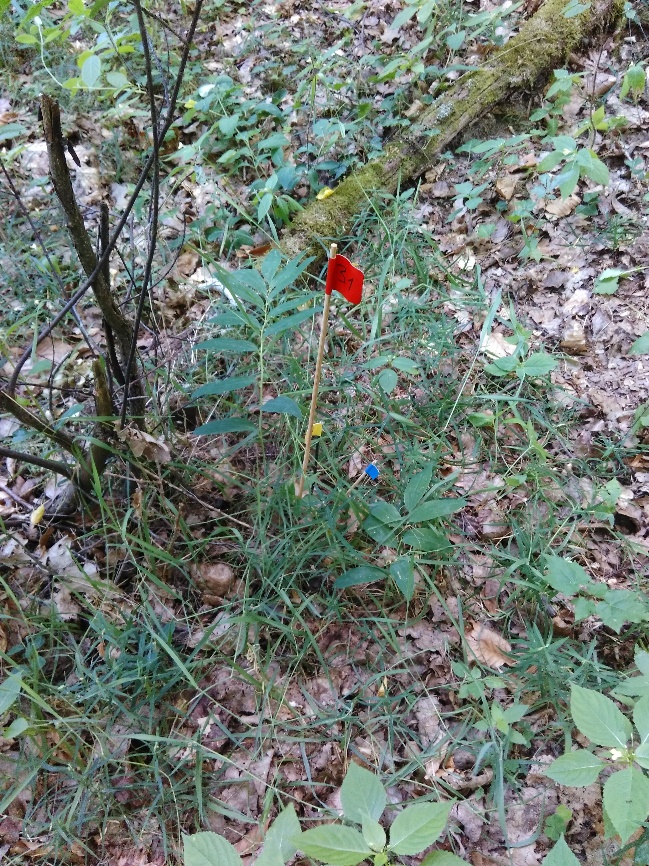** | **B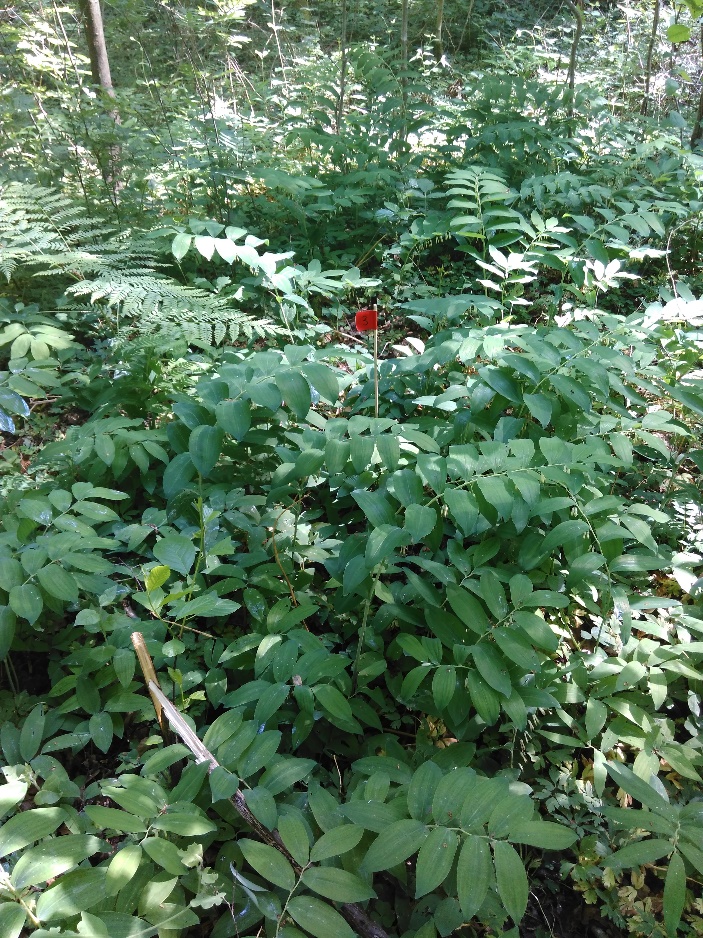** |
| --- | --- |
| **C** | **D** |
| **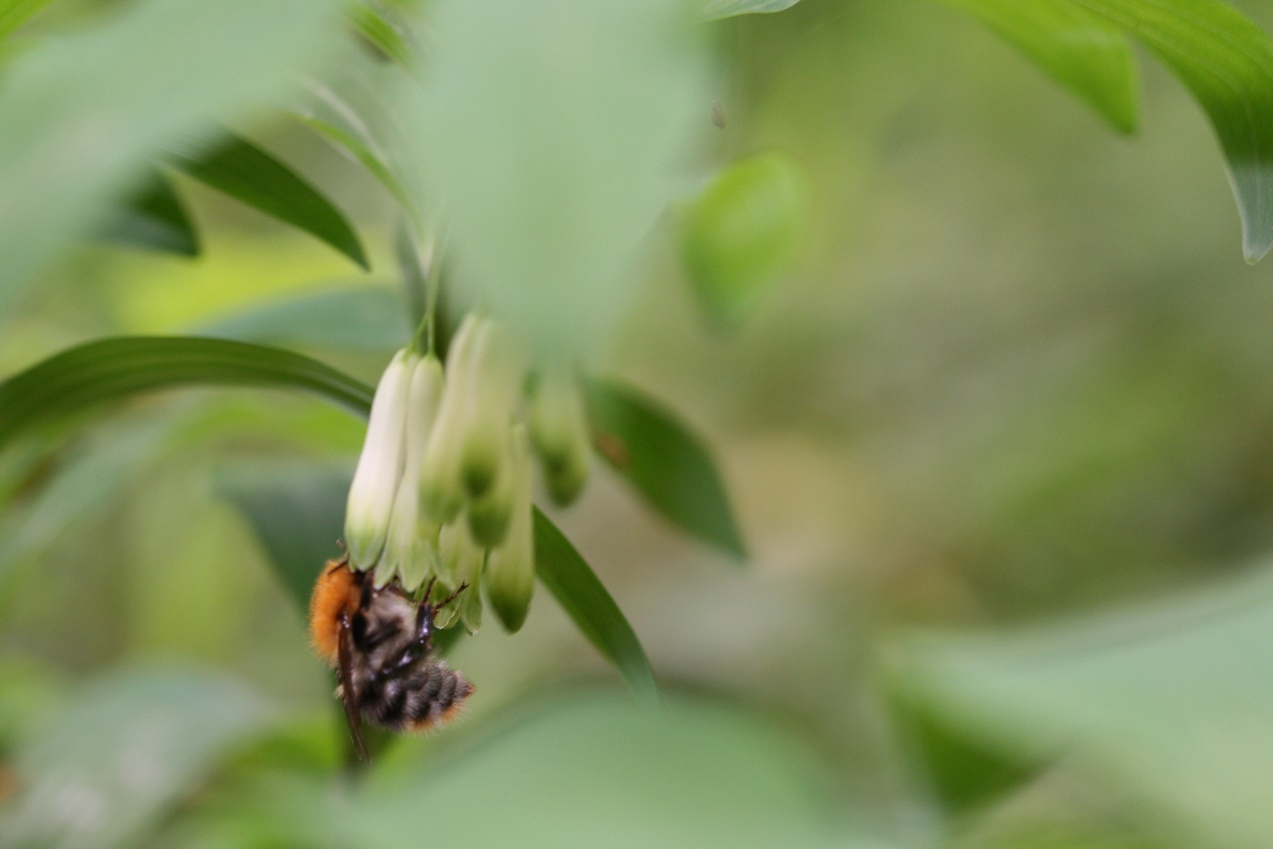** | **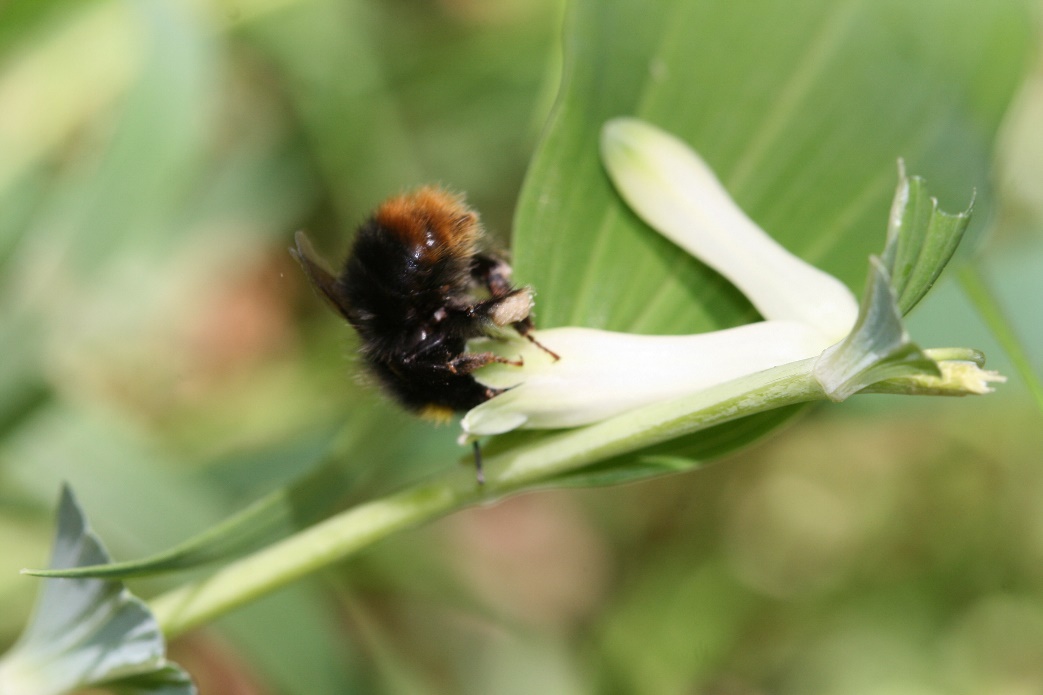** |

**Figure S1:** Pictures A and B: Shoot clusters of *P. multiflorum* with only a few flowering shoots (A) and multiple shoots (B); pictures C and D: Flower visits of *Bombus pascuorum* (C) and *Bombus pratorum* (D).

**Table S1:** During 26 hours of flower observations, we recorded between one and four different bumblebee species per forest patch visiting *P. multiflorum*, out of a total of five species. In addition, we observed other wild bee visitors from the genus *Anthophora*. The table shows the total observation time in 2018 and 2019, as well as the number of individuals per species per forest patch. Details of the observation procedure are provided in Naaf et *al*. (2021), Supplement S1.

| Forest patch ID | Time observed  [hours:min] | *B. pascuorum* | *B. pratorum* | *B. hortorum* | *B. lapidarius* | *B. hypnorum* | *Bombus species* | *Anthophora spp* |
| --- | --- | --- | --- | --- | --- | --- | --- | --- |
| F01 | 4:30 | 7 | 6 | 0 | 1 | 0 | 3 | 0 |
| F04 | 2:00 | 4 | 0 | 0 | 0 | 0 | 1 | 0 |
| F06 | 1:30 | 3 | 2 | 0 | 0 | 0 | 2 | 0 |
| F07 | 4:00 | 16 | 10 | 0 | 0 | 0 | 2 | 0 |
| F08 | 3:00 | 7 | 4 | 0 | 0 | 0 | 2 | 0 |
| F10 | 3:30 | 5 | 11 | 0 | 0 | 1 | 3 | 2 |
| F45 | 1:30 | 1 | 0 | 0 | 0 | 0 | 1 | 0 |
| F48 | 3:00 | 6 | 13 | 2 | 1 | 0 | 4 | 2 |
| F51 | 3:00 | 4 | 0 | 0 | 0 | 0 | 1 | 0 |

**Reference**

Naaf, T., Feigs, J. T., Huang, S., Brunet, J., Cousins, S. A. O., Decocq, G. Sensitivity to habitat fragmentation across European landscapes in three temperate forest herbs. *Landscape Ecology*. 2021; *36*(10), 2831-2848. <https://doi.org/10.1007/s10980-021-01292-w>

# Supplement 2: Overview over included samples


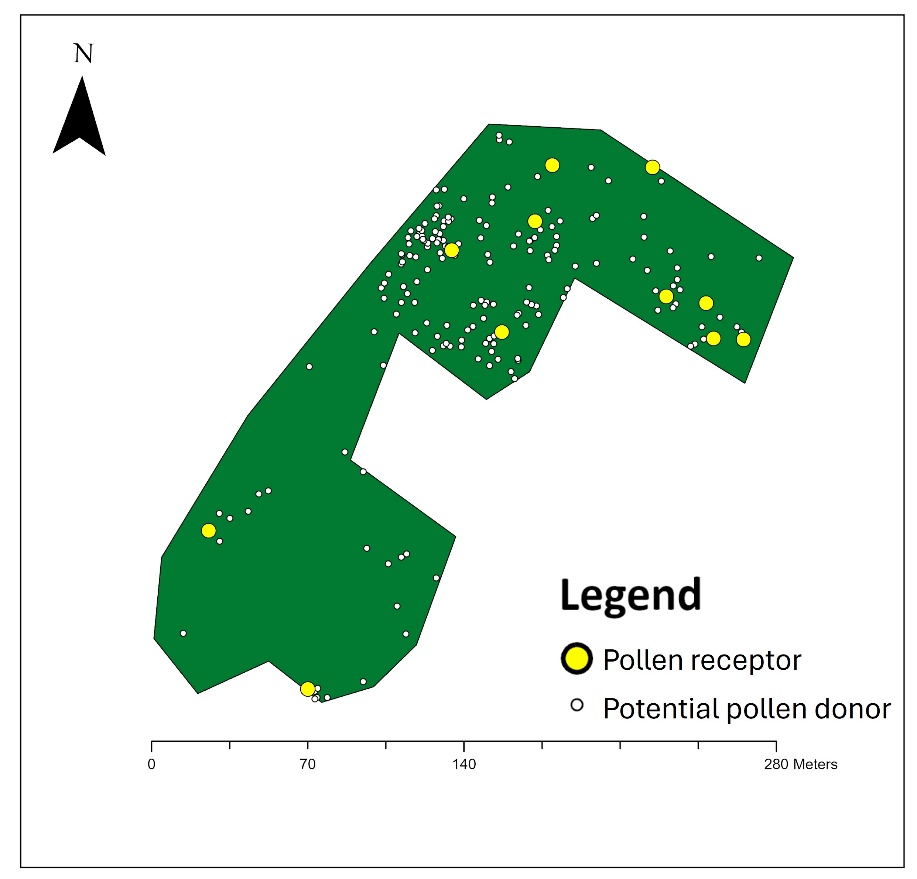


**Figure S2a:** This figure shows the sampling in forest patch F01. Yellow dots represent the plants selected as pollen receptors, while grey dots indicate all potential pollen donors.


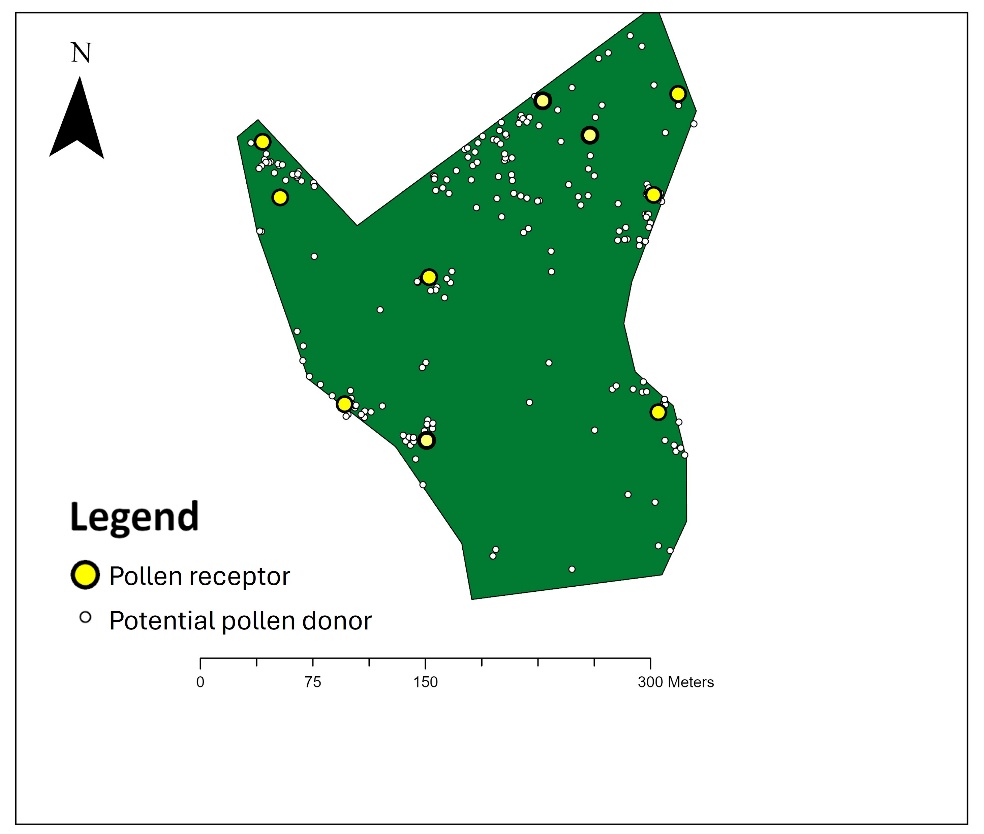


**Figure S2b:** This figure shows the sampling in forest patch F04. Yellow dots represent the plants selected as pollen receptors, while grey dots indicate all potential pollen donors.

**
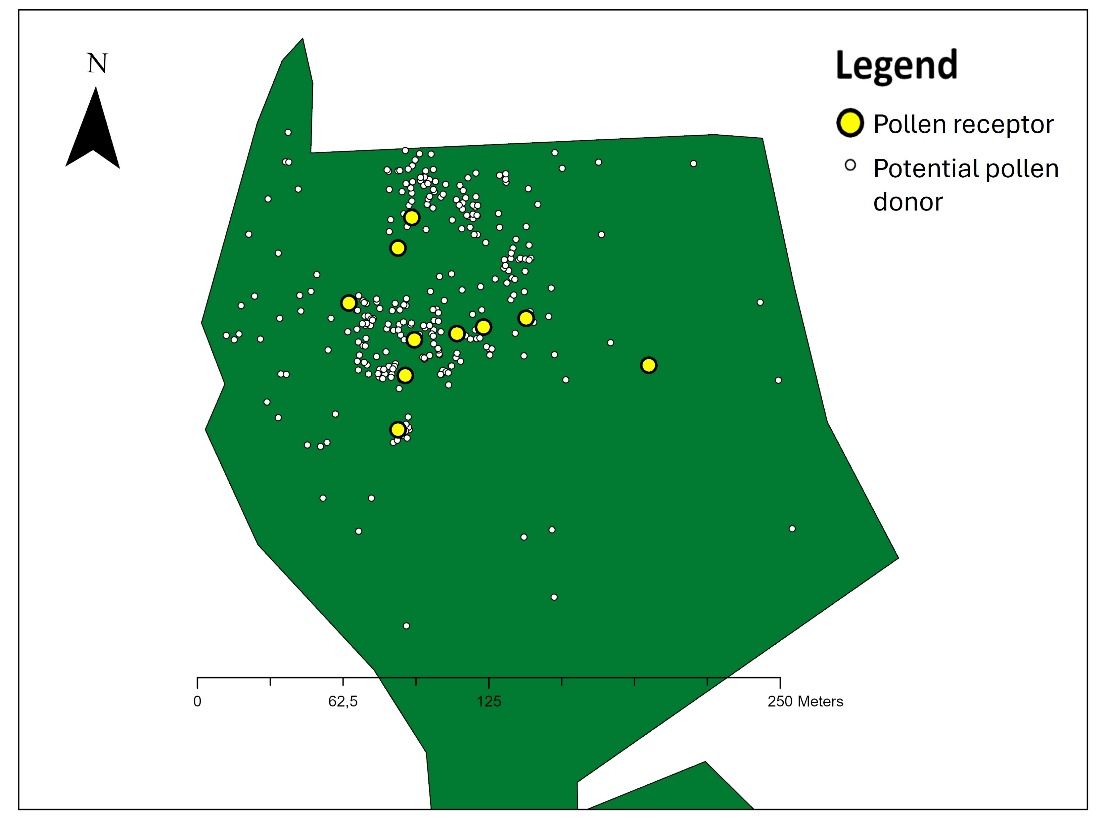
**

**Figure S2c:** This figure shows the sampling in forest patch F06. Yellow dots represent the plants selected as pollen receptors, while grey dots indicate all potential pollen donors.


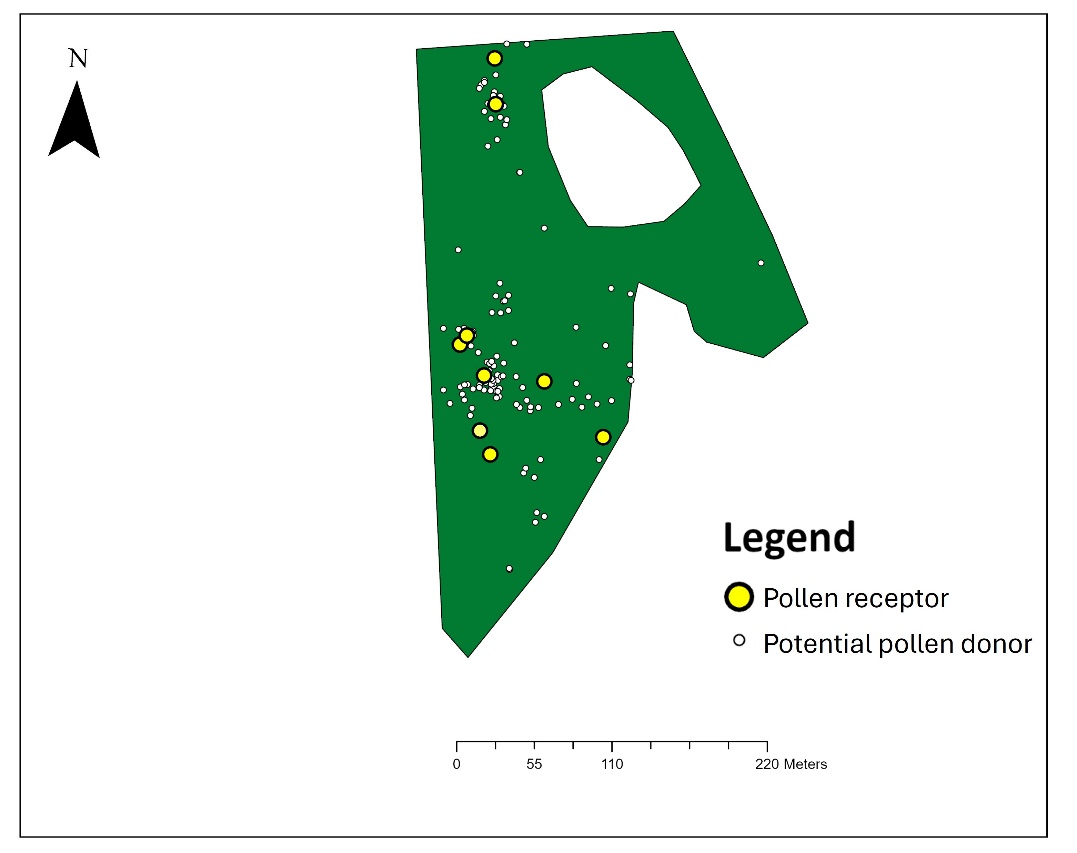


**Figure S2d:** This figure shows the sampling in forest patch F07. Yellow dots represent the plants selected as pollen receptors, while grey dots indicate all potential pollen donors.

**
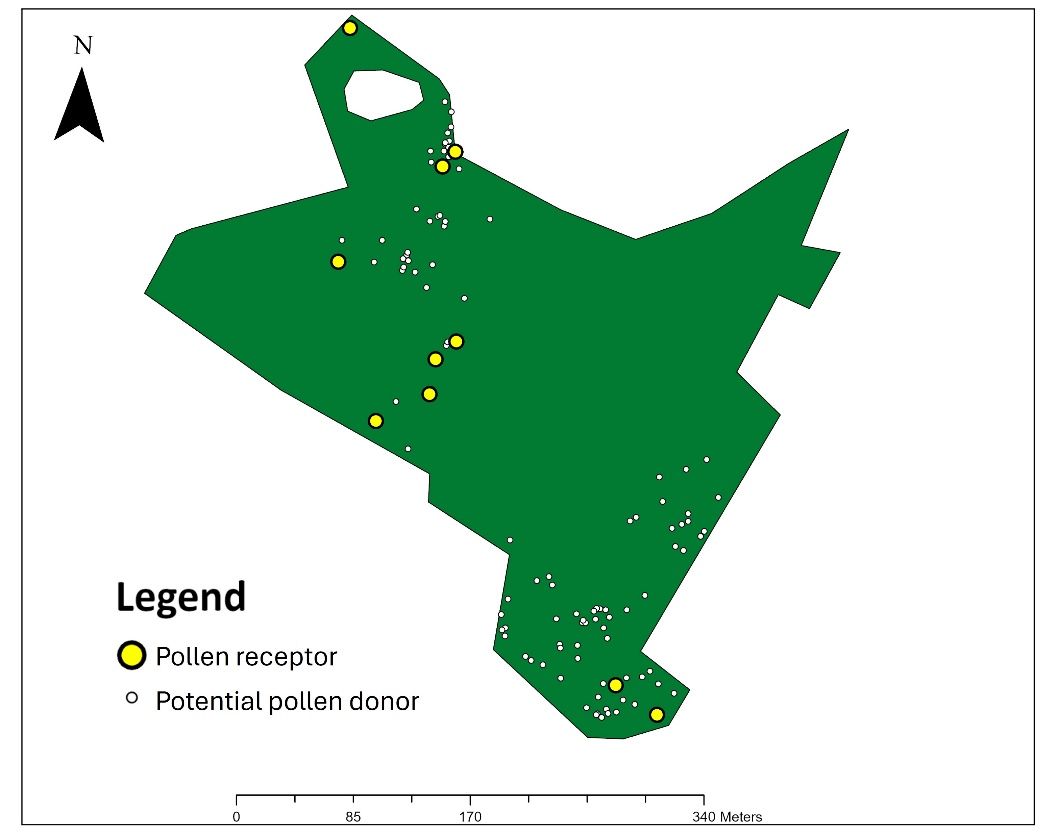
**

**Figure S2e:** This figure shows the sampling in forest patch F08. Yellow dots represent the plants selected as pollen receptors, while grey dots indicate all potential pollen donors.


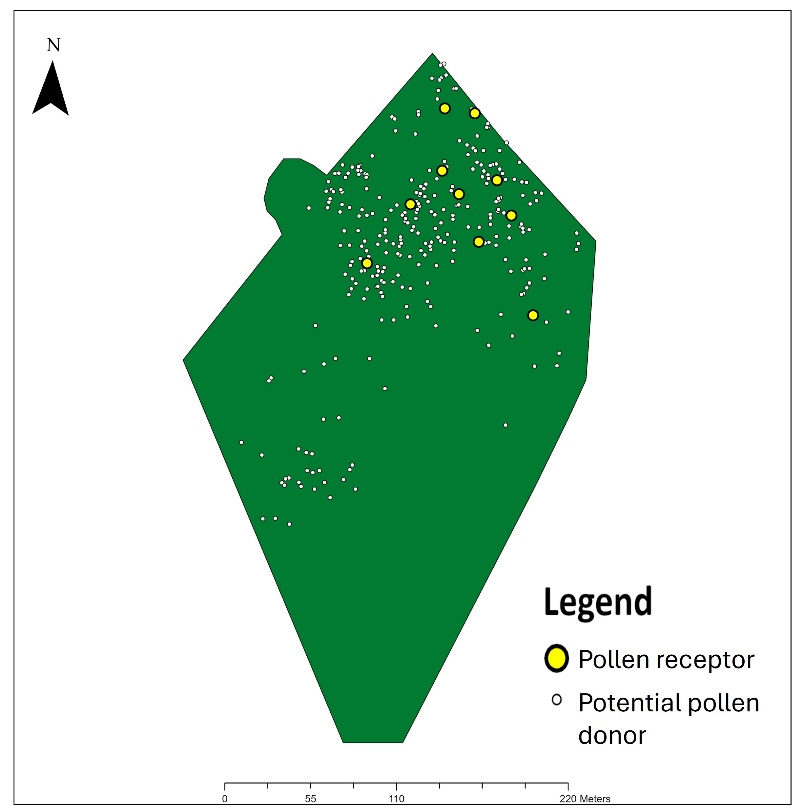


**Figure S2f:** This figure shows the sampling in forest patch F10. Yellow dots represent the plants selected as pollen receptors, while grey dots indicate all potential pollen donors.

**
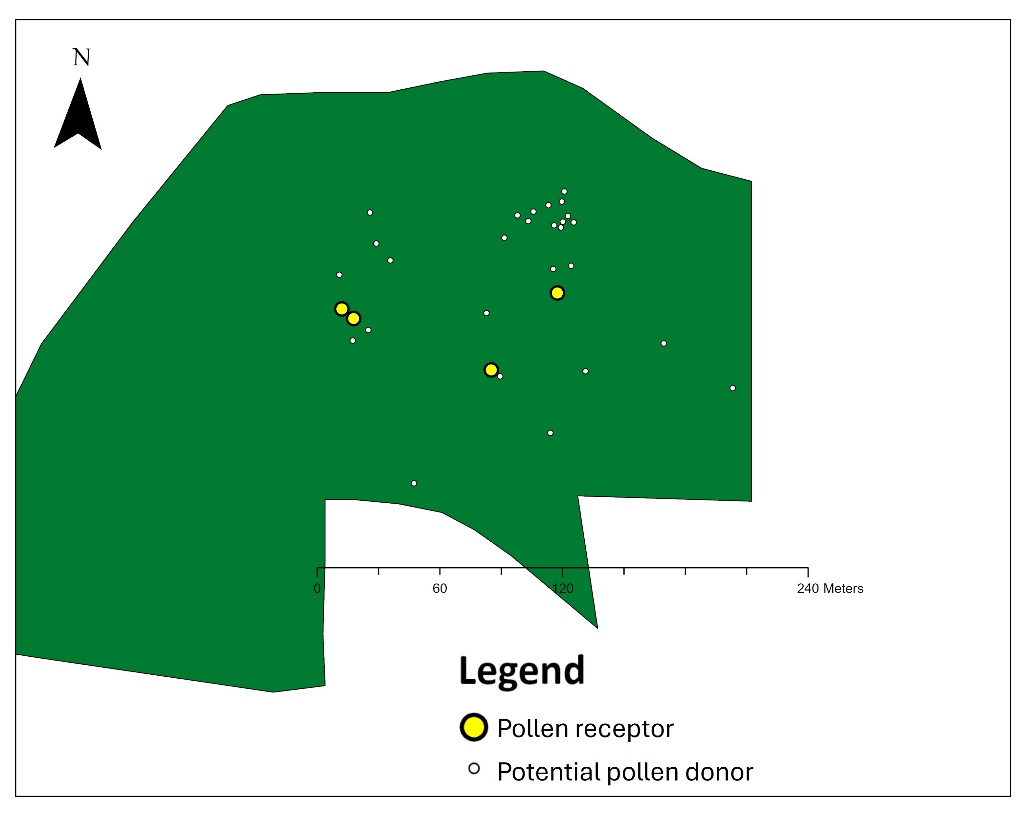
**

**Figure S2g:** This figure shows the sampling in forest patch F45. Yellow dots represent the plants selected as pollen receptors, while grey dots indicate all potential pollen donors.


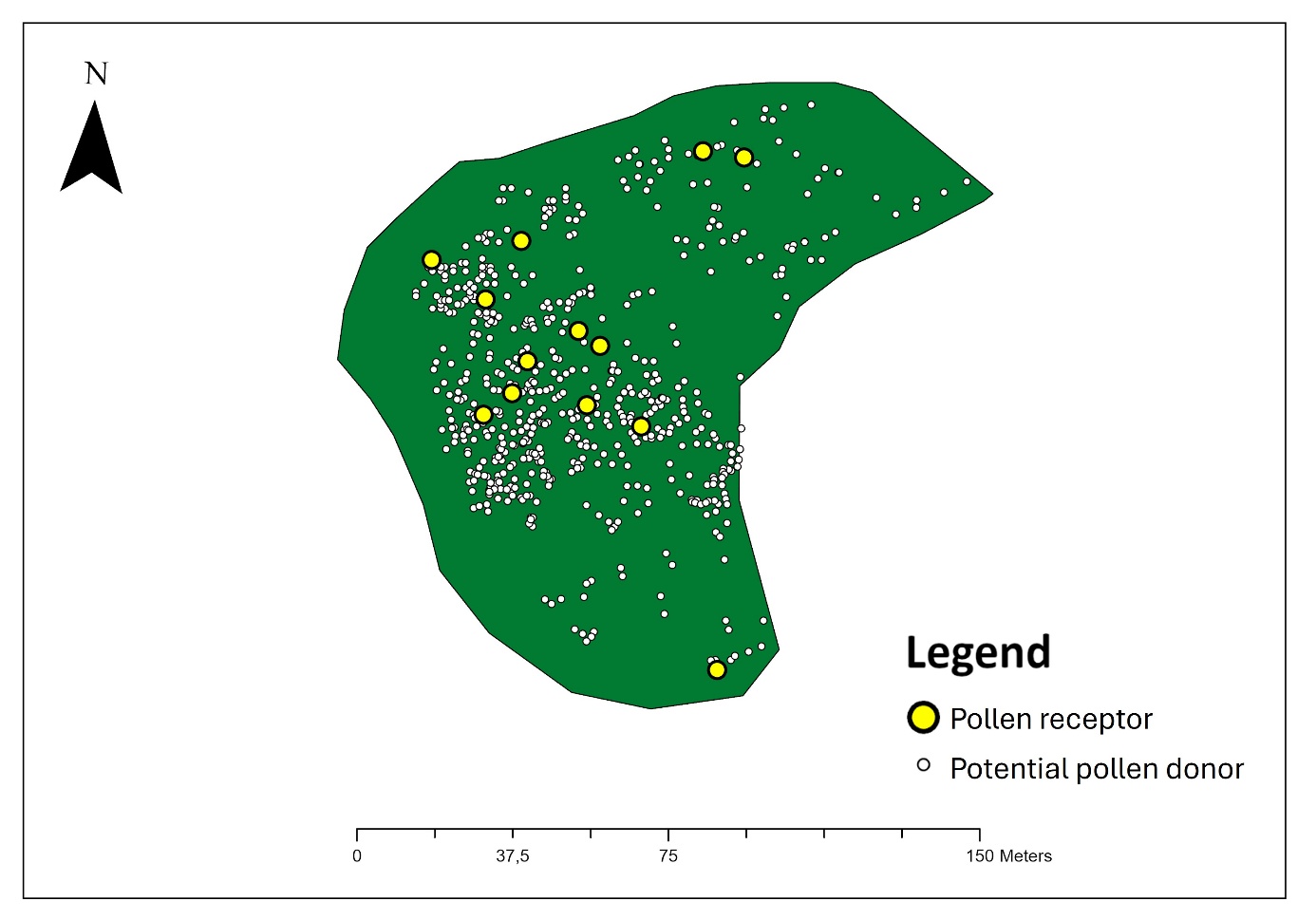


**Figure S2h:** This figure shows the sampling in forest patch F48. Yellow dots represent the plants selected as pollen receptors, while grey dots indicate all potential pollen donors.


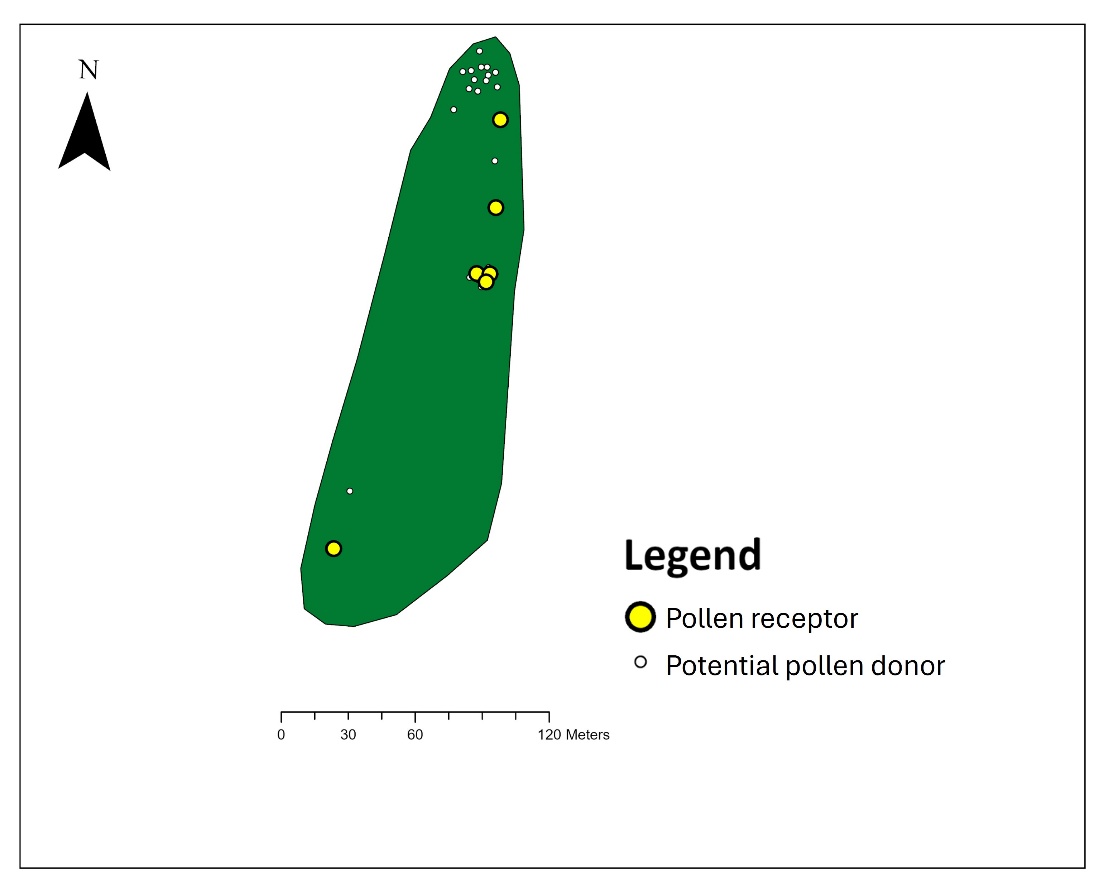


**Figure S2i:** This figure shows the sampling in forest patch F10. Yellow dots represent the plants selected as pollen receptors, while grey dots indicate all potential pollen donors.

**Table S2.1 Column descriptions:** explaining the content of each column of “Table S2.1.xlsx”, provides the exact number of samples involved at each step of the analysis. For clarity, we divided the study process into four steps, organized as follows: **Step 1:** Sampling and germination; **Step 2:** Quality and clonality checks, **Step 3:** Paternity analysis, **Step 4:** Samples used for testing H1, H3 and H4.

| Column name | Description |
| --- | --- |
| Step 1: Sampling | This step is based on the dataset Table S2.3 Al_pollen_donors.xlsx |
| n sampled shoot cluster | The total number of shoots within each forest patch. All sampled in 2018. For each cluster, the number of shoots was documented. See Figure S2 for the spatial distribution of the shoot clusters. |
| n shoot cluster = 1 shoot | Number of all single shoots from each forest patch that were sampled in spring 2018. |
| n shoot cluster 2-9 shoots | Number of all shoot clusters with two to nine shoots from each forest patch sampled in spring 2018. |
| n shoot cluster 10-19 shoots | Number of all shoot clusters with ten to 19 shoots from each forest patch sampled in spring 2018. |
| n shoot cluster >= 20 shoots | Number of all shoot clusters with more than 19 shoots sampled from each forest patch sampled in spring 2018. |
| Proportion of shoot cluster = 1 shoot | Proportion of single shoots among the n sampled shoot clusters per forest patch. |
| Proportion of shoot cluster 2-9 shoots | Proportion of clusters with two to nine shoots among the n sampled shoot clusters per forest patch. |
| Proportion of shoot cluster 9-19 shoots | Proportion of shoot clusters with ten to 19 shoots among the n sampled shoot clusters per forest patch. |
| Proportion of shoot cluster >= 20 shoots | Proportion of shoot clusters with more than 19 shoots among the n sampled shoot clusters per forest patch. |
| Step 2 Quality and clonality checks | This step is based on the dataset Table S4.2 Allele_table.xlsx |
| n successfully genotyped samples | Number of leaf and rhizome samples successfully genotyped after DNA isolation. |
| n successfully genotyped leaf samples as potential pollen donors | Number of leaf samples, successfully genotyped after DNA isolation, that were considered as potential pollen donors. |
| n successfully genotyped leaf samples for within shoot clusters | Number of leaf samples, successfully genotyped after DNA isolation, that were used for the clonality test within the shoot clusters. |
| n successfully genotyped leaf samples as pollen receptors | Number of leaf samples, successfully genotyped after DNA isolation, that were considered as pollen receptors. |
| n successfully genotyped rhizome samples genotyped as offspring | Number of rhizome samples, successfully genotyped after DNA isolation, that were considered as pollen receptors. |
| n samples removed from n successfully genotyped samples because of missing values <1 | Number of leaf and rhizome samples that were removed because of too many missing values. |
| n samples included in clonality test among shoot clusters | Number of leaf and rhizome samples included in the test of clonality among shoot clusters. |
| n distinct MLGs | Number of distinct multilocus genotypes identified in the clonality test among shoot clusters. |
| n identified clones among shoot clusters | Number of clones identified in the clonality test among shoot clusters. |
| n shoot clusters included in clonality test within shoot clusters | Number of shoot clusters that were included for the clonality test within shoot clusters (see Table S4.2 and S4.3 for details). |
| n genotyped leaf samples included in clonality test within shoot clusters | Number of all multilocus genotypes included in the clonality test within shoot clusters (see Table S4.2 and S4.3 for details). |
| n distinct identified MLGs in clonality test within shoot clusters | Number of distinct multilocus genotypes included in the clonality test within shoot clusters (see Table S4.2 and S4.3 for details). |
| n additional genotypes from clonality test within shoot clusters | Number of additional distinct multilocus genotypes identified through the within shoot cluster clonality test. |
| n all samples after Step 2 | Number of remaining genotypes after the checks of step 2 (Table S2.3) |
| Step 3 Paternity analysis |  |
| n MLGs included in paternity analysis | Total number of all multilocus genotypes that were included in the paternity analysis. |
| n potential pollen donor MLGs | Number of all multilocus genotypes that were included in the paternity analysis as potential pollen donors (including pollen receptors). |
| n pollen receptors MLGs | Number of all multilocus genotypes that were included in the paternity analysis as pollen receptors. |
| n offspring MLGs | Number of all multilocus genotypes that were included in the paternity analysis as offspring with known pollen receptors. |
| Step 4 Data analysis |  |
| n pollen receptors included in testing H1, H3, H4 | Number of pollen receptors with more than five offspring, for which *PF_within_* and allelic richness were calculated. |
| n shoot clusters included in testing H2 | Number of included shoot clusters with known shoot numbers. |

**Samples removed in Step 2: Quality and clonality checks**

**Table S2.2:** The table summarizes how many potential pollen donors, pollen receptors and offspring samples were excluded due too many missing values, duplicate occurrences of identical multi-locus genotypes, or more than two mismatches between the genotypes of the pollen receptor and the offspring.

| **Forest Patch ID** | **Missing value > 1** | **Clonality test** | **Mismatches between pollen receptor and offspring > 2** |
| --- | --- | --- | --- |
| F01 | 5 potential pollen donors | 18 potential pollen donors | none |
| F04 | none | 46 potential pollen donors  1 offspring | none |
| F06 | 2 potential pollen donors  1 offspring | 55 potential pollen donors | 1 offspring |
| F07 | 1 offspring | 16 potential pollen donors | none |
| F08 | none | 8 potential pollen donors  1 offspring | none |
| F10 | 1 potential pollen donor  2 offspring | 40 potential pollen donors  1 offspring | none |
| F45 | 1 offspring | 4 potential pollen donors  2 offspring | none |
| F48 | none | 73 potential pollen donors  1 offspring | none |
| F51 | 1 offspring | 5 p potential pollen donors  1 pollen receptor  5 offspring | 1 offspring |
| Total | 8 potential pollen donors  6 offspring | 265 potential pollen donors  11 offspring  1 pollen receptor | 2 offspring |

**Table S2.3 Column descriptions:** The attached Excel file, “Table S2.3 All_pollen_donors.xlsx”, provides information for each sampled shoot cluster.

| Column name | Description |
| --- | --- |
| Shoot_Cluster_ID | Unique identifier for each shoot cluster |
| Forest_Patch_ID | Unique identifier of the forest patch |
| No_shoots | Number of shoots in the shoot cluster counted during the May campaign 2018 |
| No_sampled | Number of shoots in the shoot cluster that were sampled |
| Date_Sampling | Date when the leaf material was collected |
| Longi_WGS84 and Lati_WGS84 | The GPS coordinates of the shoot cluster (WGS84) |

# Supplement 3: Stratification and germination

| **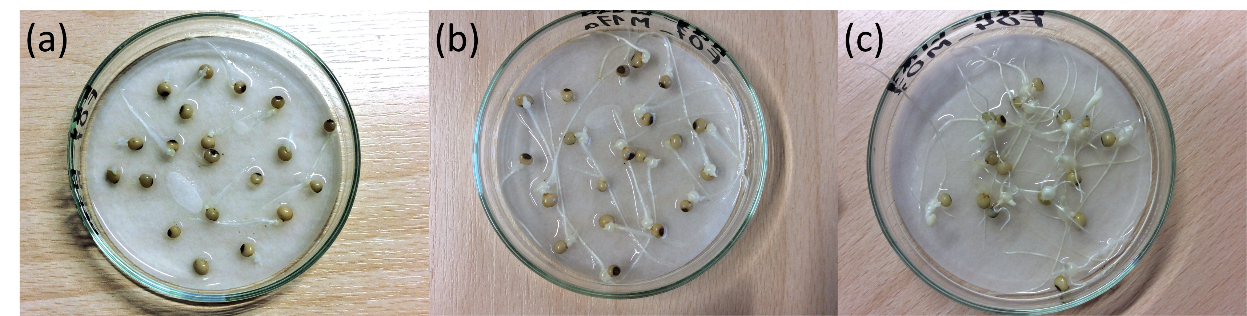** |  |  |
| --- | --- | --- |

**Figure S3:** Photographs after stratification: (a) 3.5 weeks, (b) 5 weeks, and (c) 10 weeks after start of germination.

**Protocol:**

1. We extracted seeds from the fruits and thoroughly cleaned them from the fruit pulp.
2. We included a total of 1442 seeds in the stratification and germination procedure. They originated from 91 different pollen receptors (6 to 14 pollen receptors per forest patch; 1 to 46 seeds per pollen receptor; mean: 17)
3. We placed the 20 seeds on wet filter paper (soaked with distilled water) on Petri dishes with a diameter of 88 mm. The Petri dishes were covered with a lid to reduce evaporation.
4. We exposed the seeds to a cold-wet stratification, i.e., we placed the Petri dishes in a climate chamber at 5°C for six weeks and kept the filter paper wet during this time.
5. After six weeks, the Petri dishes were kept in a closed box at room temperature (around 20°C) for another seven weeks. The filter paper was kept moist during this time.
6. We obtained a total number of 1006 seedlings, which indicates a germination rate of 0.70.

# Supplement 4: Genotyping, quality and assessment of clonality

**Table S4.1:** The table describes the seven different microsatellite primers used in this study. It reports the size ranges in base pairs [bp], the number of alleles and genotypes, the amount of missing values and pollen receptor-offspring mismatches, as well as the genotyping error rates based on 10 % repetition as well as on pollen receptor-offspring mismatches.

| Primer name | Range  [bp] | Alleles  [N] | Genotypes  [N] | Percentage of missing value  [%] |  | Error rate based on 10% repetition [%] | Receptor-offspring mismatch or NA  [N] | Error rate based on Receptor-offspring mismatches [%] |
| --- | --- | --- | --- | --- | --- | --- | --- | --- |
| Pmu024^1^ | 125-129 | 2 | 2 | 0 |  | 0.61 | 0 | 0 |
| Pmu091^1^ | 243-276 | 12 | 53 | 0.19 |  | 0.31 | 2 | 0.21 |
| Pc33^2^ | 233-277 | 22 | 164 | 0.34 |  | 0.31 | 8 | 0.84 |
| Pc25^2^ | 215-263 | 15 | 67 | 0.49 |  | 0 | 4 | 0.42 |
| Pmu008^1^ | 187-190 | 2 | 4 | 0.68 |  | 1.22 | 0 | 0 |
| Pmu373^1^ | 100-106 | 2 | 4 | 0.15 |  | 0.92 | 0 | 0 |
| Pt09^2^ | 126-216 | 42 | 448 | 0.52 |  | 1.22 | 9 | 0.95 |

^1^ Newly developed for this study

^2^ Developed for *P. cyrtonema* by Cheng et al. (2010) and for *P. filipes* by Liu et al. (2010).

**References**

Cheng WJ, Liu TT, Wu HL, Zhou SB, Xuan SQ, Zhu GP. Isolation and characterization of twelve polymorphic microsatellite loci in *Polygonatum cyrtonema* and cross-species amplification. Conserv Genet Resour. 2010; 2:105-107. <https://doi.org/10.1007/s12686-010-9218-1>

Liu TT, Cheng WJ, Zhou SB, Shao JW, Wu HL, Zhu GP. Eleven polymorphic microsatellite loci in Polygonatum filipes and cross-amplification in other congeneric species. Conserv Genet Resour. 2010; 2:77-79. <https://doi.org/10.1007/s12686-010-9179-4>

**Table S4.2 Column descriptions:** The attached Excel file, “Table S4.2 Allele table”, contains two sheets: “Alleles”, which lists allele data for each sample at specific loci, and “Sample_info”, which provides additional sample information.

| Column name | Description |
| --- | --- |
| Both sheets |  |
| Sample ID | Unique identifier of the sample |
| Sheet: „Alleles“ |  |
| Rows B-O | Alleles at the following loci: Pmu024, Pmu091, Pc33, Pc25, Pmu008, Pmu373, Pt09 (See: Table S4,1). As we used codominant markers, two alleles are recorded per locus and sample, indicated by “_1” and “_2”. Missing values are coded as “-999”. |
| Sheet “Sample_info” |  |
| Pollen_receptor_ID | Identifier of the pollen receptor to which the sample belonged. This information is only relevant for samples with the purpose “Offspring” or “Pollen_receptor”. |
| Shoot_cluster_ID | Identifier of the shoot cluster from which the sample was taken. |
| Forest_patch | Identifier of the forest patch from which the sample was taken. |
| Purpose | Original purpose for which the sample was collected: “pollen receptor”, “pollen donor”, “offspring”, and “Clone test” = (sampled to test within-shoot-cluster clonality). |
| For_clone_check | Indicated samples included in the test of within shoot cluster clonality, including samples originally classified as “Pollen_donor”. |
| Year | Year when the sample was collected. |

**Clonality among shoot clusters**


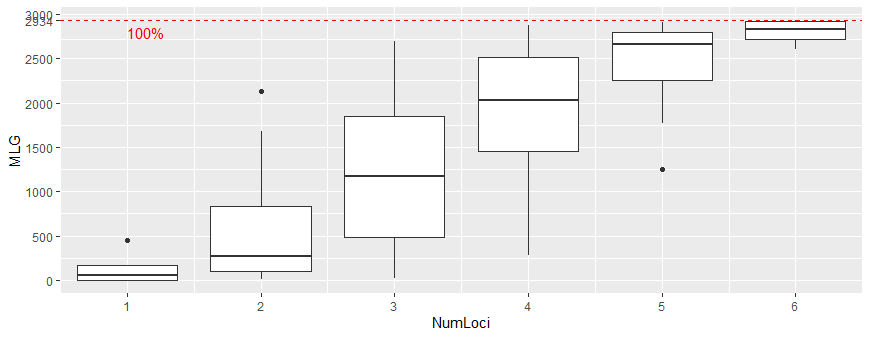


**Figure S4.1:** Multi locus genotype (MLG) accumulation curve based on 200 subsamples for each number of loci.

**Clonality within shoot clusters**

**Table S4.3:** Detailed overview of the shoots analyzed in the comparisons of shoot clusters of different sizes. Reported are numbers of flowering shoots per cluster, the number of collected samples, the number of samples that were genotyped, and the number of identified multi locus genotypes (MLGs).

| **Polygonatum patch** | **Flowering shoots** | **Samples collected in the field** | **Genotyped samples** | **MLG** |
| --- | --- | --- | --- | --- |
| F01_P22 | 2 | 2 | 2 | 1 |
| F04_P37 | 2 | 2 | 2 | 1 |
| F04c_P09 | 2 | 2 | 2 | 2 |
| F06b_P37 | 2 | 2 | 2 | 1 |
| F10b_P40 | 2 | 2 | 2 | 2 |
| F04a_P16 | 3 | 3 | 3 | 2 |
| F06_P03 | 3 | 3 | 3 | 1 |
| F48d_P31 | 3 | 3 | 3 | 3 |
| F01a_P39 | 4 | 2 | 2 | 1 |
| F04b_P25 | 4 | 2 | 2 | 2 |
| F06e_P42 | 4 | 2 | 2 | 1 |
| F10a_P22 | 4 | 2 | 2 | 1 |
| F10c_P37 | 4 | 2 | 2 | 1 |
| F04_P05 | 6 | 3 | 3 | 1 |
| F10c_P04 | 6 | 3 | 3 | 2 |
| F48b_P17 | 6 | 2 | 2 | 1 |
| F48j_P24 | 6 | 3 | 3 | 1 |
| F48k_P57 | 6 | 5 | 5 | 5 |
| F07a_P55 | 7 | 2 | 2 | 2 |
| F10_P35 | 7 | 2 | 2 | 1 |
| F48f_P22 | 7 | 2 | 2 | 1 |
| F10c_P45 | 8 | 2 | 2 | 1 |
| F04c_P02 | 9 | 5 | 5 | 3 |
| F10b_P52 | 10 | 2 | 2 | 1 |
| F48_P58 | 10 | 3 | 3 | 2 |
| F48a_P34a | 10 | 2 | 2 | 2 |
| F07b_P17 | 11 | 2 | 2 | 1 |
| F48c_P08 | 11 | 3 | 3 | 2 |
| F01a_P30 | 12 | 5 | 5 | 3 |
| F06a_P43 | 12 | 2 | 2 | 1 |
| F10d_P13 | 12 | 2 | 2 | 1 |
| F48d_P04 | 12 | 3 | 3 | 2 |
| F01a_P53 | 14 | 5 | 5 | 3 |
| F04c_P47a | 15 | 8 | 8 | 4 |
| F08a_P19 | 15 | 2 | 2 | 1 |
| F48e_P30 | 16 | 3 | 3 | 2 |
| F48i_P25 | 16 | 2 | 2 | 2 |
| F10_P51 | 17 | 3 | 3 | 2 |
| F10b_P49b | 17 | 2 | 2 | 1 |
| F04c_P41 | 18 | 3 | 3 | 2 |
| F06e_P48a | 18 | 2 | 2 | 1 |
| F06d_P19 | 20 | 5 | 5 | 1 |
| F48_P42b | 21 | 3 | 3 | 2 |
| F01b_P10 | 22 | 5 | 5 | 1 |
| F10_P43 | 22 | 2 | 2 | 2 |
| F06e_P01 | 24 | 8 | 8 | 1 |
| F10a_P46 | 25 | 3 | 3 | 2 |
| F48c_P43 | 27 | 3 | 3 | 2 |
| F10b_P04 | 33 | 5 | 5 | 2 |
| F48e_P20 | 36 | 5 | 4 | 3 |
| F06b_P39 | 37 | 8 | 8 | 3 |
| F10c_P13 | 46 | 5 | 4 | 1 |
| F04d_P32 | 51 | 8 | 8 | 1 |
| F10c_P44 | 51 | 5 | 5 | 4 |
| F48e_P11 | 67 | 8 | 8 | 7 |
| F48k_P32 | 69 | 10 | 10 | 6 |
| F06e_P38 | 103 | 13 | 11 | 7 |
| F48i_P17 | 119 | 16 | 16 | 14 |
| F07_P03b | 190 | 25 | 25 | 17 |
| Total | 1316 | 249 | 245 | 144 |

**Table S4.4:** The table summarizes how many shoot clusters per size class were included in the assessment of within-shoot-cluster clonality and the proportion of them that were monoclonal.

|  | Number of included shoot clusters | Monoclonal shoot clusters |
| --- | --- | --- |
| All | 59 | 26 (44%) |
| ≤ 5 flowering shoots | 13 | 8 (62%) |
| 5-20 flowering shoots | 28 | 13 (46%) |
| > 20 flowering shoots | 18 | 5 (28%) |

With a higher number of sampled shoots (larger shoot clusters) the percentage of monoclonal shoot clusters is decreasing.

# Supplement 5: Information on quality of paternity analysis

**Table S5.1:** The table shows the reproducibility of the pollen-donor-assignment procedure across independent runs. It indicates how many pollen donor-offspring pairs were consistently assigned in n out of five total runs. In cases where different pollen donors were assigned to the same offspring, assignments were not considered reproducible unless one donor appeared at least in one run more than the other(s), in such cases the dominant donor was considered as the assigned pollen donor. The mean assignment probabilities for pollen donor-offspring pairs, as reported by COLONY 2 are summarized in the rightmost column across different numbers of seeds for all pairs identified in multiple runs.

| Forest Patch | Number of runs | | | | | Differing pollen donors | Total | Total of reliable assigned offspring | Mean, minimum and maximum probability |
| --- | --- | --- | --- | --- | --- | --- | --- | --- | --- |
|  | 1 | 2 | 3 | 4 | 5 |  |  |  |  |
| F01 |  |  |  | **1** | **8** | **0** | **9** | **9** | 0.82 (range: 0.81-0.89) |
| F04 | **4** | **2** |  | **5** | **18** | **0** | **29** | **25** | 0.94 (range: 0.74-1) |
| F06 | **2** | **12** | **3** | **14** | **3** | **0** | **34** | **32** | 0.87 (range: 0.42-1) |
| F07 | **17** | **7** | **5** |  | **4** | **3** | **33** | **16** | 0.95 (range: 0.88-0.99) |
| F08 | **3** | **15** | **1** |  | **23** | **0** | **42** | **39** | 0.82 (range: 0.59-1) |
| F10 | **1** |  | **3** | **23** | **9** | **1** | **36** | **35** | 0.84 (range: 0.7-1) |
| F48 |  |  |  |  | **12** | **0** | **12** | **12** | 1 (range: 1-1) |
| F51 | **1** |  |  | **1** | **5** | **0** | **7** | **6** | 1 (range: 1-1 |
| Total | **28** | **36** | **12** | **44** | **82** | **4** | **202** | **174** |  |

# Supplement 6: Landscape metrics

**Table S6.1:** Landscape metrics used to quantify the landscape composition in buffers with radii of 50 m, 250 m, and 1000 m around pollen receptor plants.

| Area-based metrics | Buffer radius [m] | | Median, minimum and maximum percentage cover [%] of buffer |
| --- | --- | --- | --- |
| Deciduous Forest | 50 | 76 (range: 31.5-100) | |
| Deciduous Forest | 250 | 26.7 (range: 9-41.4) | |
| Deciduous Forest | 1000 | 9.5 (range: 5.7-16.8) | |
| SEMNATGRASS | 50 | 0 | |
| SEMNATGRASS | 250 | 0 | |
| SEMNATGRASS | 1000 | 0.4 (range: 0-1.3) | |
| RAPESEED | 50 | 0 | |
| RAPESEED | 250 | 0 | |
| RAPESEED | 1000 | 0.9 (range: 0-21.4) | |
| MAIZE | 50 | 0 (range: 0-73) | |
| MAIZE | 250 | 7.8 (range: 0-71.4) | |
| MAIZE | 1000 | 22.3 (range: 5-47.5) | |
| Linear landscape elements |  | **Median, minimum and maximum length in meters [m]** | |
| L_ROAD | 50 | 0 (range: 0-162) | |
| L_ROAD | 250 | 317 (range: 0-1028) | |
| L_ROAD | 1000 | 5466 (range: 3649-9567) | |
| L_WATER | 50 | 0 (range: 0-153) | |
| L_WATER | 250 | 547 (range: 0-976) | |
| L_WATER | 1000 | 4995 (range: 694-8459) | |
| L_WOOD | 50 | 0 | |
| L_WOOD | 250 | 477 (range: 0-2100) | |
| L_WOOD | 1000 | 10140 (range: 2276-19455) | |


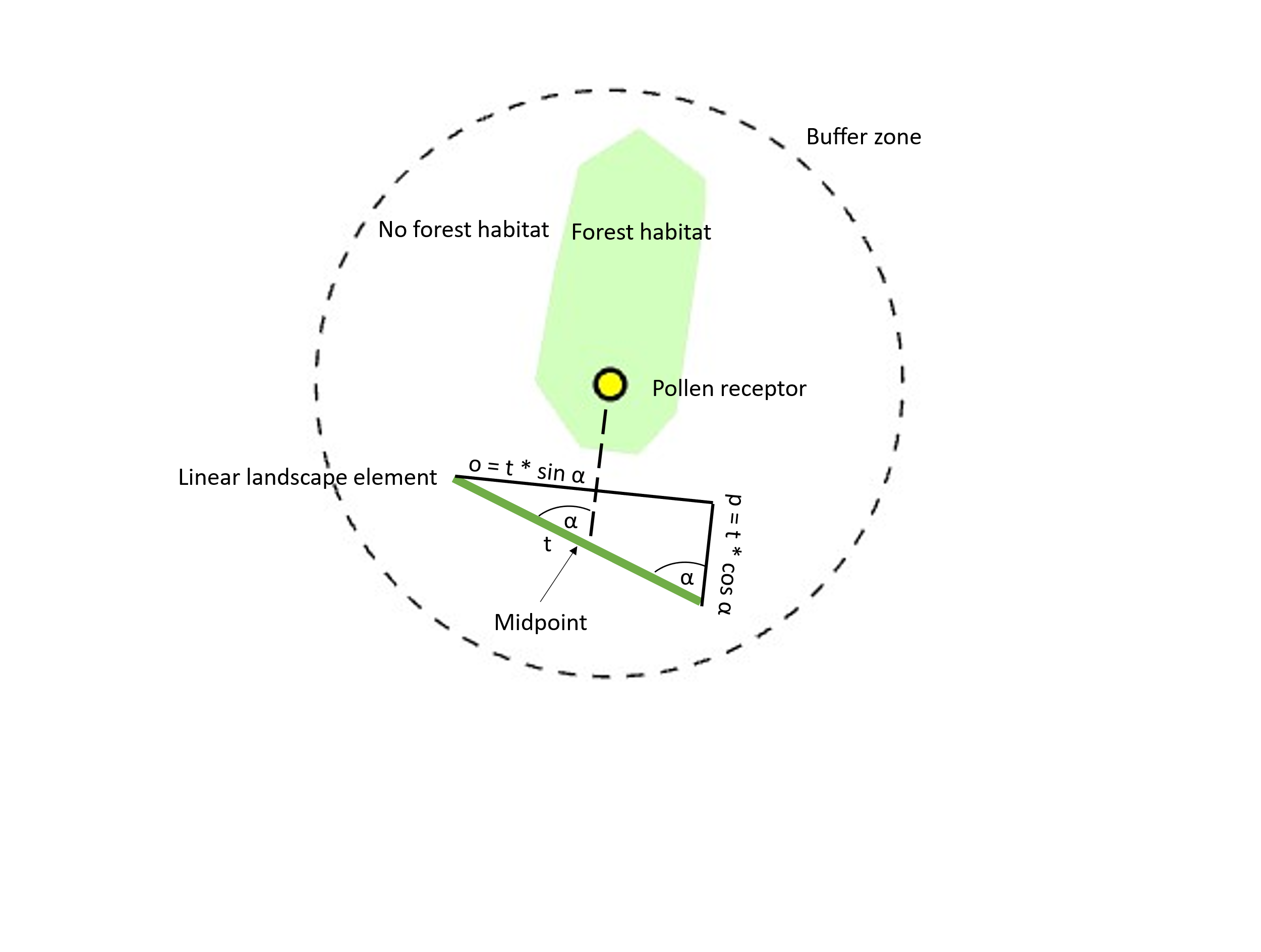


**Figure S6.1:** Schematic visualization of the calculation of the parallel (p) and orthogonal (o) length component of each specific linear landscape element.


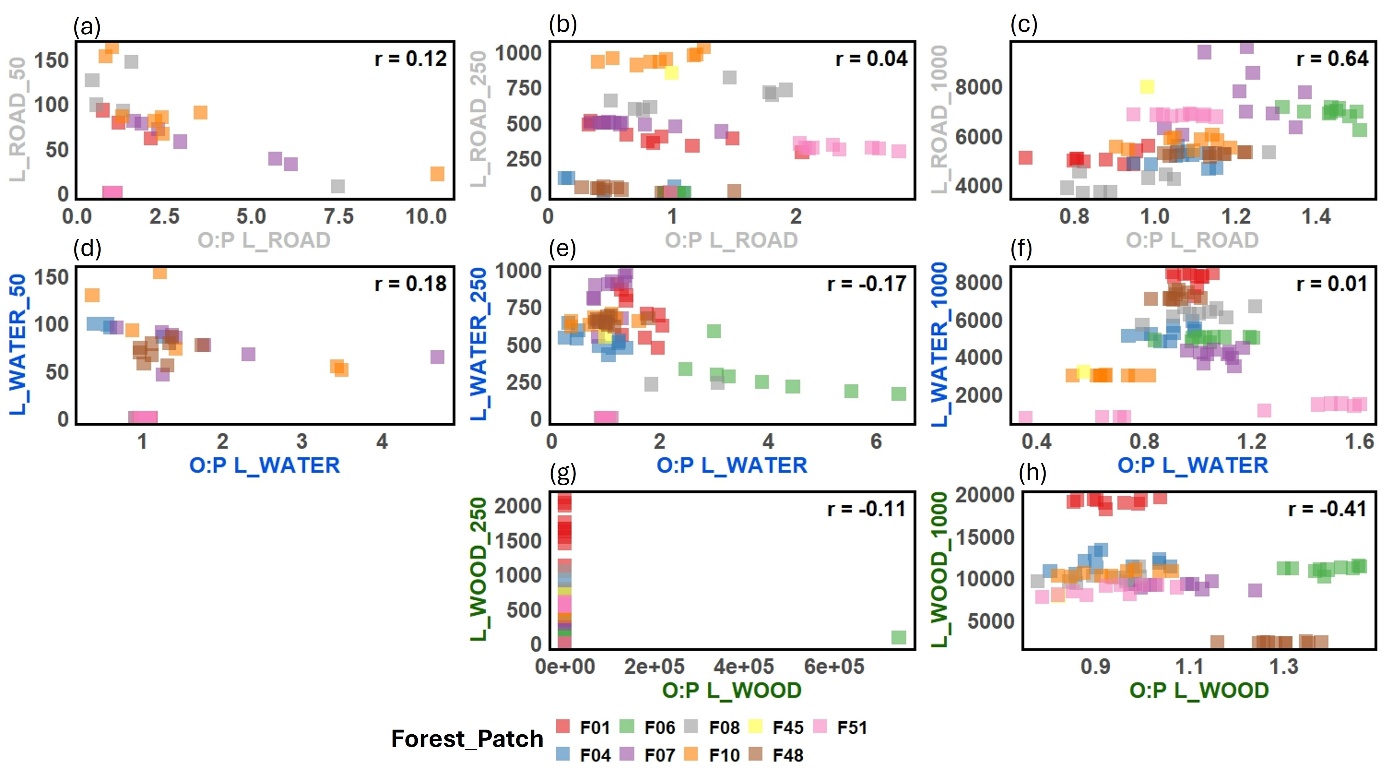


**Figure S6.2:** Distribution of the length of linear landscape elements and their orthogonal-parallel ratio at three different buffer radii. Colors indicate the forest patch from which the pollen receptor originates.

# Supplement 7: Distribution of pollen flow measures

**
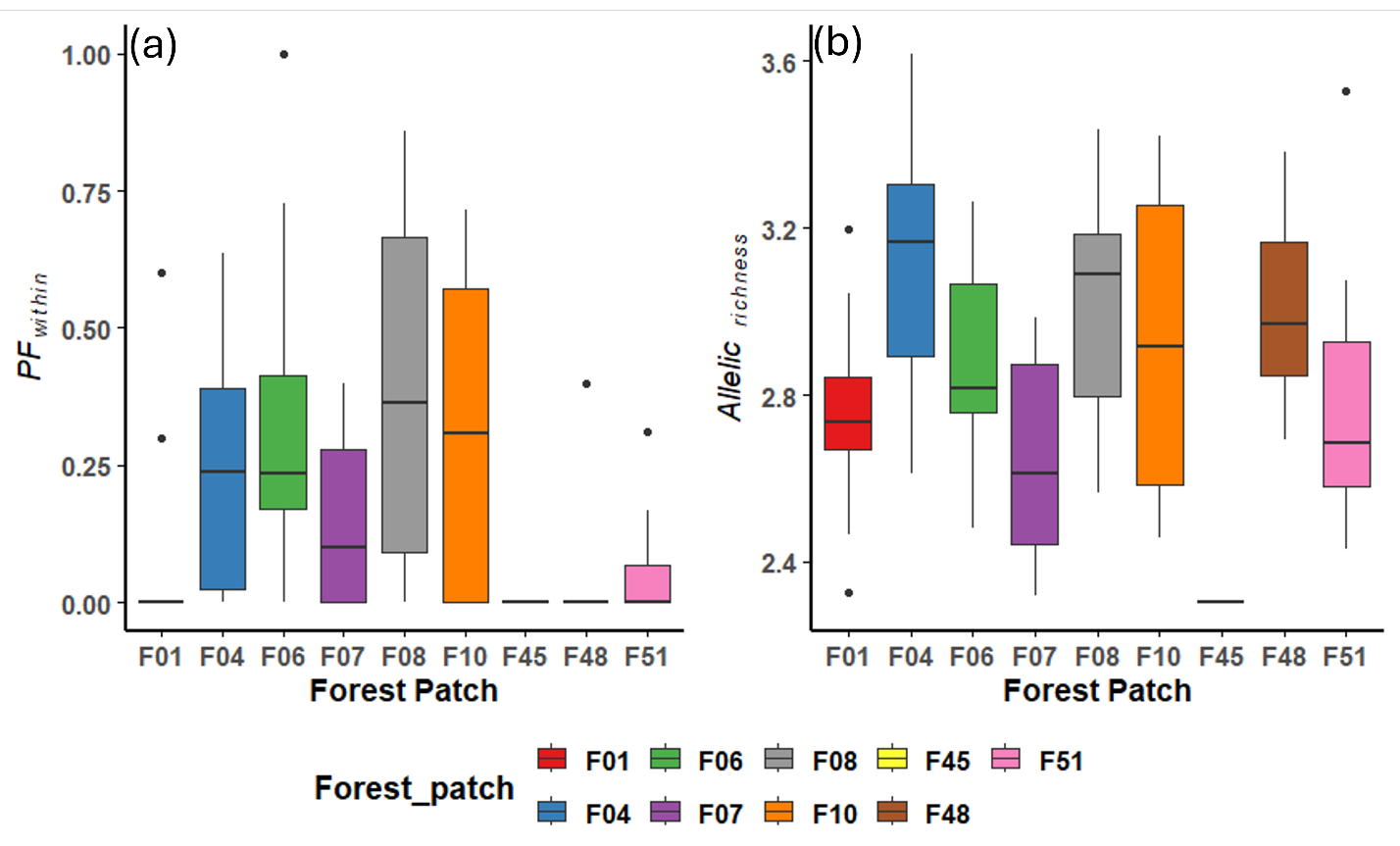
**

**Figure S7.1:** The figure shows the distribution of (a) *PF_within_* and (b) *A_r_* per pollen receptor among forest patches. Colors indicate the forest patch from which the pollen receptor originates.

**
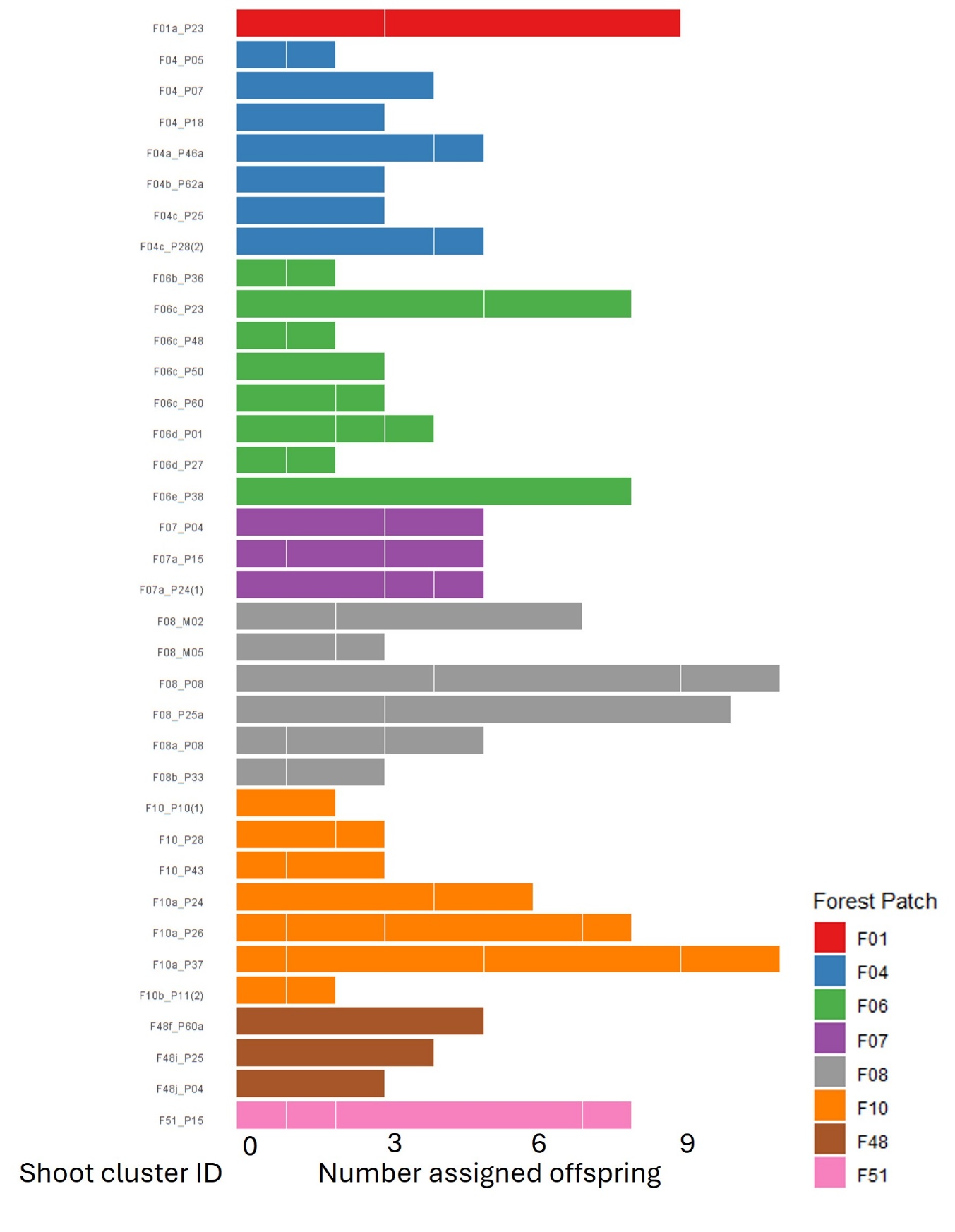
**

**Figure S7.2:** The figure shows the counts of offspring that were assigned to specific pollen donors from their own forest patch. The white vertical lines indicate from how many pollen receptors the offspring originated.

**
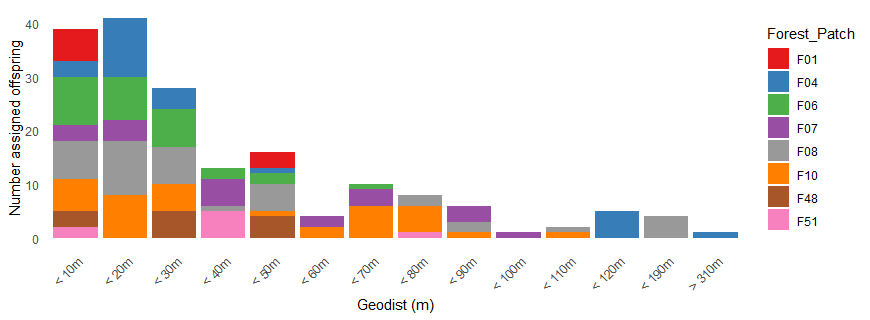
**

**Figure S7.3:** The figure shows the counts of offspring that were assigned to specific pollen donors from their own forest patch at specific geographic distances classes.

**
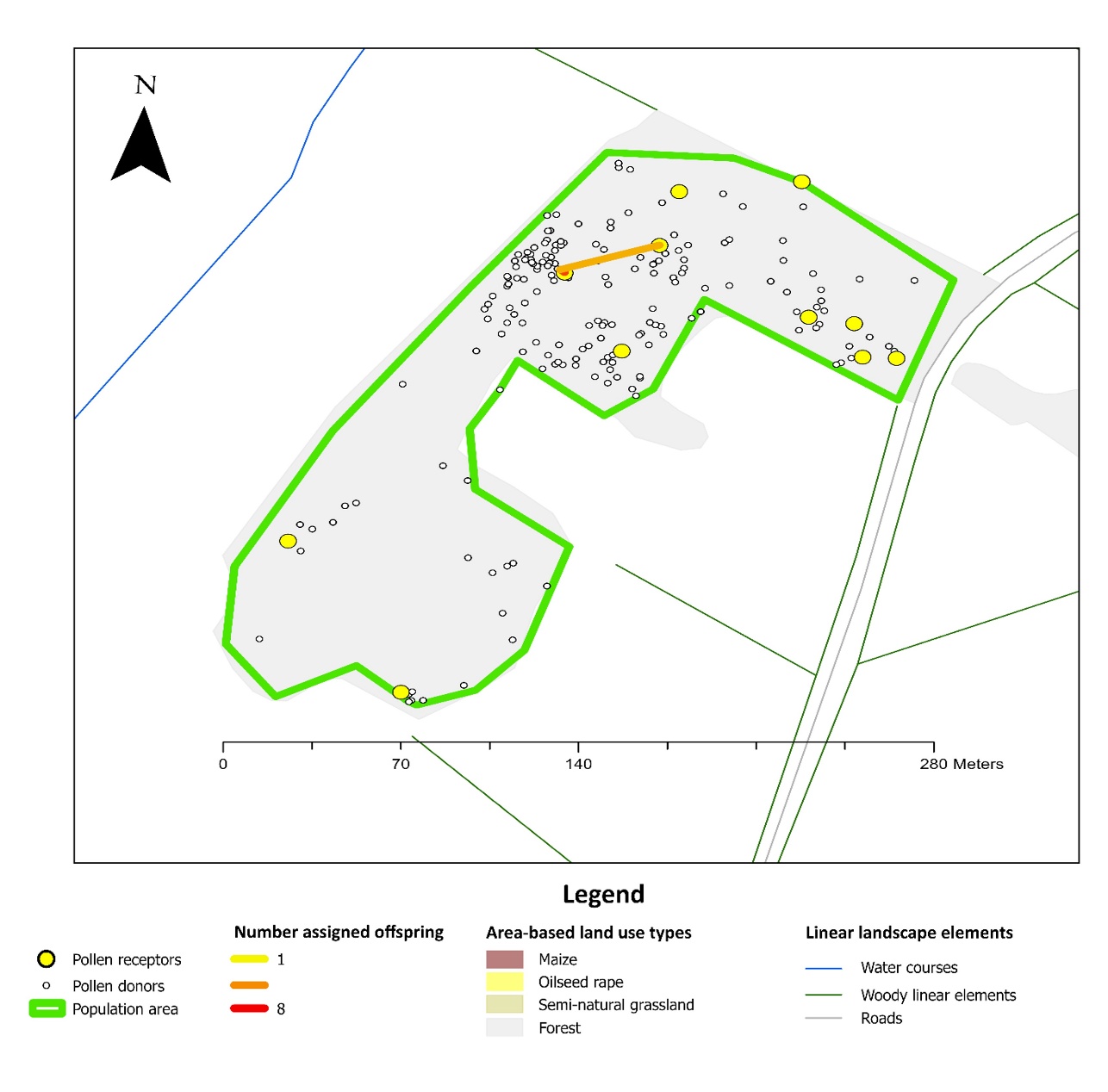
**

**Figure S7.4a:** Connection lines between pollen receptors (yellow dots) and identified pollen donors in forest patch F01. The color of the lines indicates the number of assigned offspring individuals. White areas represent land-use types not included in the study, mainly cereals and intensively managed grassland.


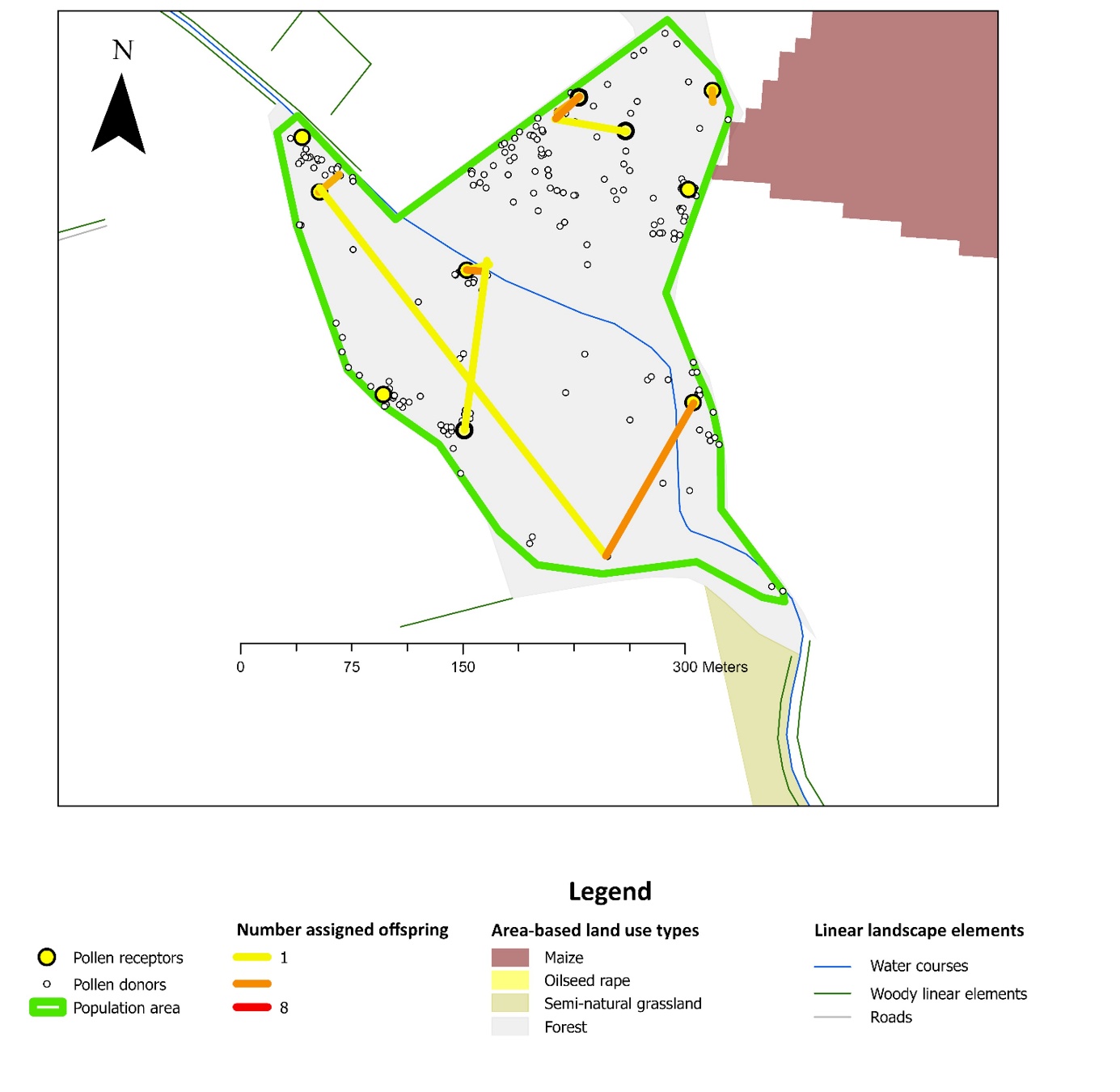


**Figure S7.4b:** Connection lines between pollen receptors (yellow dots) and identified pollen donors in forest patch F04. The color of the lines indicates the number of assigned offspring individuals. White areas represent land-use types not included in the study, mainly cereals and intensively managed grassland.


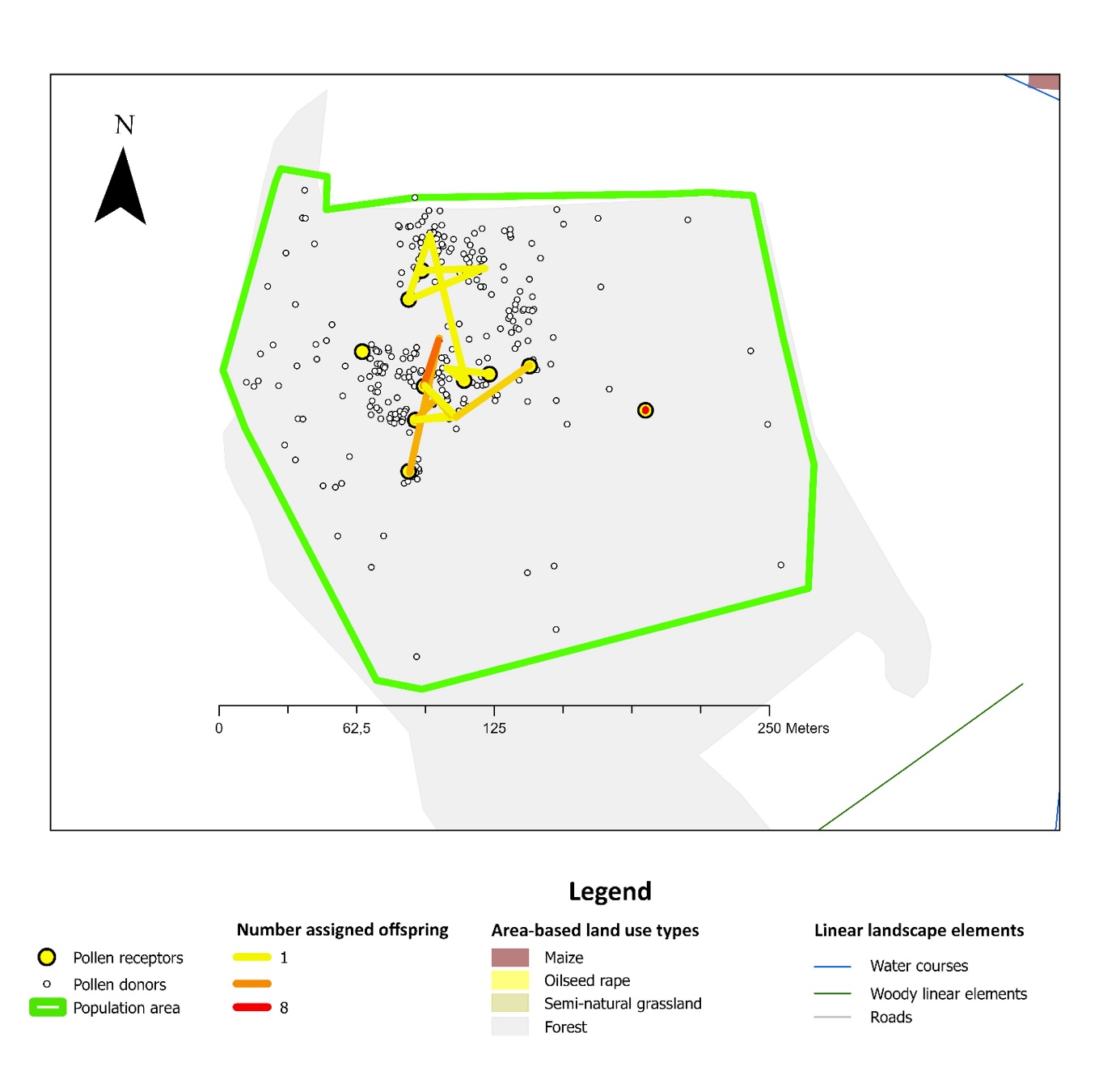


**Figure S7.4c:** Connection lines between pollen receptors (yellow dots) and identified pollen donors in forest patch F06. The color of the lines indicates the number of assigned offspring individuals. White areas represent land-use types not included in the study, mainly cereals and intensively managed grassland.


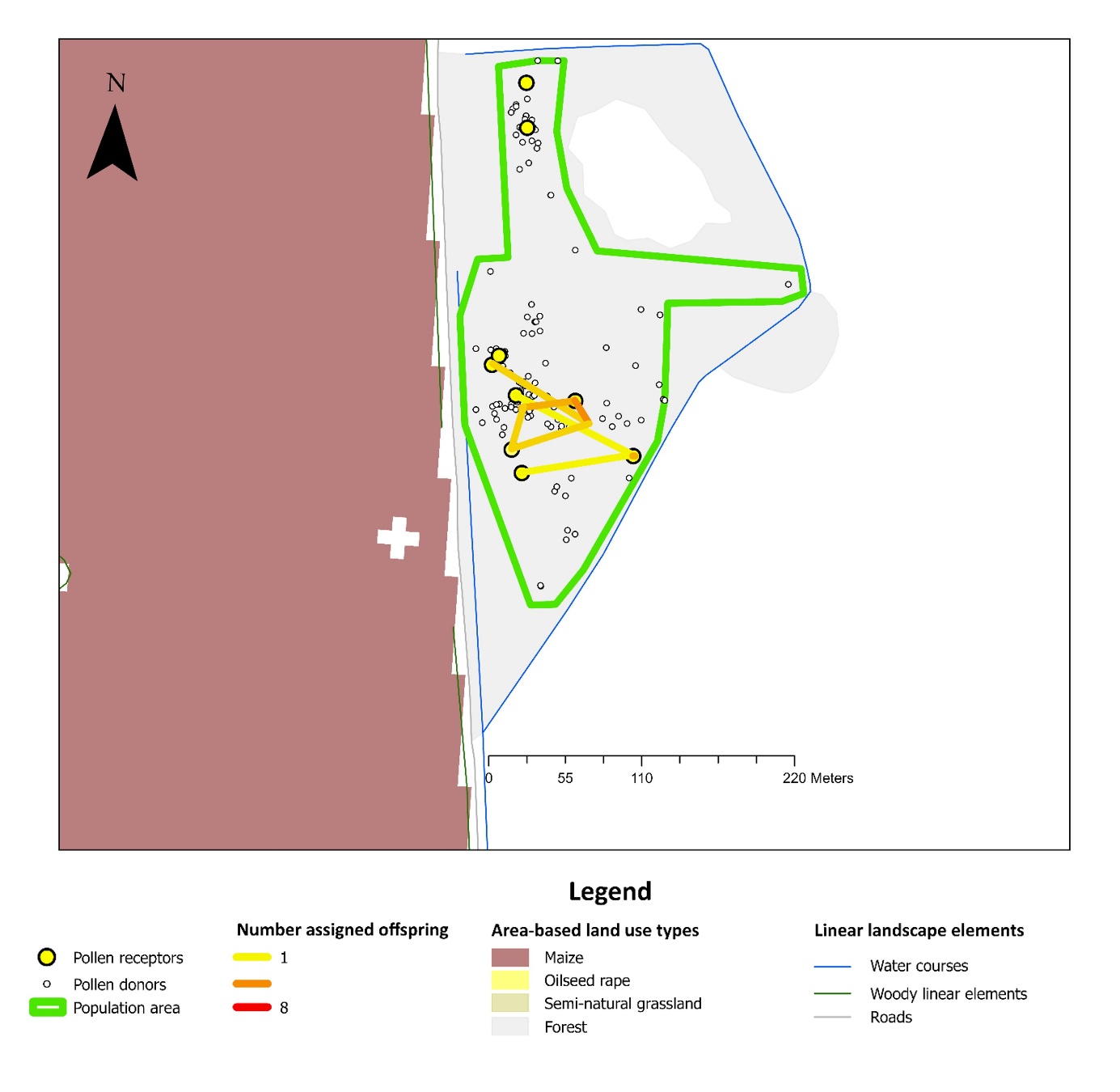


**Figure S7.4d:** Connection lines between pollen receptors (yellow dots) and identified pollen donors in forest patch F07. The color of the lines indicates the number of assigned offspring individuals. White areas represent land-use types not included in the study, mainly cereals and intensively managed grassland.


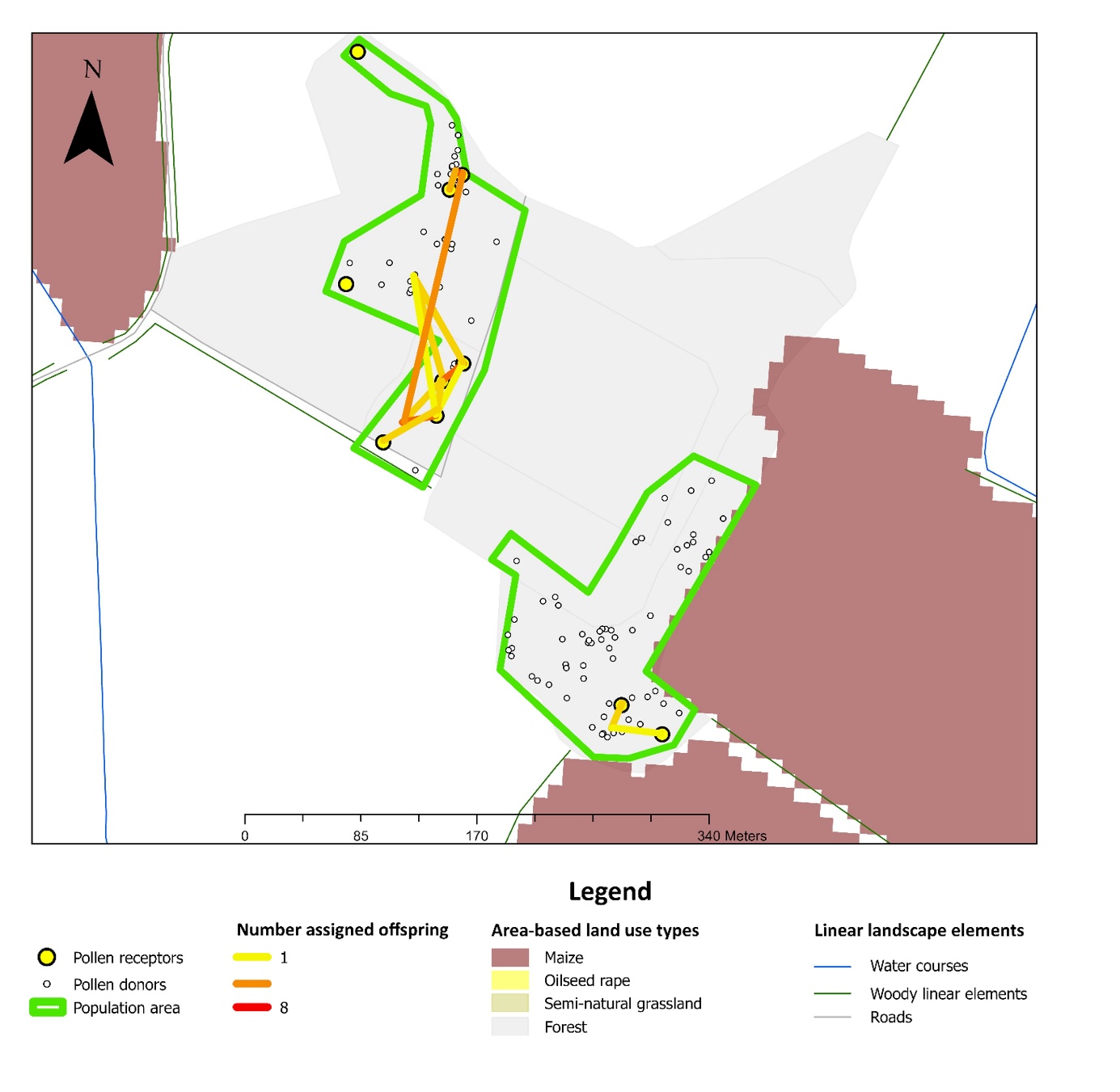


**Figure S7.4e:** Connection lines between pollen receptors (yellow dots) and identified pollen donors in forest patch F08. The color of the lines indicates the number of assigned offspring individuals. White areas represent land-use types not included in the study, mainly cereals and intensively managed grassland.


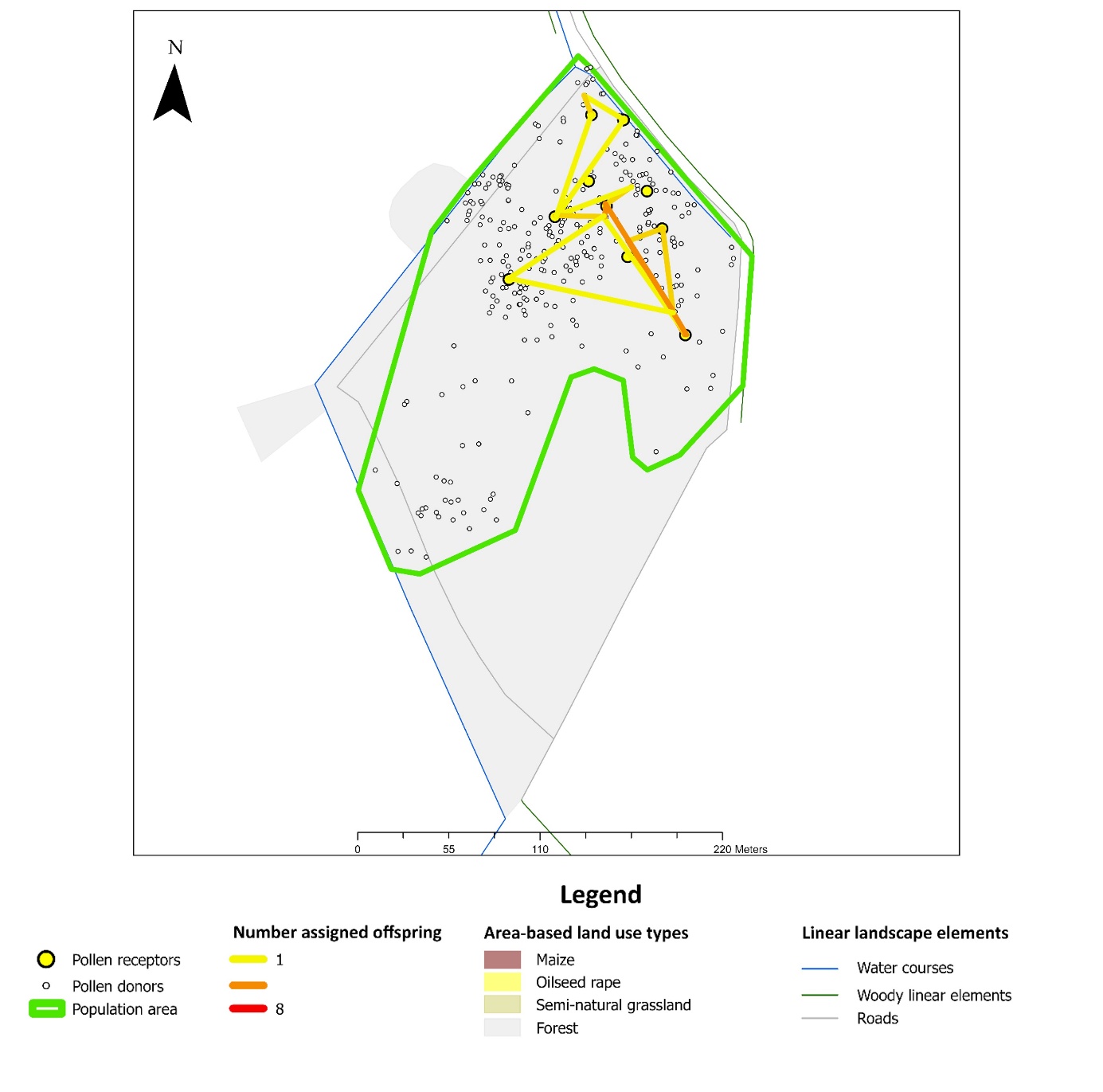


**Figure S7.4f:** Connection lines between pollen receptors (yellow dots) and identified pollen donors in forest patch F10. The color of the lines indicates the number of assigned offspring individuals. White areas represent land-use types not included in the study, mainly cereals and intensively managed grassland.


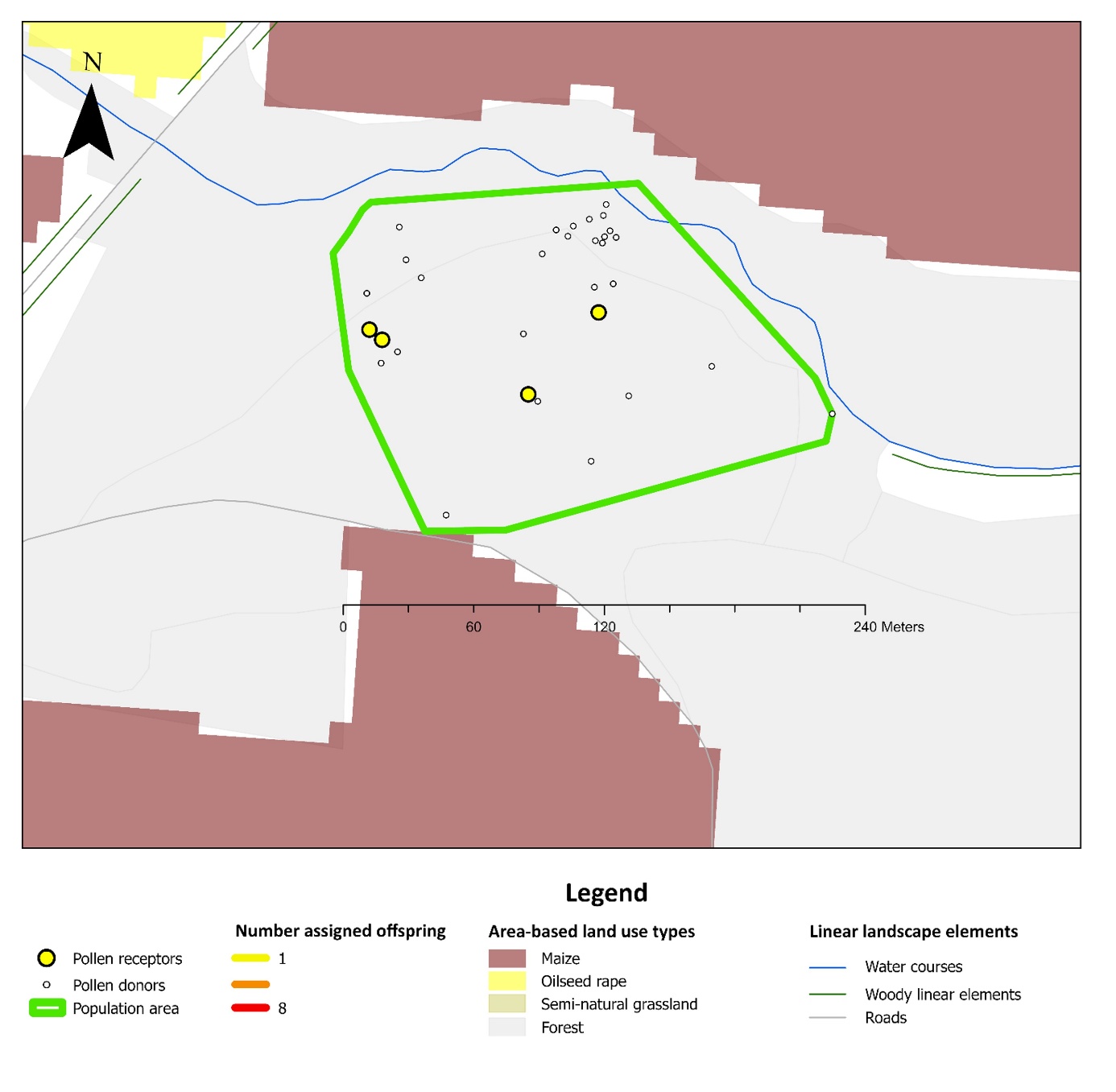


**Figure S7.4g:** Connection lines between pollen receptors (yellow dots) and identified pollen donors in forest patch F45. The color of the lines indicates the number of assigned offspring individuals. White areas represent land-use types not included in the study, mainly cereals and intensively managed grassland.


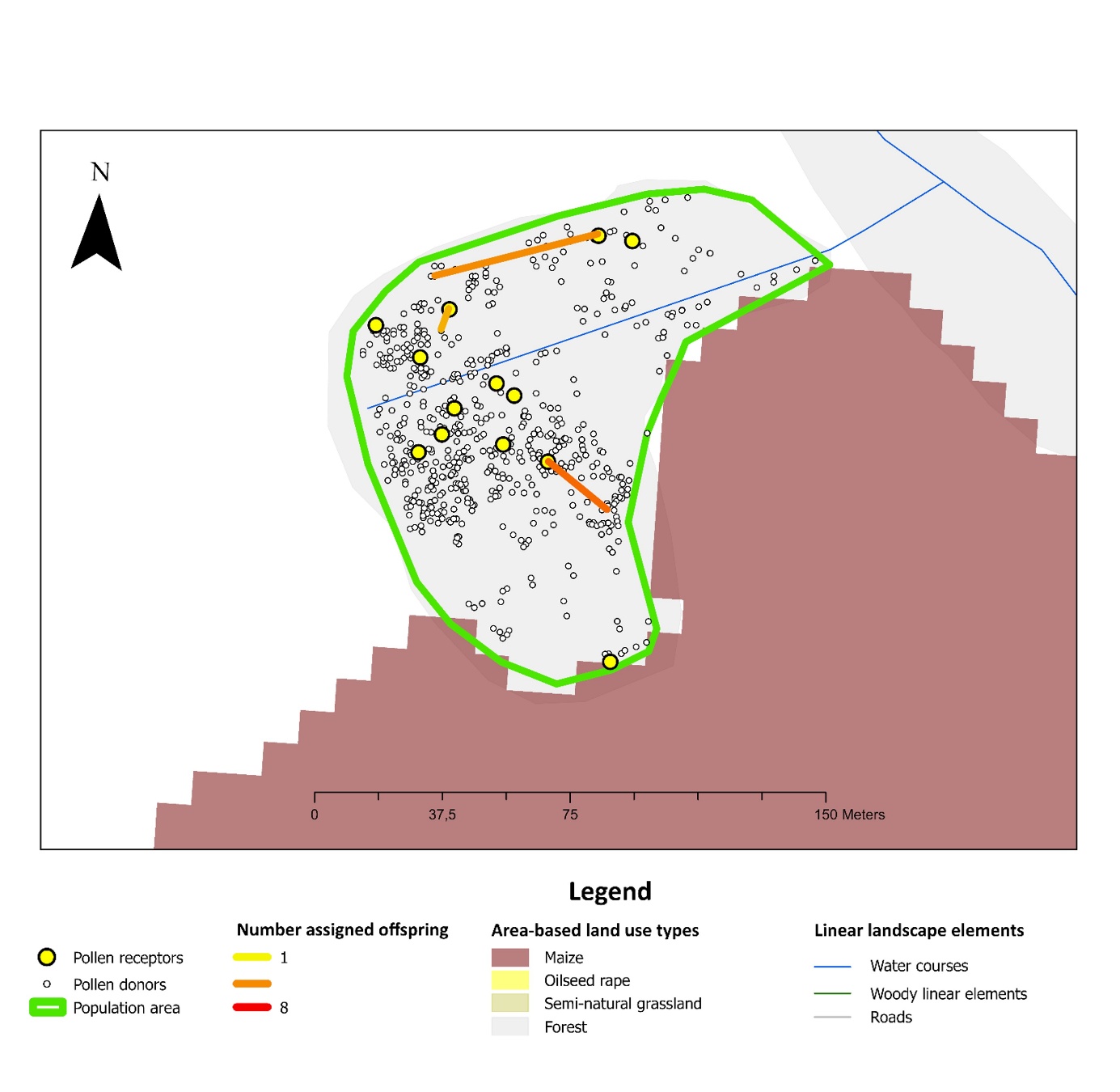


**Figure S7.4h:** Connection lines between pollen receptors (yellow dots) and identified pollen donors in forest patch F48. The color of the lines indicates the number of assigned offspring individuals. White areas represent land-use types not included in the study, mainly cereals and intensively managed grassland.


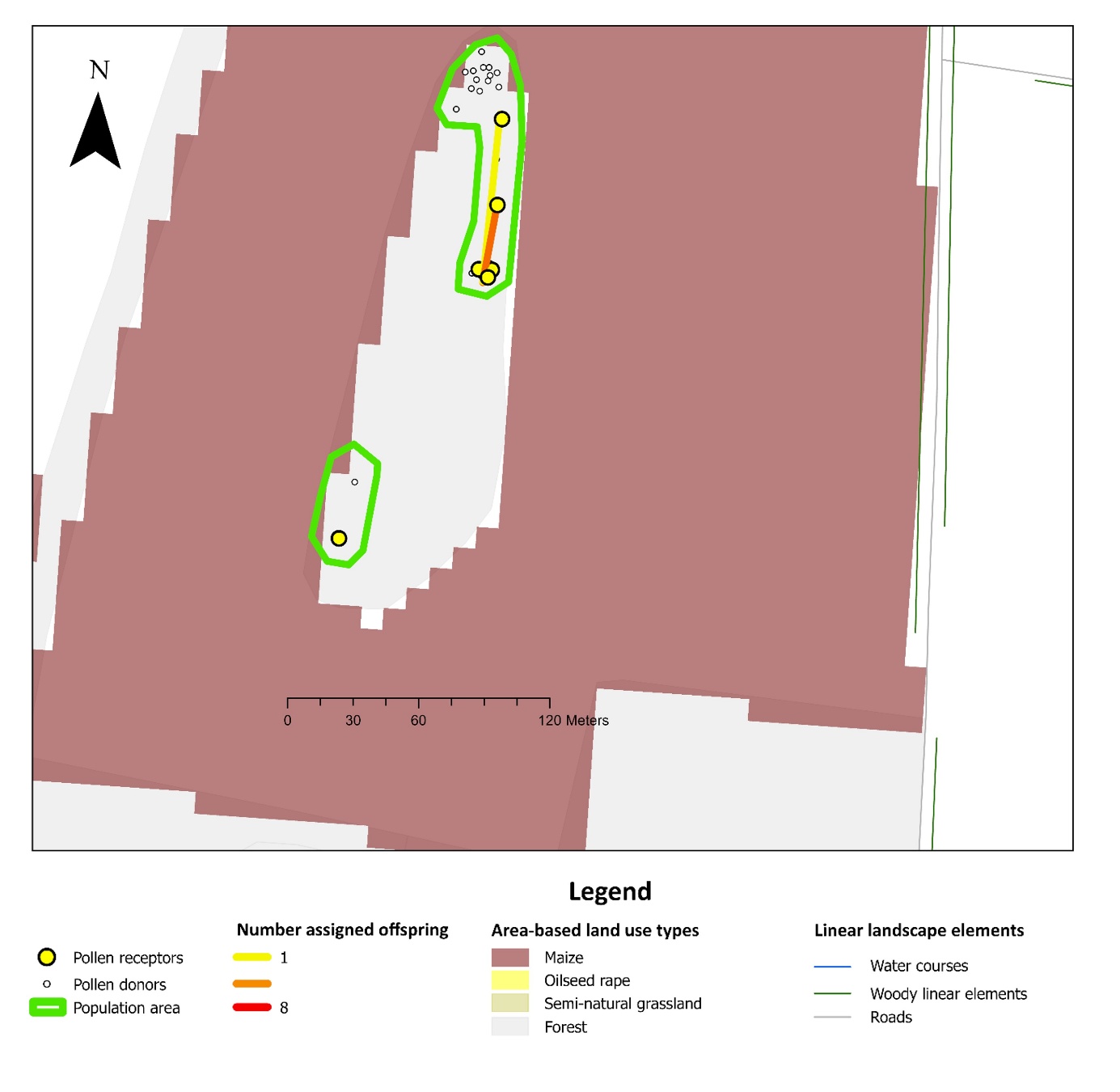


**Figure S7.4i:** Connection lines between pollen receptors (yellow dots) and identified pollen donors in forest patch F51. The color of the lines indicates the number of assigned offspring individuals. White areas represent land-use types not included in the study, mainly cereals and intensively managed grassland.

# Supplement 8: Model outcome H2

**Table S8.1:** Model outcomes of the generalized linear mixed models (GLMM) for the conditional effects to testing H2. To test the effects on within-forest patch mating patterns (H2), we used GLMM with a conditional part (Poisson distribution) and a zero-inflation part (See section Data analysis for more details).

|  | Estimate | Standard error | *z*-statistic | *p*-value |
| --- | --- | --- | --- | --- |
| (Intercept) | 1.593 | 0.096 | 16.567 | 0.000 |
| Shoot number | -0.048 | 0.059 | -0.811 | 0.418 |
| Distance_to_forest_edge | -0.057 | 0.083 | -0.691 | 0.489 |
| Interaction shoot number and distance_to_forest_edge | 0.065 | 0.038 | 1.704 | 0.088 |

**Table S8.2:** model outcomes of for the generalized linear mixed models (GLMM) for the zero-inflation effects to testing H2. To test the effects on within-forest patch mating patterns (H2), we used GLMM with a conditional part (Poisson distribution) and a zero-inflation part (See section Data analysis for more details)

|  | Estimate | Standard error | *z*-statistic | *p*-value |
| --- | --- | --- | --- | --- |
| (Intercept) | 4.118 | 0.287 | 14.344 | 0.000 |
| Shoot number | -0.262 | 0.0938 | -2.796 | 0.005 |
| Distance_to_forest_edge | -0.169 | 0.176 | -0.957 | 0.338 |
| Interaction shoot number and distance_to_forest_edge | -0.045 | 0.085 | -0.532 | 0.594 |

**
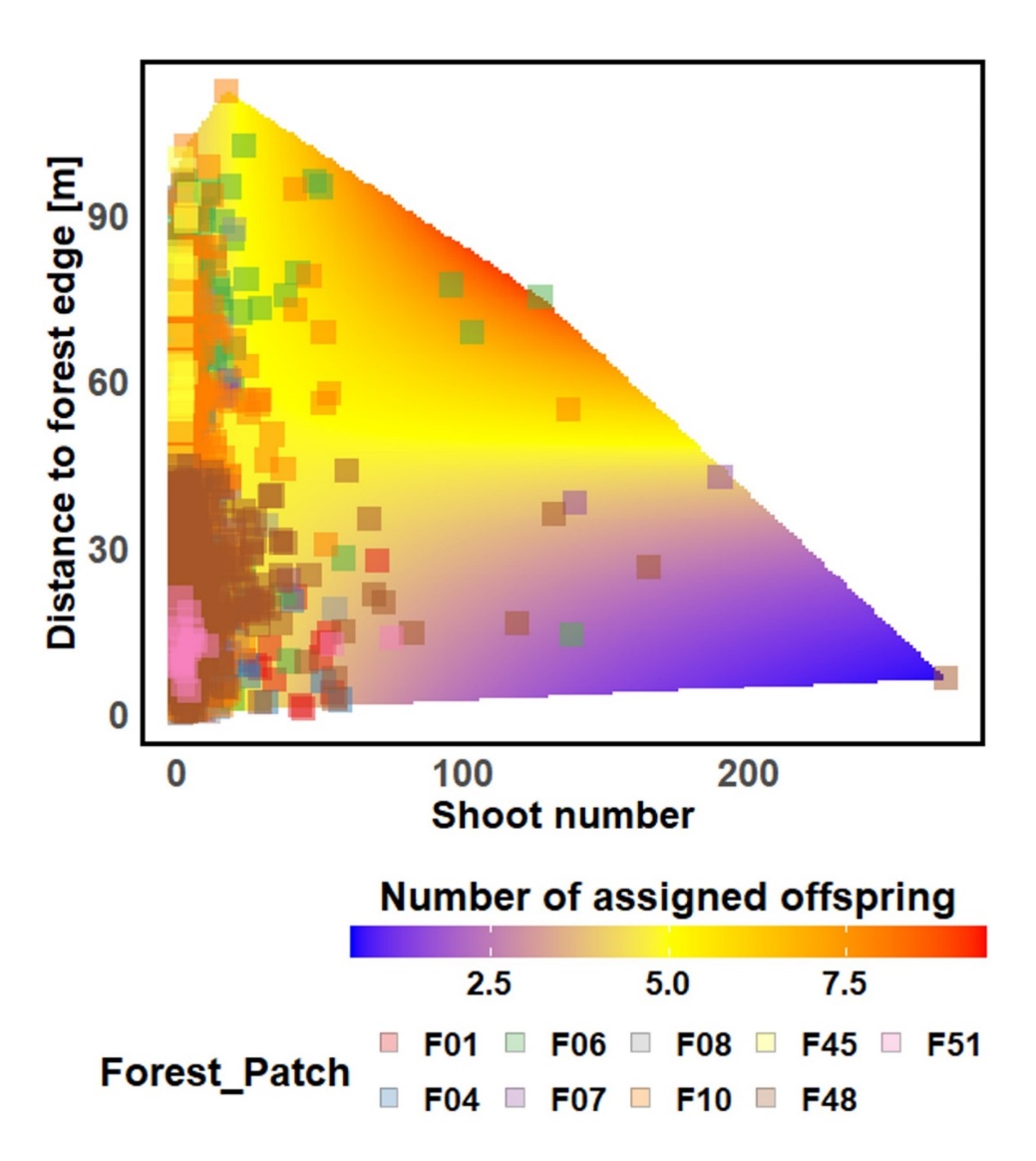
**

**Figure S8.1:** Interaction effect between shoot cluster size (shoot number) and distance to the forest edge on the number of assigned offspring (color gradient from blue to red). Colored squares represent the residuals, with colors indicating the forest patch from which the pollen receptors originated.

# Supplement 9: Collinearity among included variables

**Table S9.1:** Test results for collinearity (Pearson correlation) among all landscape metrics with a *p* < 0.15 in single-metric models for *PF_within_* in the 50 m buffer radius. Abbreviations are explained in Table 1.

| L_ROAD_50 | -0.20 |  |
| --- | --- | --- |
| O:P_ ROAD_50 | -0.07 | 0.23 |
|  | MAIZE_50 | L_ROAD_50 |

**Table S9.2:** Test results for collinearity (Pearson correlation) among all landscape metrics with a *p* < 0.15 in single-metric models for *A_r_* in the 50 m buffer radius. Abbreviations are explained in Table 1.

| L_WATER_50 | -0.14 |
| --- | --- |
|  | MAIZE_50 |

**Table S9.3:** Test results for collinearity (Pearson correlation) among all landscape metrics with a *p* < 0.15 in single-metric models for *PF_within_* in the 250 m buffer radius. Abbreviations are explained in Table 1.

| L_ROAD_250 | -0.20 |  |
| --- | --- | --- |
| N_POPULATIONS_250 | -0.33 | 0.17 |
|  | MAIZE_250 | L_ROAD_250 |

**Figure S9.4:** Test results for collinearity (Pearson correlation) among all landscape metrics with a *p* < 0.15 in single-metric models for *A_r_* in the 250 m buffer radius. Abbreviations are explained in Table 1.

| O:P_ WATER_250 | -0.17 |
| --- | --- |
|  | L_WATER_250 |

**Table S9.5:** Test results for collinearity (Pearson correlation) among all landscape metrics with a *p* < 0.15 in single-metric models for *PF_within_* cond part in the 1000 m buffer radius. Red colors indicate a correlation of |r| ≥ 0.7. Abbreviations are explained in Table 1.

| N_POPULATIONS_1000 | 0.57 |  |  |  |  |  |  |  |
| --- | --- | --- | --- | --- | --- | --- | --- | --- |
| MAIZE_1000 | -0.34 | -0.15 |  |  |  |  |  |  |
| L_WOOD_1000 | -0.31 | -0.38 | -0.54 |  |  |  |  |  |
| L_ROAD_1000 | -0.33 | -0.38 | 0.60 | -0.13 |  |  |  |  |
| L_WATER_1000 | 0.52 | 0.63 | -0.56 | 0.23 | -0.58 |  |  |  |
| O:P_WATER_1000 | -0.26 | 0.21 | 0.43 | 0.01 | 0.17 | 0.07 |  |  |
| O:P_ WOOD_1000 | 0.25 | 0.36 | 0.39 | -0.40 | 0.48 | 0.11 | 0.37 |  |
| RAPESEED_1000 | 0.24 | 0.04 | 0.32 | -0.74 | 0.32 | -0.40 | -0.13 | 0.28 |
|  | SEMNATGRASS_1000 | N_POPULATIONS _1000 | MAIZE_1000 | L_WOOD_1000 | L_ROAD_1000 | L_WATER_1000 | O:P_WATER_1000 | O:P_WOOD_1000 |

**Table S9.6:** Test results for collinearity (Pearson correlation) among all landscape metrics with a *p* < 0.15 in single-metric models for *PF_within_* zero-inflation part in the 1000 m buffer radius. Red colors indicate a Pearson correlation of |r| ≥ 0.7. Abbreviations are explained in Table 1.

| O:P_WATER_1000 | 0.07 |  |  |  |  |  |
| --- | --- | --- | --- | --- | --- | --- |
| L_ROAD_1000 | -0.58 | 0.17 |  |  |  |  |
| RAPESEED_1000 | -0.40 | -0.13 | 0.32 |  |  |  |
| L_WOOD_1000 | 0.23 | 0.01 | -0.13 | -0.74 |  |  |
| O:P_WOOD_1000 | 0.11 | 0.37 | 0.48 | 0.28 | -0.40 |  |
|  | L_WATER_1000 | O:P_ WATER_1000 | L_ROAD_1000 | RAPESEED_1000 | L_WOOD_1000 | O:P_ WOOD_1000 |

**Table S9.7:** Test results for collinearity (Pearson correlation) among all landscape metrics with a *p* < 0.15 in single-metric models for *A_r_* in the 1000 m buffer radius. Abbreviations are explained in Table 1.

| N_ POPULATIONS _1000 | -0.15 |  |  |  |
| --- | --- | --- | --- | --- |
| SEMNATGRASS_1000 | -0.34 | 0.57 |  |  |
| L_ROAD_1000 | 0.60 | -0.38 | -0.33 |  |
|  | MAIZE_1000 | N_ POPULATIONS _1000 | SEMNATGRASS_1000 | L_ROAD_1000 |

# Supplement 10: Outcome of Model averaging for H3 and H4

**Table S10.1:** Overview of model averaging. Abbreviations are explained in Table 1. Effects on *A_r_* were modeled with LMM and on *PF_within_* with GLMM with a conditional (cond) and zero-inflation part (zi). Shown are the total number of tested models and the number of models with *ΔAICc* < 2.

|  | Buffer radius | Variables from single metric models | Total number of models | Number of models with *ΔAICc* < 2 |
| --- | --- | --- | --- | --- |
| *PF_within_* | 50 | Cond:   - MAIZE - L_ROAD - O:P_ROAD   ZI:  --- | 9 | 1 |
| *A_r_* | 50 | - MAIZE - L_WATER | 4 | 2 |
| *PF_within_* | 250 | Cond:   - MAIZE - L_ROAD   ZI:   - N_POPULATIONS | 16 | 1 |
| *A_r_* | 250 | - L_WATER - O:P_ WATER | 3 | 1 |
| *PF_within_* | 1000 | Cond:   - MAIZE - SEMNATGRASS - RAPESEED - L_ROAD - L_WATER   Zi:   - RAPSEED - L_ROAD - L_WATER - L_WOOD - O:P_WOOD - O:P_WATER | 1320 | 14 |
| *A_r_* | 1000 | - MAIZE - SEMNATGRASS - L_ROAD - N_POPULATIONS | 11 | 3 |

**Table S10.2:** The table summarizes the outcome of the single best model for *PF_within_* at the 50 m buffer. To test the effects of the landscape metrics on *PF_within_*, generalized linear mixed models (GLMM) with a conditional (cond) part and a zero-inflation (zi) part were used (see Data Analysis section for more details).

|  | Estimate | Standard error | *z*-statistic | *p*-value |
| --- | --- | --- | --- | --- |
| MAIZE_50 (cond) | 0.384 | 0.442 | 0.869 | 0.385 |
| MAIZE_50^2 (cond) | -0.910 | 0.355 | -2.562 | 0.010 |

**Table S10.3:** The table summarizes the outcome of the model averaging procedure for *A_r_* at the 50 m buffer. To test the effects of the landscape metrics on *A_r_*, linear mixed models (LMM) were used (see Data Analysis section for more details).

|  | Estimate | Standard error | *z*-statistic | *p*-value |
| --- | --- | --- | --- | --- |
| L_WATER_50 | 0.129 | 0.039 | 3.253 | 0.001 |
| MAIZE_50 | -0.077 | 0.045 | 1.689 | 0.091 |

**Table S10.4:** The table summarizes the outcome of the model averaging procedure for *PF_within_* at the 250 m buffer. To test the effects of the landscape metrics on *PF_within_*, generalized linear mixed models (GLMM) with a conditional (cond) part and a zero-inflation (zi) part were used (see Data Analysis section for more details).

|  | Estimate | Standard error | *z*-statistic | *p*-value |
| --- | --- | --- | --- | --- |
| L_ROAD_250 (cond) | 0.217 | 0.101 | 2.147 | 0.032 |
| MAIZE_250 (cond) | -0.513 | 0.132 | -3.891 | 0.0001 |
| MAIZE_250^2 (cond) | -0.618 | 0.159 | -3.89 | 0.0001 |
| N. Populations 250 (zi) | 1.189 | 0.425 | 2.794 | 0.005 |

**Table S10.5:** The table summarizes the outcome of the model averaging procedure for *A_r_* at the 250 m buffer. To test the effects of the landscape metrics on *A_r_*, linear mixed models (LMM) were used (see Data Analysis section for more details).

| column name | Estimate | Standard error | *t*-statistic | *p*-value |
| --- | --- | --- | --- | --- |
| L_WATER_250 | -0.058 | 0.045 | -1.268 | 0.209 |
| O:P_WATER_250 | -0.135 | 0.038 | -3.596 | 0.0006 |
| L_WATER_250 X O:P_WATER_250 | -0.152 | 0.048 | -3.141 | 0.003 |

**Table S10.6:** The table summarizes the outcome of the model averaging procedure for *PF_within_* at the 1000 m buffer. To test the effects of the landscape metrics on *PF_within_*, generalized linear mixed models (GLMM) with a conditional (cond) part and a zero-inflation (zi) part were used (see Data Analysis section for more details).

|  | Estimate | Standard error | *z*-statistic | *p*-value |
| --- | --- | --- | --- | --- |
| MAIZE_1000 (cond) | -0.922 | 0.385 | 2.364 | 0.018 |
| MAIZE_1000^2 (cond) | -0.701 | 0.309 | 2.227 | 0.026 |
| RAPESEED_1000 (cond) | -0.444 | 0.244 | 1.795 | 0.073 |
| RAPESEED_1000^2 (cond) | 0.224 | 0.242 | 0.909 | 0.364 |
| SEMNATGRASS_1000 (cond) | -0.128 | 0.239 | 0.524 | 0.600 |
| SEMNATGRASS _1000^2 (cond) | -0.351 | 0.124 | 2.77 | 0.006 |
| L_WATER_1000 (cond) | -0.383 | 0.378 | 0.995 | 0.32 |
| RAPESEED_1000 (zi) | 0.694 | 0.440 | 1.554 | 0.120 |
| L_WATER_1000 (zi) | 1.987 | 0.706 | 2.773 | 0.006 |
| O:P_WATER_1000 (zi) | -1.916 | 1.066 | 1.769 | 0.077 |
| L_WATER_1000 x O:P_WATER_1000 (zi) | -0.183 | 0.599 | 0.300 | 0.764 |
| L_ROAD_1000 (zi) | 0.761 | 0.492 | 1.52 | 0.129 |
| L_WOOD_1000 (zi) | 0.021 | 0.366 | 0.057 | 0.955 |
| O:P_WOOD_1000 (zi) | -0.435 | 0.439 | 0.977 | 0.328 |
| L_ WOOD _1000 x O:P_ WOOD _1000 (zi) | -1.446 | 0.430 | 3.308 | 0.001 |

**Table S10.7:** The table summarizes the outcome of the model averaging procedure for *A_r_* at the 1000 m buffer. To test the effects of the landscape metrics on *A_r_*, linear mixed models (LMM) were used (see Data Analysis section for more details).

|  | Estimate | Standard error | *z*-statistic | *p*-value |
| --- | --- | --- | --- | --- |
| (Intercept) | 2.961 | 0.045 | 64.964 | 0.000 |
| L_ROADS_1000 | -0.078 | 0.042 | 1.816 | 0.069 |
| MAIZE_1000 | -0.027 | 0.046 | 0.578 | 0.563 |
| MAIZE_1000^2 | -0.086 | 0.029 | 2.884 | 0.004 |
| SEMNATGRASS_1000 | 0.032 | 0.036 | 0.851 | 0.395 |

# Supplement 11: Models used for figures in the main text

**Table S11:** To illustrate the effects of the landscape metrics, we plotted their effects from the models in which they showed the strongest effects. The table below lists the models on which the specific figures are based, with standardized regression coefficients shown in brackets. To test the effects of the landscape metrics on *PF_within_*, generalized linear mixed models (GLMM) with a conditional (cond) part and a zero-inflation (zi) part were used. To test the effects of the landscape metrics on *A_r_*, linear mixed models (LMM) were used.

| Figure ID | Model |
| --- | --- |
| Figure 5a | *PF_within_* ~  cond: MAIZE_50 [0.38] + MAIZE_50^2 [-0.91] |
| Figure 5b | *PF_within_* ~  cond: MAIZE_250 [-0.51] + MAIZE_250^2 [-0.62] + L_ROAD [0.22]  zi: N_ populations_250 [1.19] |
| Figure 5c | *PF_within_* ~  cond: MAIZE_1000 [1.25] + MAIZE_1000^2 [-0.82] + L_WATER_1000 [-0.33] + RAPESEED_1000 [-0.62]  zi: L_WATER_1000 [2.09] + WATER_1000_O:P [-1.69] + RAPESEED_1000 [0.55] + L_WATER_1000 x WATER_1000_O:P [-0.02] |
| Figure 5d | *A_r_* ~  MAIZE_1000[-0.06] + MAIZE_1000^2 [-0.1] |
| Figure 5e | *PF_within_* ~  cond: MAIZE_1000 [-0.69] + MAIZE_1000^2 [-0.61] + SEMNATGRASS_1000[-0.15] + SEMNATGRASS^2_1000 [-0.36]  zi : L_WOOD_1000 [0.02] + WOOD_1000_O:P [-0.24] + L_WOOD_1000 x WOOD_1000_O:P [-1.32] |
| Figure 6a | *A_r_* ~  L_WATER_250 [-0.06] + L_WATER_O:P [-0.14] + L_WATER_250: L_WATER_O:P [-0.15] |
| Figure 6b | *PF_within_* ~  cond: MAIZE_1000 [-0.65] + MAIZE^2_1000 [-0.61] +  SEMNATGRASS _1000 [-0.14] + SEMNATGRASS_1000^2 [-0.36]  zi L_ROAD_1000 [0.62] + L_WOOD_1000 [0]+ WOOD_1000_O:P [-0.69] + L_WOOD_1000: WOOD_1000_O:P [-1.61] |
| Figure 7a | *PF_within_* ~  cond: L_ROAD [0.22]_250 + MAIZE_250 [-0.51]+ MAIZE_250^2 [-0.62] +  zi: N_populations_250 [1.19] |
