# Supplementary material for: Greater pollen-mediated gene flow among than within populations of a bumblebee-pollinated forest herb despite habitat fragmentation
Source: Mov Ecol. 2026 May 30;14:36. doi: 10.1186/s40462-026-00664-8 (PMC13224658; doi:10.1186/s40462-026-00664-8)
Supplement: Supplementary file 3 — Supplementary Material 3 [file 40462_2026_664_MOESM3_ESM.pdf]

Supplement: Greater pollen-mediated gene flow among than within populations of a bumblebee-pollinated forest herb despite habitat fragmentation

Supplement 1: Shoot clusters of *Polygonatum multiflorum* and their bumblebee pollinators............. 2
Supplement 2: Overview over included samples.. 4
Supplement 3: Stratification and germination... 14
Supplement 4: Genotyping, quality and assessment of clonality 15
Supplement 5: Information on quality of paternity analysis 20
Supplement 6: Landscape metrics 21
Supplement 7: Distribution of pollen flow measures 23
Supplement 8: Model outcome H2 34
Supplement 9: Collinearity among included variables... 36
Supplement 10: Outcome of Model averaging for H3 and H4... 39
Supplement 11: Models used for figures in the main text.. 43

Supplement 1: Shoot clusters of *Polygonatum multiflorum* and their bumblebee pollinators

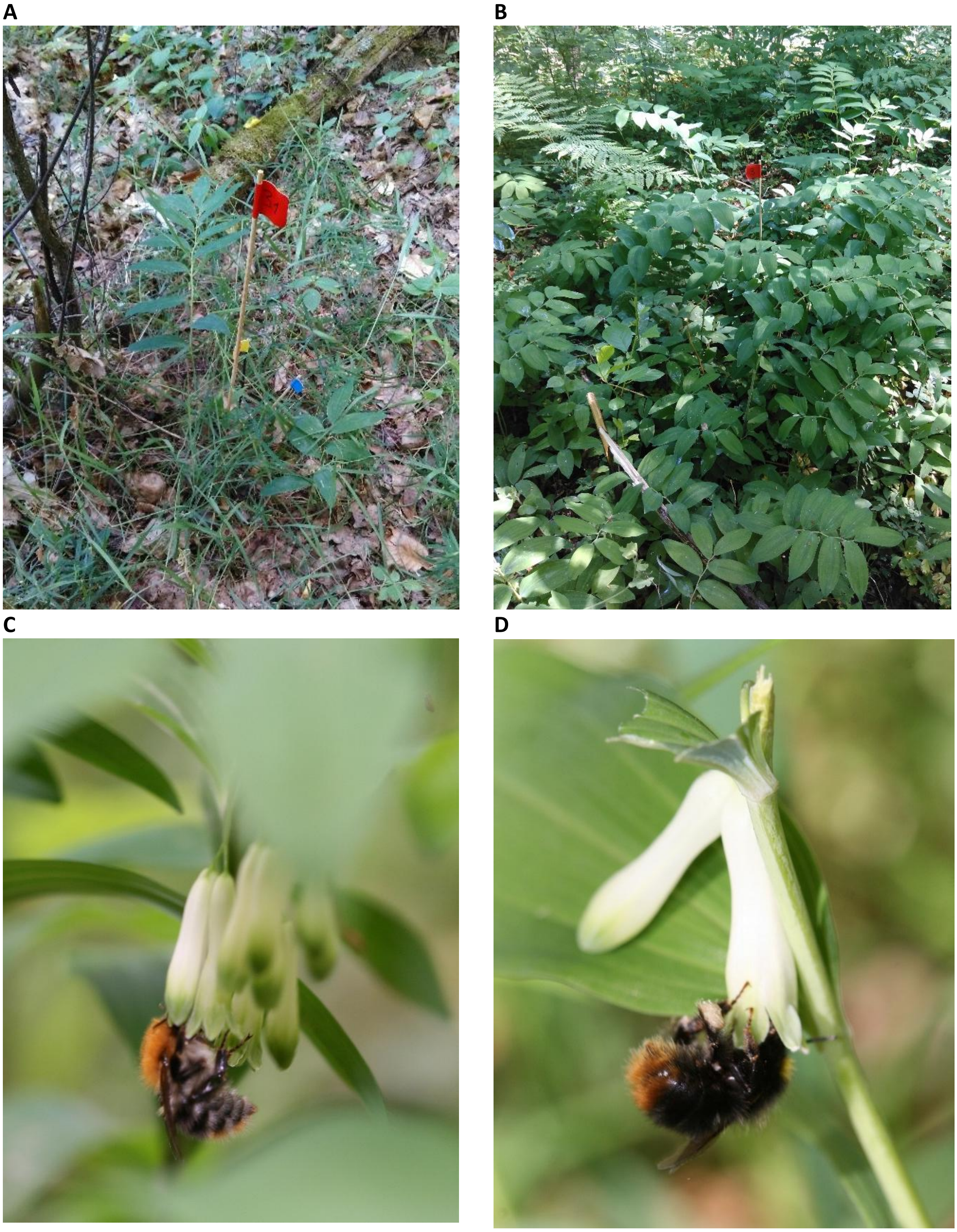

Figure S1: Pictures A and B: Shoot clusters of *P. multiflorum* with only a few flowering shoots (A) and multiple shoots (B); pictures C and D: Flower visits of *Bombus pascuorum* (C) and *Bombus pratorum* (D).

Table S1: During 26 hours of flower observations, we recorded between one and four different bumblebee species per forest patch visiting *P. multiflorum*, out of a total of five species. In addition, we observed other wild bee visitors from the genus *Anthophora*. The table shows the total observation time in 2018 and 2019, as well as the number of individuals per species per forest patch. Details of the observation procedure are provided in Naaf et *al*. (2021), Supplement S1.

**Forest patch ID**	**Time observed [hours:min]**	***B. pascuorum***	***B. pratorum***	***B. hortorum***	***B. lapidarius***	***B. hypnorum***	***Bombus species***	***Anthophora spp***
**F01**	4:30	7	6	0	1	0	3	0
**F04**	2:00	4	0	0	0	0	1	0
**F06**	1:30	3	2	0	0	0	2	0
**F07**	4:00	16	10	0	0	0	2	0
**F08**	3:00	7	4	0	0	0	2	0
**F10**	3:30	5	11	0	0	1	3	2
**F45**	1:30	1	0	0	0	0	1	0
**F48**	3:00	6	13	2	1	0	4	2
**F51**	3:00	4	0	0	0	0	1	0

Reference

Naaf, T., Feigs, J. T., Huang, S., Brunet, J., Cousins, S. A. O., Decocq, G. Sensitivity to habitat fragmentation across European landscapes in three temperate forest herbs. *Landscape Ecology*. 2021; *36*(10), 2831-2848. https://doi.org/10.1007/s10980-021-01292-w

Supplement 2: Overview over included samples

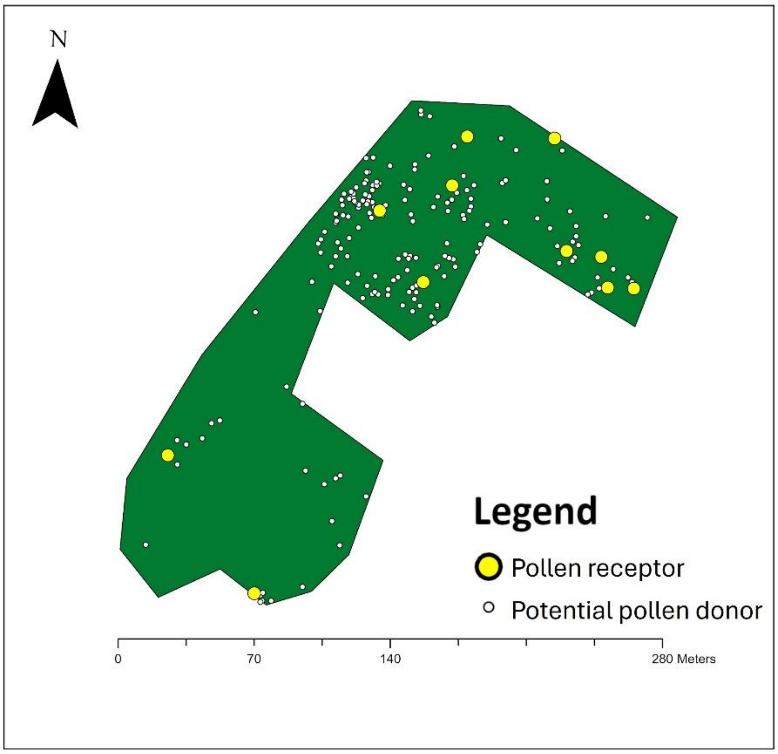

Figure S2a: This figure shows the sampling in forest patch F01. Yellow dots represent the plants selected as pollen receptors, while grey dots indicate all potential pollen donors.

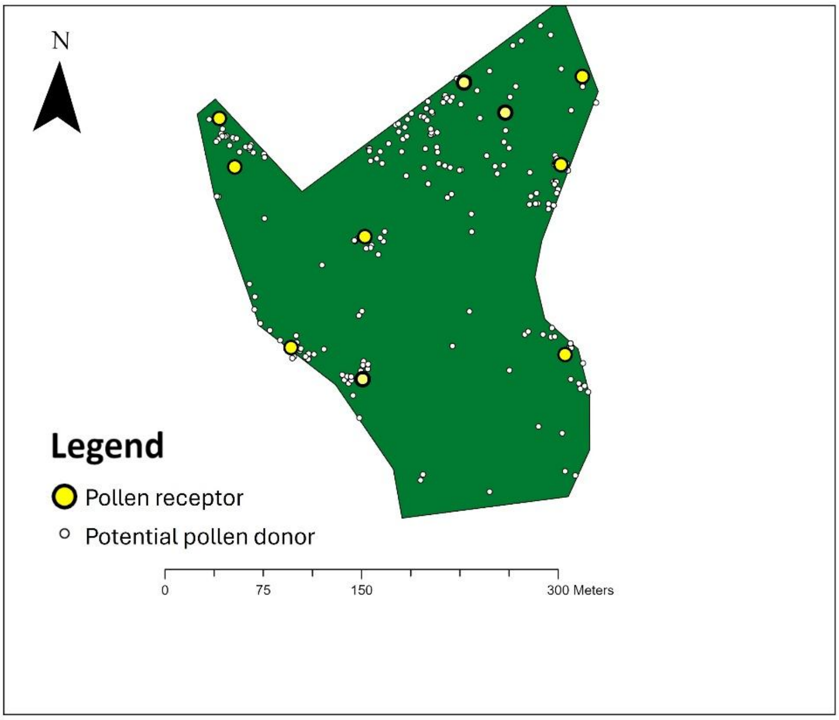

Figure S2b: This figure shows the sampling in forest patch F04. Yellow dots represent the plants selected as pollen receptors, while grey dots indicate all potential pollen donors.

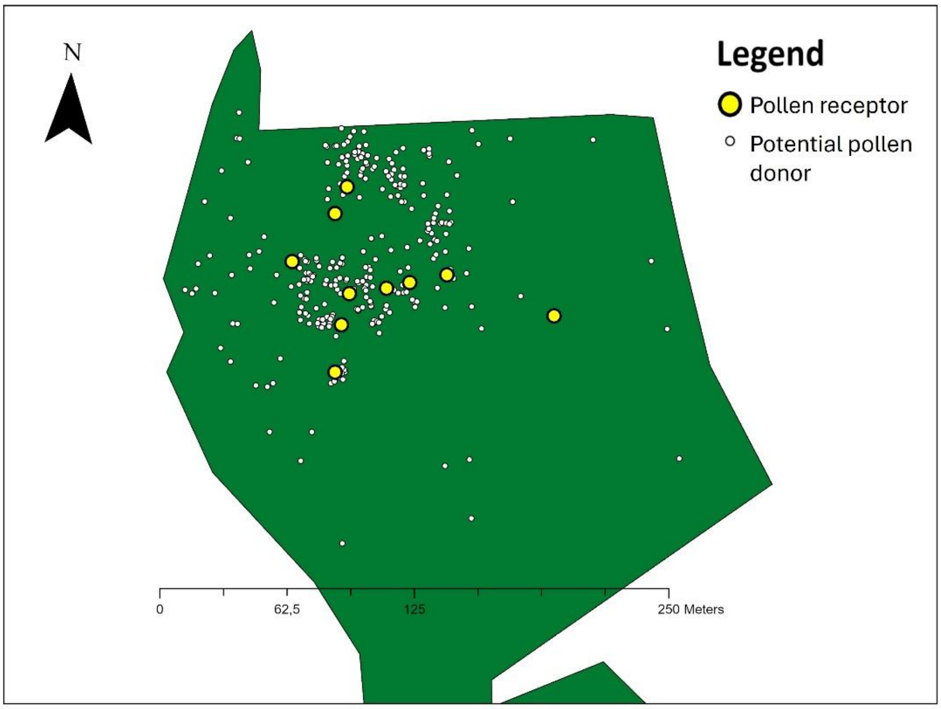

Figure S2c: This figure shows the sampling in forest patch F06. Yellow dots represent the plants selected as pollen receptors, while grey dots indicate all potential pollen donors.

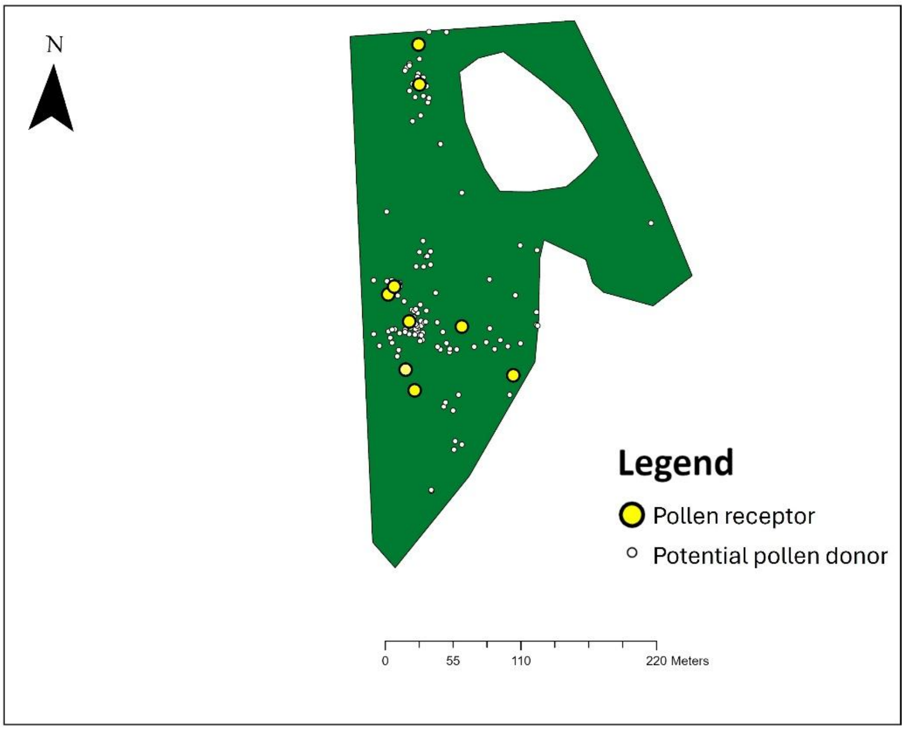

Figure S2d: This figure shows the sampling in forest patch F07. Yellow dots represent the plants selected as pollen receptors, while grey dots indicate all potential pollen donors.

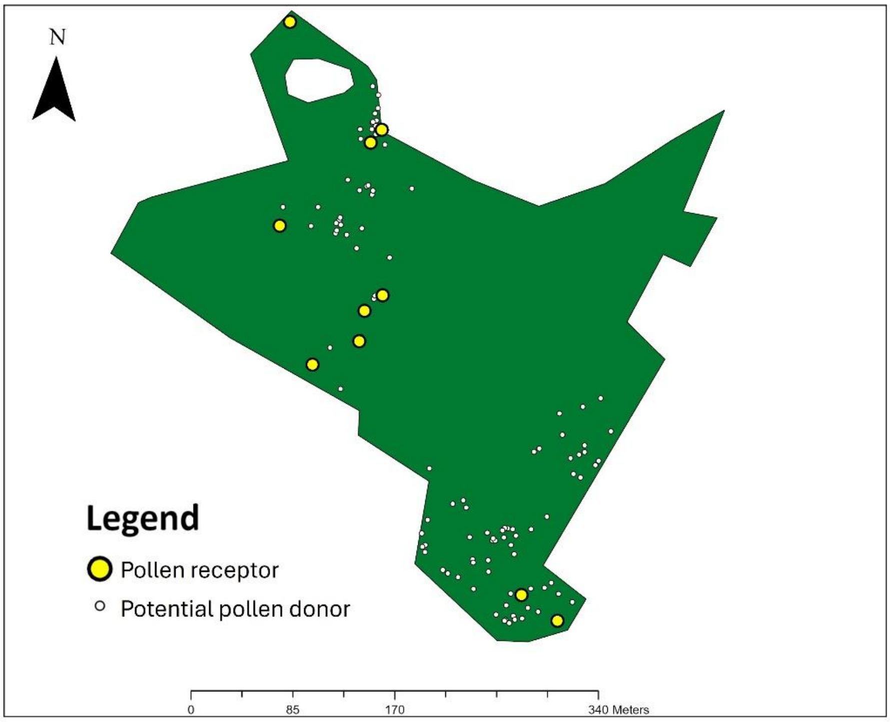

Figure S2e: This figure shows the sampling in forest patch F08. Yellow dots represent the plants selected as pollen receptors, while grey dots indicate all potential pollen donors.

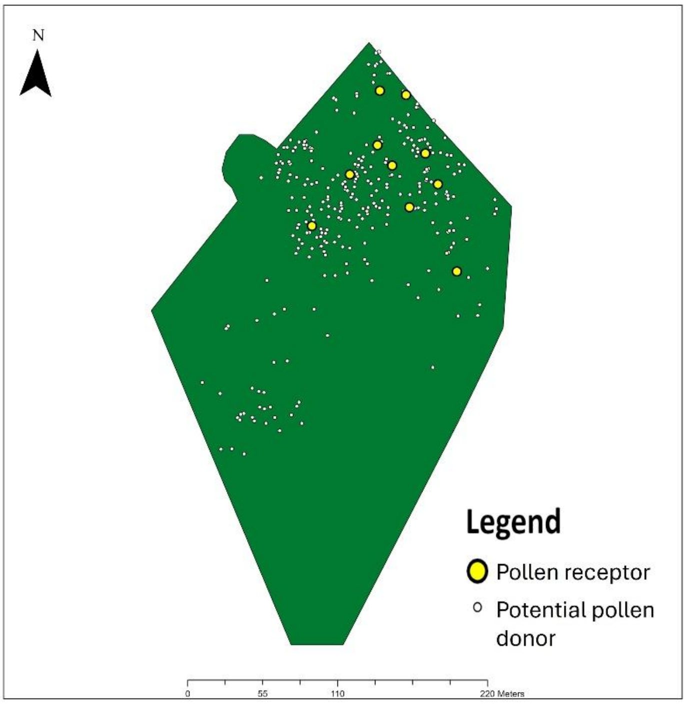

Figure S2f: This figure shows the sampling in forest patch F10. Yellow dots represent the plants selected as pollen receptors, while grey dots indicate all potential pollen donors.

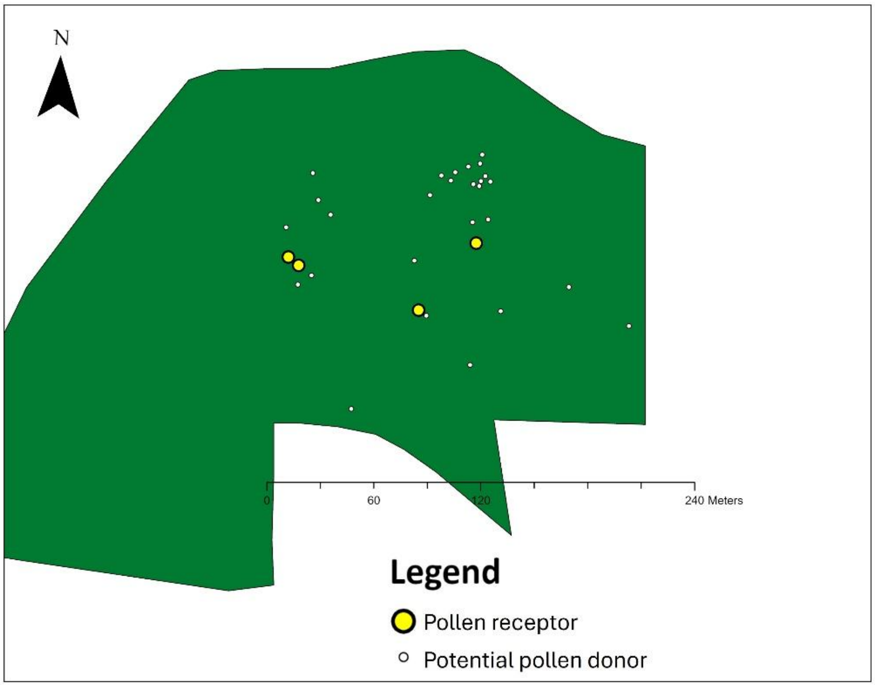

Figure S2g: This figure shows the sampling in forest patch F45. Yellow dots represent the plants selected as pollen receptors, while grey dots indicate all potential pollen donors.

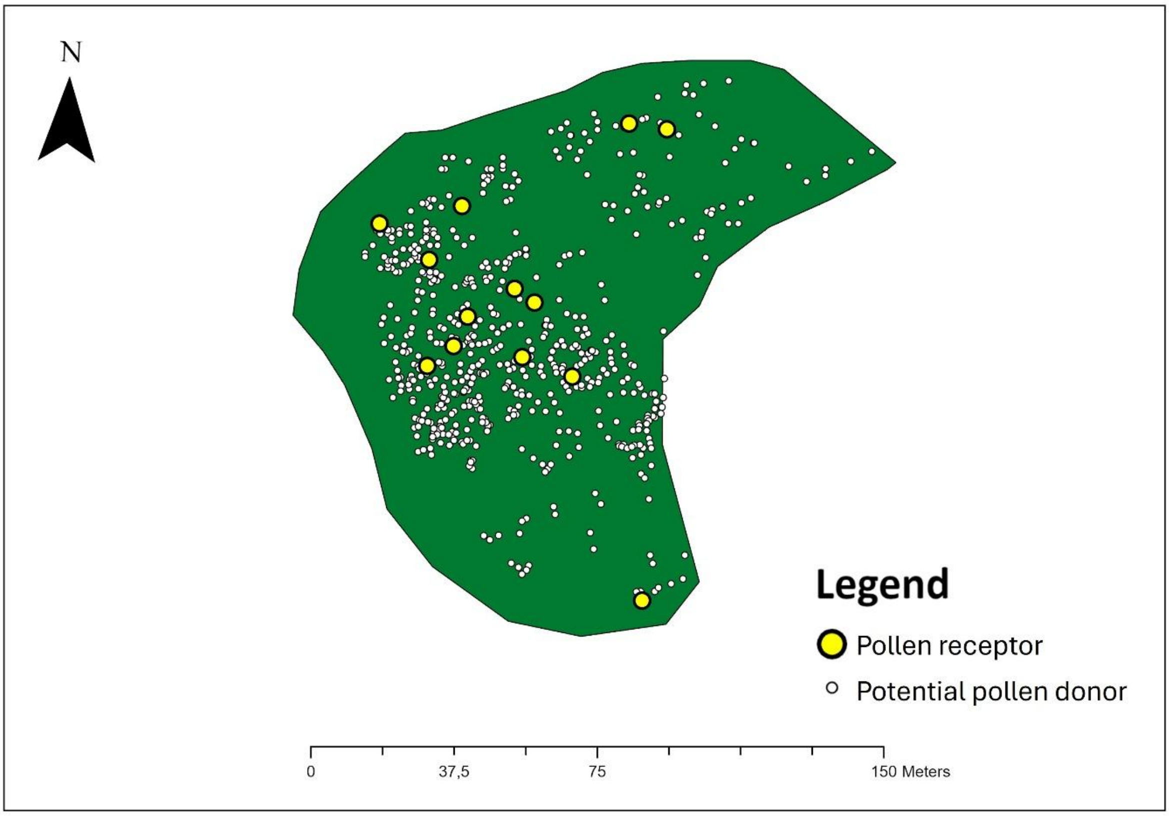

Figure S2h: This figure shows the sampling in forest patch F48. Yellow dots represent the plants selected as pollen receptors, while grey dots indicate all potential pollen donors.

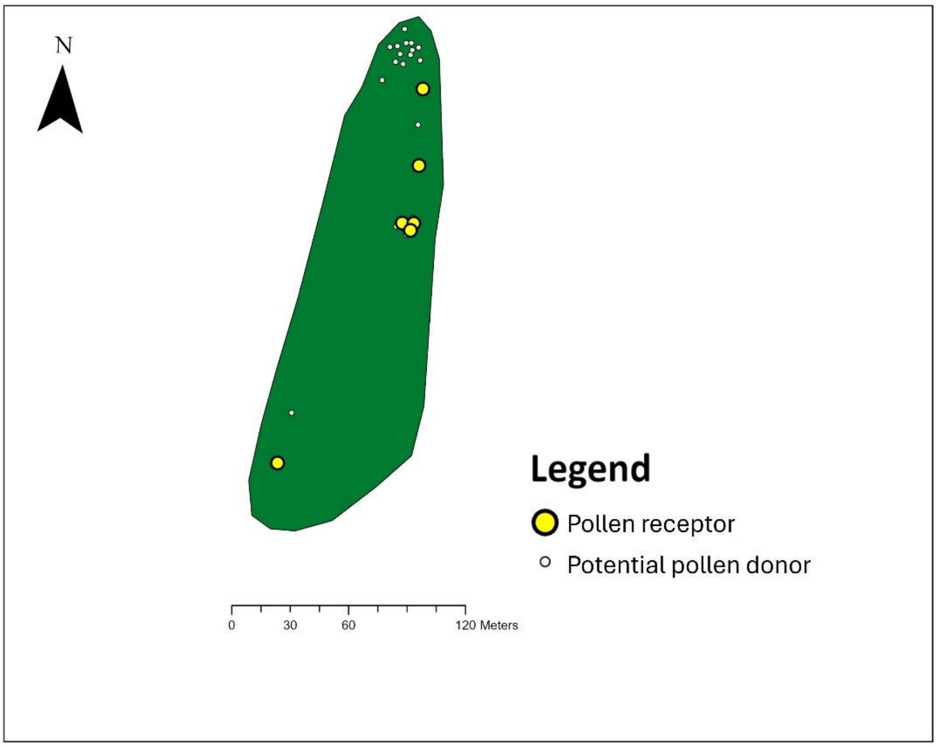

Figure S2i: This figure shows the sampling in forest patch F10. Yellow dots represent the plants selected as pollen receptors, while grey dots indicate all potential pollen donors.

Table S2.1 Column descriptions: explaining the content of each column of "Table S2.1.xlsx", provides the exact number of samples involved at each step of the analysis. For clarity, we divided the study process into four steps, organized as follows: **Step 1:** Sampling and germination; **Step 2:** Quality and clonality checks, **Step 3:** Paternity analysis, **Step 4:** Samples used for testing H1, H3 and H4.

Column name	Description
**Step 1: Sampling**	This step is based on the dataset Table S2.3 Al_pollen_donors.xlsx
**n sampled shoot cluster**	The total number of shoots within each forest patch. All sampled in 2018. For each cluster, the number of shoots was documented. See Figure S2 for the spatial distribution of the shoot clusters.
**n shoot cluster = 1 shoot**	Number of all single shoots from each forest patch that were sampled in spring 2018.
**n shoot cluster 2-9 shoots**	Number of all shoot clusters with two to nine shoots from each forest patch sampled in spring 2018.
**n shoot cluster 10-19 shoots**	Number of all shoot clusters with ten to 19 shoots from each forest patch sampled in spring 2018.
**n shoot cluster >= 20 shoots**	Number of all shoot clusters with more than 19 shoots sampled from each forest patch sampled in spring 2018.
**Proportion of shoot cluster = 1 shoot**	Proportion of single shoots among the n sampled shoot clusters per forest patch.
**Proportion of shoot cluster 2-9 shoots**	Proportion of clusters with two to nine shoots among the n sampled shoot clusters per forest patch.
**Proportion of shoot cluster 9-19 shoots**	Proportion of shoot clusters with ten to 19 shoots among the n sampled shoot clusters per forest patch.
**Proportion of shoot cluster >= 20 shoots**	Proportion of shoot clusters with more than 19 shoots among the n sampled shoot clusters per forest patch.
**Step 2 Quality and clonality checks**	This step is based on the dataset Table S4.2 Allele_table.xlsx
**n successfully genotyped samples**	Number of leaf and rhizome samples successfully genotyped after DNA isolation.

**n successfully genotyped leaf samples as potential pollen donors**	Number of leaf samples, successfully genotyped after DNA isolation, that were considered as potential pollen donors.
**n successfully genotyped leaf samples for within shoot clusters**	Number of leaf samples, successfully genotyped after DNA isolation, that were used for the clonality test within the shoot clusters.
**n successfully genotyped leaf samples as pollen receptors**	Number of leaf samples, successfully genotyped after DNA isolation, that were considered as pollen receptors.
**n successfully genotyped rhizome samples genotyped as offspring**	Number of rhizome samples, successfully genotyped after DNA isolation, that were considered as pollen receptors.
**n samples removed from n successfully genotyped samples because of missing values <1**	Number of leaf and rhizome samples that were removed because of too many missing values.
**n samples included in clonality test among shoot clusters**	Number of leaf and rhizome samples included in the test of clonality among shoot clusters.
**n distinct MLGs**	Number of distinct multilocus genotypes identified in the clonality test among shoot clusters.
**n identified clones among shoot clusters**	Number of clones identified in the clonality test among shoot clusters.
**n shoot clusters included in clonality test within shoot clusters**	Number of shoot clusters that were included for the clonality test within shoot clusters (see Table S4.2 and S4.3 for details).
**n genotyped leaf samples included in clonality test within shoot clusters**	Number of all multilocus genotypes included in the clonality test within shoot clusters (see Table S4.2 and S4.3 for details).
**n distinct identified MLGs in clonality test within shoot clusters**	Number of distinct multilocus genotypes included in the clonality test within shoot clusters (see Table S4.2 and S4.3 for details).
**n additional genotypes from clonality test within shoot clusters**	Number of additional distinct multilocus genotypes identified through the within shoot cluster clonality test.
**n all samples after Step 2**	Number of remaining genotypes after the checks of step 2 (Table S2.3)
**Step 3 Paternity analysis**	

**n MLGs included in paternity analysis**	Total number of all multilocus genotypes that were included in the paternity analysis.
**n potential pollen donor MLGs**	Number of all multilocus genotypes that were included in the paternity analysis as potential pollen donors (including pollen receptors).
**n pollen receptors MLGs**	Number of all multilocus genotypes that were included in the paternity analysis as pollen receptors.
**n offspring MLGs**	Number of all multilocus genotypes that were included in the paternity analysis as offspring with known pollen receptors.
**Step 4 Data analysis**	
**n pollen receptors included in testing H1, H3, H4**	Number of pollen receptors with more than five offspring, for which PF_{within} and allelic richness were calculated.
**n shoot clusters included in testing H2**	Number of included shoot clusters with known shoot numbers.

Samples removed in Step 2: Quality and clonality checks

Table S2.2: The table summarizes how many potential pollen donors, pollen receptors and offspring samples were excluded due too many missing values, duplicate occurrences of identical multi-locus genotypes, or more than two mismatches between the genotypes of the pollen receptor and the offspring.

Forest Patch ID	Missing value > 1	Clonality test	Mismatches between pollen receptor and offspring > 2
F01	5 potential pollen donors	18 potential pollen donors	none
F04	none	46 potential pollen donors<br>1 offspring	none
F06	2 potential pollen donors<br>1 offspring	55 potential pollen donors	1 offspring
F07	1 offspring	16 potential pollen donors	none
F08	none	8 potential pollen donors<br>1 offspring	none
F10	1 potential pollen donor<br>2 offspring	40 potential pollen donors<br>1 offspring	none
F45	1 offspring	4 potential pollen donors<br>2 offspring	none
F48	none	73 potential pollen donors<br>1 offspring	none
F51	1 offspring	5 p potential pollen donors<br>1 pollen receptor<br>5 offspring	1 offspring
Total	8 potential pollen donors<br>6 offspring	265 potential pollen donors<br>11 offspring<br>1 pollen receptor	2 offspring

Table S2.3 Column descriptions: The attached Excel file, "Table S2.3 All_pollen_donors.xlsx", provides information for each sampled shoot cluster.

Column name	Description
Shoot_Cluster_ID	Unique identifier for each shoot cluster
Forest_Patch_ID	Unique identifier of the forest patch
No_shoots	Number of shoots in the shoot cluster counted during the May campaign 2018
No_sampled	Number of shoots in the shoot cluster that were sampled
Date_Sampling	Date when the leaf material was collected
Longi_WGS84 and Lati_WGS84	The GPS coordinates of the shoot cluster (WGS84)

Supplement 3: Stratification and germination

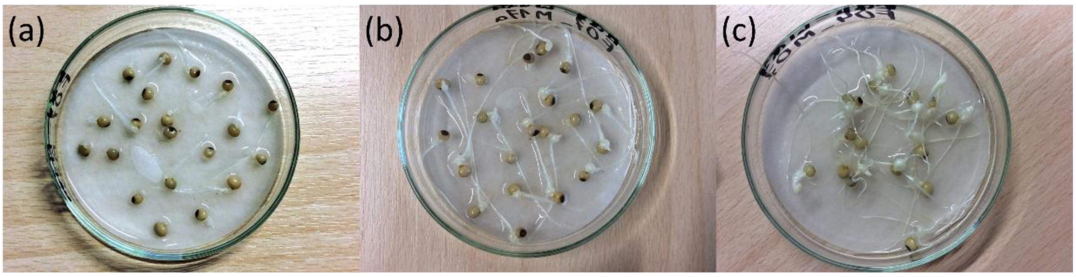

Figure S3: Photographs after stratification: (a) 3.5 weeks, (b) 5 weeks, and (c) 10 weeks after start of germination.

Protocol:

1. We extracted seeds from the fruits and thoroughly cleaned them from the fruit pulp.
2. We included a total of 1442 seeds in the stratification and germination procedure. They originated from 91 different pollen receptors (6 to 14 pollen receptors per forest patch; 1 to 46 seeds per pollen receptor; mean: 17)
3. We placed the 20 seeds on wet filter paper (soaked with distilled water) on Petri dishes with a diameter of 88 mm. The Petri dishes were covered with a lid to reduce evaporation.
4. We exposed the seeds to a cold-wet stratification, i.e., we placed the Petri dishes in a climate chamber at 5°C for six weeks and kept the filter paper wet during this time.
5. After six weeks, the Petri dishes were kept in a closed box at room temperature (around 20°C) for another seven weeks. The filter paper was kept moist during this time.
6. We obtained a total number of 1006 seedlings, which indicates a germination rate of 0.70.

Supplement 4: Genotyping, quality and assessment of clonality

Table S4.1: The table describes the seven different microsatellite primers used in this study. It reports the size ranges in base pairs [bp], the number of alleles and genotypes, the amount of missing values and pollen receptor-offspring mismatches, as well as the genotyping error rates based on 10 % repetition as well as on pollen receptor-offspring mismatches.

**Primer name**	**Range [bp]**	**Alleles [N]**	**Genotypes [N]**	**Percentage of missing value [%]**	**Error rate based on 10% repetition [%]**	**Receptor-offspring mismatch or NA [N]**	**Error rate based on Receptor-offspring mismatches [%]**
**Pmu024**[1]	125-129	2	2	0	0.61	0	0
**Pmu091**[1]	243-276	12	53	0.19	0.31	2	0.21
**Pc33**[2]	233-277	22	164	0.34	0.31	8	0.84
**Pc25**[2]	215-263	15	67	0.49	0	4	0.42
**Pmu008**[1]	187-190	2	4	0.68	1.22	0	0
**Pmu373**[1]	100-106	2	4	0.15	0.92	0	0
**Pt09**[2]	126-216	42	448	0.52	1.22	9	0.95

[1] Newly developed for this study

[2] Developed for *P. cyrtonema* by Cheng et al. (2010) and for *P. filipes* by Liu et al. (2010).

References

Cheng WJ, Liu TT, Wu HL, Zhou SB, Xuan SQ, Zhu GP. Isolation and characterization of twelve polymorphic microsatellite loci in *Polygonatum cyrtonema* and cross-species amplification. Conserv Genet Resour. 2010; 2:105-107. https://doi.org/10.1007/s12686-010-9218-1

Liu TT, Cheng WJ, Zhou SB, Shao JW, Wu HL, Zhu GP. Eleven polymorphic microsatellite loci in Polygonatum filipes and cross-amplification in other congeneric species. Conserv Genet Resour. 2010; 2:77-79. https://doi.org/10.1007/s12686-010-9179-4

Table S4.2 Column descriptions: The attached Excel file, "Table S4.2 Allele table", contains two sheets: "Alleles", which lists allele data for each sample at specific loci, and "Sample_info", which provides additional sample information.

Column name	Description
**Both sheets**	
**Sample ID**	Unique identifier of the sample
**Sheet: „Alleles“**	
**Rows B-O**	Alleles at the following loci: Pmu024, Pmu091, Pc33, Pc25, Pmu008, Pmu373, Pt09 (See: Table S4,1). As we used codominant markers, two alleles are recorded per locus and sample, indicated by "_1" and "_2". Missing values are coded as "-999".
**Sheet "Sample_info"**	
**Pollen_receptor_ID**	Identifier of the pollen receptor to which the sample belonged. This information is only relevant for samples with the purpose "Offspring" or "Pollen_receptor".
**Shoot_cluster_ID**	Identifier of the shoot cluster from which the sample was taken.
**Forest_patch**	Identifier of the forest patch from which the sample was taken.
**Purpose**	Original purpose for which the sample was collected: "pollen receptor", "pollen donor", "offspring", and "Clone test" = (sampled to test within-shoot-cluster clonality).
**For_clone_check**	Indicated samples included in the test of within shoot cluster clonality, including samples originally classified as "Pollen_donor".
**Year**	Year when the sample was collected.

Clonality among shoot clusters

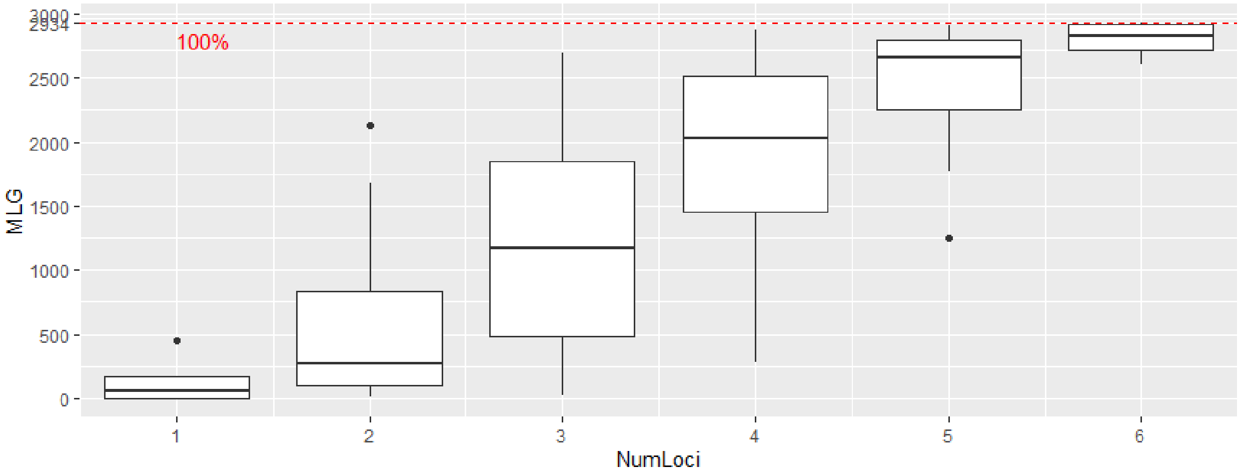

Figure S4.1: Multi locus genotype (MLG) accumulation curve based on 200 subsamples for each number of loci.

Clonality within shoot clusters

Table S4.3: Detailed overview of the shoots analyzed in the comparisons of shoot clusters of different sizes. Reported are numbers of flowering shoots per cluster, the number of collected samples, the number of samples that were genotyped, and the number of identified multi locus genotypes (MLGs).

Polygonatum patch	Flowering shoots	Samples collected in the field	Genotyped samples	MLG
F01_P22	2	2	2	1
F04_P37	2	2	2	1
F04c_P09	2	2	2	2
F06b_P37	2	2	2	1
F10b_P40	2	2	2	2
F04a_P16	3	3	3	2
F06_P03	3	3	3	1
F48d_P31	3	3	3	3
F01a_P39	4	2	2	1
F04b_P25	4	2	2	2
F06e_P42	4	2	2	1
F10a_P22	4	2	2	1
F10c_P37	4	2	2	1
F04_P05	6	3	3	1
F10c_P04	6	3	3	2
F48b_P17	6	2	2	1
F48j_P24	6	3	3	1
F48k_P57	6	5	5	5
F07a_P55	7	2	2	2
F10_P35	7	2	2	1
F48f_P22	7	2	2	1

F10c_P45	8	2	2	1
F04c_P02	9	5	5	3
F10b_P52	10	2	2	1
F48_P58	10	3	3	2
F48a_P34a	10	2	2	2
F07b_P17	11	2	2	1
F48c_P08	11	3	3	2
F01a_P30	12	5	5	3
F06a_P43	12	2	2	1
F10d_P13	12	2	2	1
F48d_P04	12	3	3	2
F01a_P53	14	5	5	3
F04c_P47a	15	8	8	4
F08a_P19	15	2	2	1
F48e_P30	16	3	3	2
F48i_P25	16	2	2	2
F10_P51	17	3	3	2
F10b_P49b	17	2	2	1
F04c_P41	18	3	3	2
F06e_P48a	18	2	2	1
F06d_P19	20	5	5	1
F48_P42b	21	3	3	2
F01b_P10	22	5	5	1
F10_P43	22	2	2	2
F06e_P01	24	8	8	1
F10a_P46	25	3	3	2
F48c_P43	27	3	3	2
F10b_P04	33	5	5	2
F48e_P20	36	5	4	3
F06b_P39	37	8	8	3
F10c_P13	46	5	4	1
F04d_P32	51	8	8	1
F10c_P44	51	5	5	4
F48e_P11	67	8	8	7
F48k_P32	69	10	10	6
F06e_P38	103	13	11	7
F48i_P17	119	16	16	14
F07_P03b	190	25	25	17
Total	1316	249	245	144

Table S4.4: The table summarizes how many shoot clusters per size class were included in the assessment of within-shoot-cluster clonality and the proportion of them that were monoclonal.

	**Number of included shoot clusters**	**Monoclonal shoot clusters**
**All**	59	26 (44%)
**≤ 5 flowering shoots**	13	8 (62%)
**5-20 flowering shoots**	28	13 (46%)
**> 20 flowering shoots**	18	5 (28%)

With a higher number of sampled shoots (larger shoot clusters) the percentage of monoclonal shoot clusters is decreasing.

Supplement 5: Information on quality of paternity analysis

Table S5.1: The table shows the reproducibility of the pollen-donor-assignment procedure across independent runs. It indicates how many pollen donor-offspring pairs were consistently assigned in n out of five total runs. In cases where different pollen donors were assigned to the same offspring, assignments were not considered reproducible unless one donor appeared at least in one run more than the other(s), in such cases the dominant donor was considered as the assigned pollen donor. The mean assignment probabilities for pollen donor-offspring pairs, as reported by COLONY 2 are summarized in the rightmost column across different numbers of seeds for all pairs identified in multiple runs.

**Forest Patch**		**Number of runs**				**Differing pollen donors**	**Total**	**Total of reliable assigned offspring**	**Mean, minimum and maximum probability**
	1	2	3	4	5				
F01				**1**	**8**	**0**	**9**	**9**	0.82 (range: 0.81-0.89)
F04	**4**	**2**		**5**	**18**	**0**	**29**	**25**	0.94 (range: 0.74-1)
F06	**2**	**12**	**3**	**14**	**3**	**0**	**34**	**32**	0.87 (range: 0.42-1)
F07	**17**	**7**	**5**		**4**	**3**	**33**	**16**	0.95 (range: 0.88-0.99)
F08	**3**	**15**	**1**		**23**	**0**	**42**	**39**	0.82 (range: 0.59-1)
F10	**1**		**3**	**23**	**9**	**1**	**36**	**35**	0.84 (range: 0.7-1)
F48					**12**	**0**	**12**	**12**	1 (range: 1-1)
F51	**1**			**1**	**5**	**0**	**7**	**6**	1 (range: 1-1
Total	**28**	**36**	**12**	**44**	**82**	**4**	**202**	**174**	

Supplement 6: Landscape metrics

Table S6.1: Landscape metrics used to quantify the landscape composition in buffers with radii of 50 m, 250 m, and 1000 m around pollen receptor plants.

Area-based metrics	Buffer radius [m]	Median, minimum and maximum percentage cover [%] of buffer
Deciduous Forest	50	76 (range: 31.5-100)
Deciduous Forest	250	26.7 (range: 9-41.4)
Deciduous Forest	1000	9.5 (range: 5.7-16.8)
SEMNATGRASS	50	0
SEMNATGRASS	250	0
SEMNATGRASS	1000	0.4 (range: 0-1.3)
RAPESEED	50	0
RAPESEED	250	0
RAPESEED	1000	0.9 (range: 0-21.4)
MAIZE	50	0 (range: 0-73)
MAIZE	250	7.8 (range: 0-71.4)
MAIZE	1000	22.3 (range: 5-47.5)
**Linear landscape elements**		**Median, minimum and maximum length in meters [m]**
L_ROAD	50	0 (range: 0-162)
L_ROAD	250	317 (range: 0-1028)
L_ROAD	1000	5466 (range: 3649-9567)
L_WATER	50	0 (range: 0-153)
L_WATER	250	547 (range: 0-976)
L_WATER	1000	4995 (range: 694-8459)
L_WOOD	50	0
L_WOOD	250	477 (range: 0-2100)
L_WOOD	1000	10140 (range: 2276-19455)

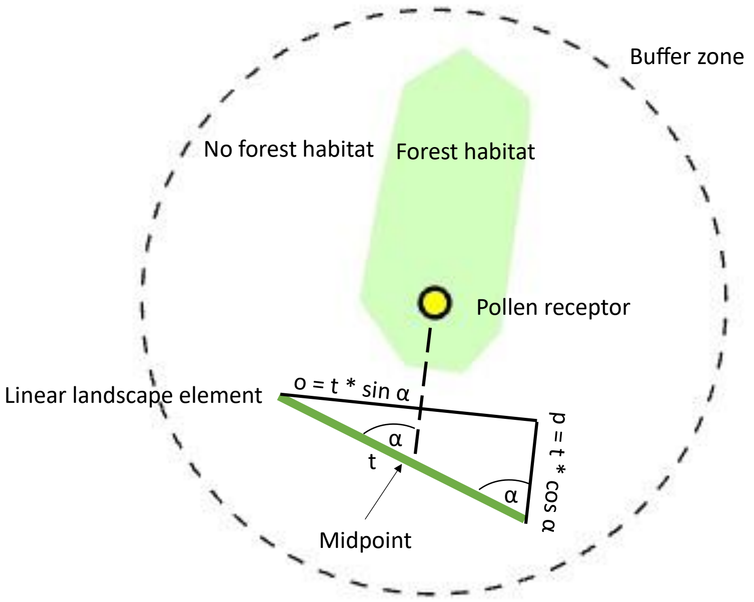

Figure S6.1: Schematic visualization of the calculation of the parallel (p) and orthogonal (o) length component of each specific linear landscape element.

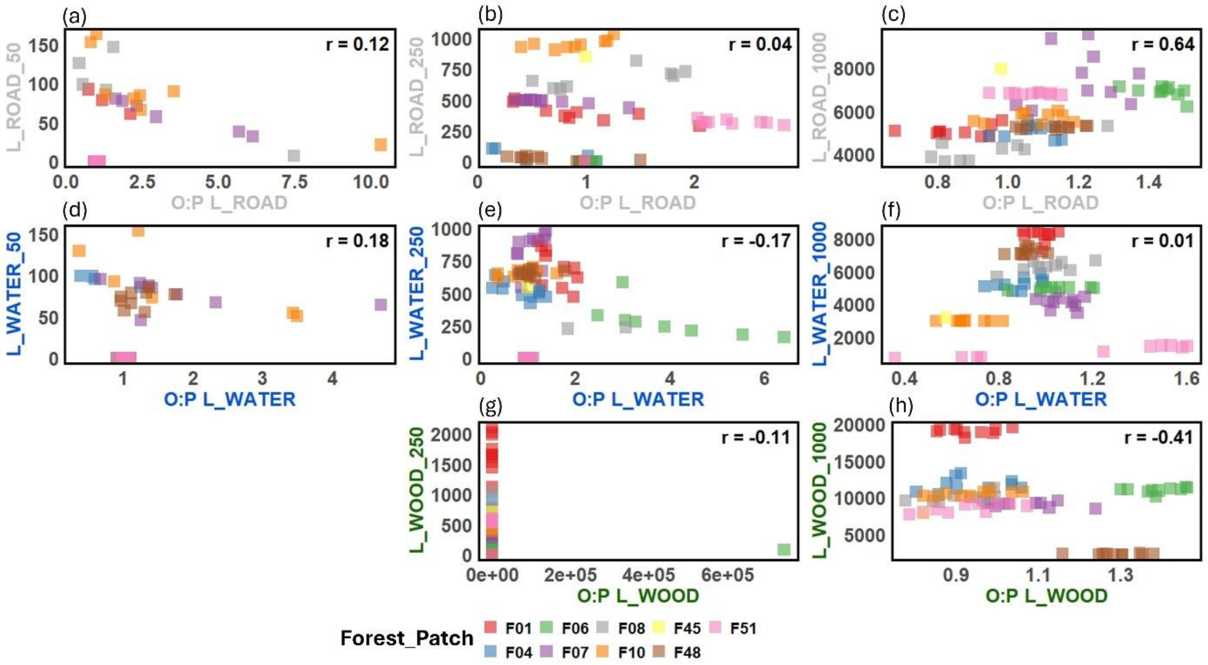

Figure S6.2: Distribution of the length of linear landscape elements and their orthogonal-parallel ratio at three different buffer radii. Colors indicate the forest patch from which the pollen receptor originates.

Supplement 7: Distribution of pollen flow measures

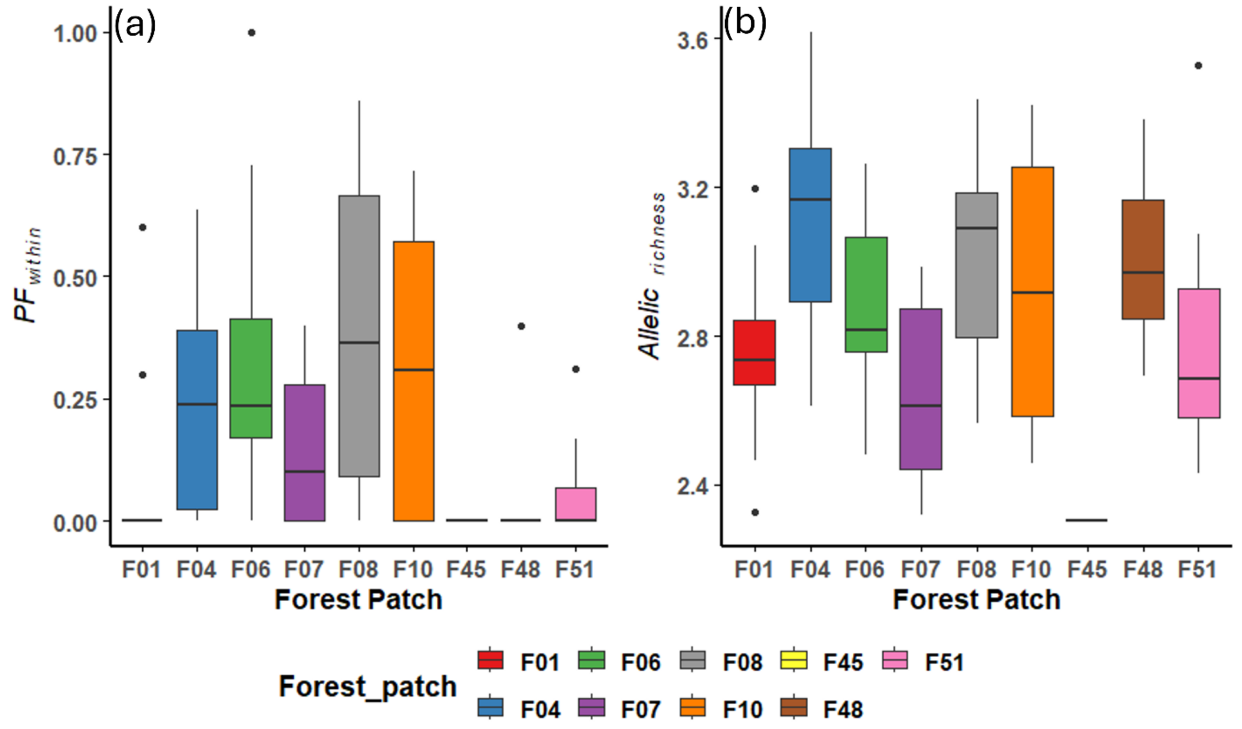

Figure S7.1: The figure shows the distribution of (a) PF_{within} and (b) A_r per pollen receptor among forest patches. Colors indicate the forest patch from which the pollen receptor originates.

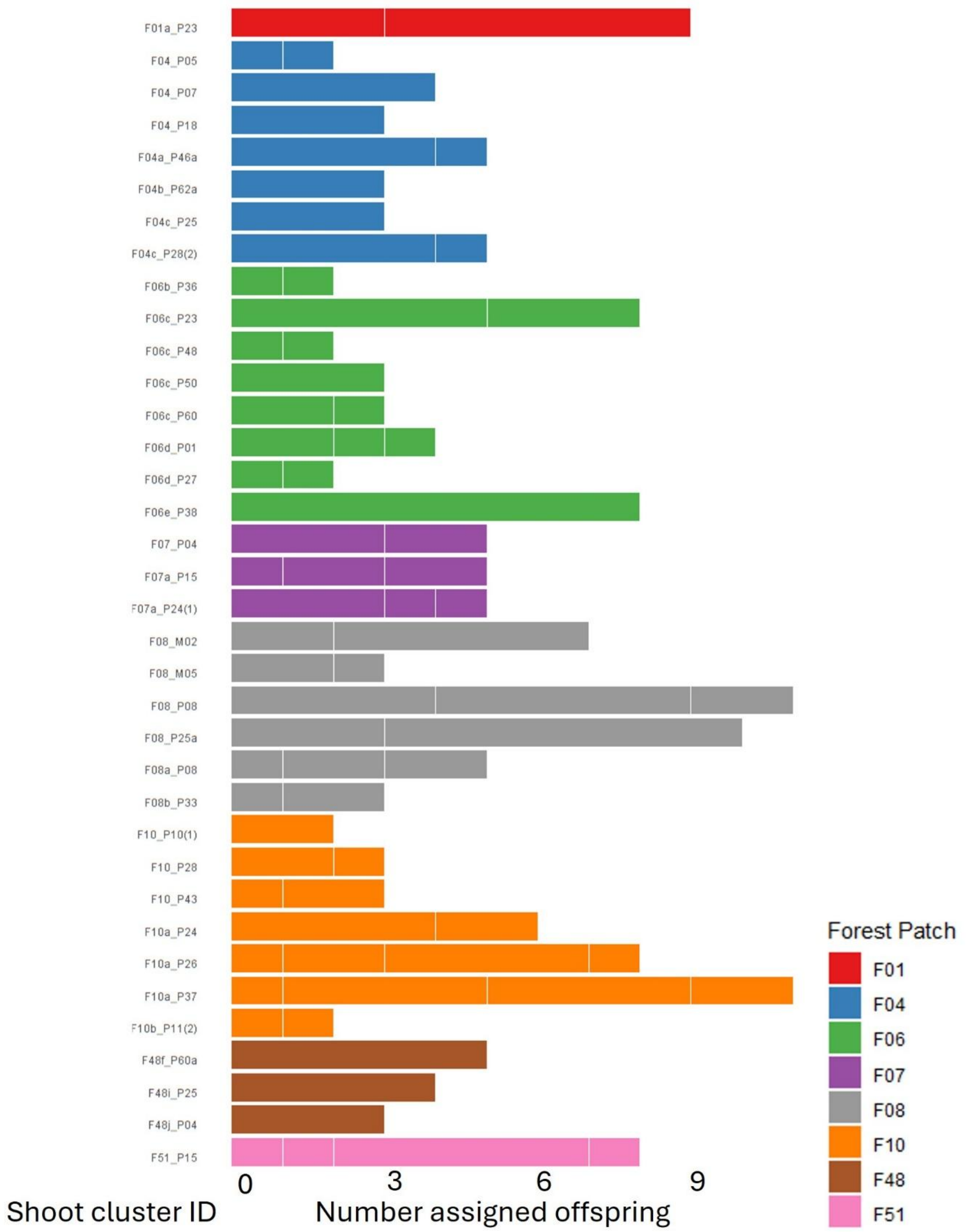

Figure S7.2: The figure shows the counts of offspring that were assigned to specific pollen donors from their own forest patch. The white vertical lines indicate from how many pollen receptors the offspring originated.

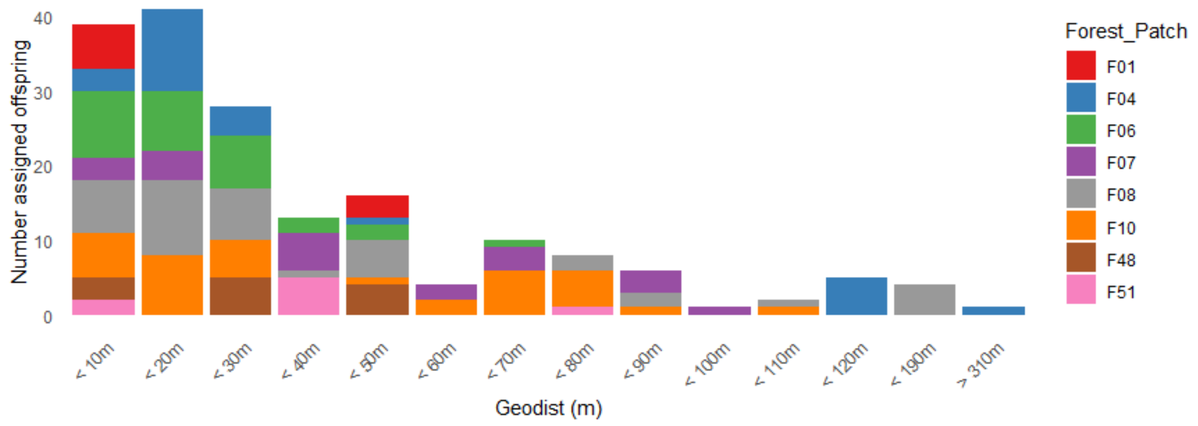

Figure S7.3: The figure shows the counts of offspring that were assigned to specific pollen donors from their own forest patch at specific geographic distances classes.

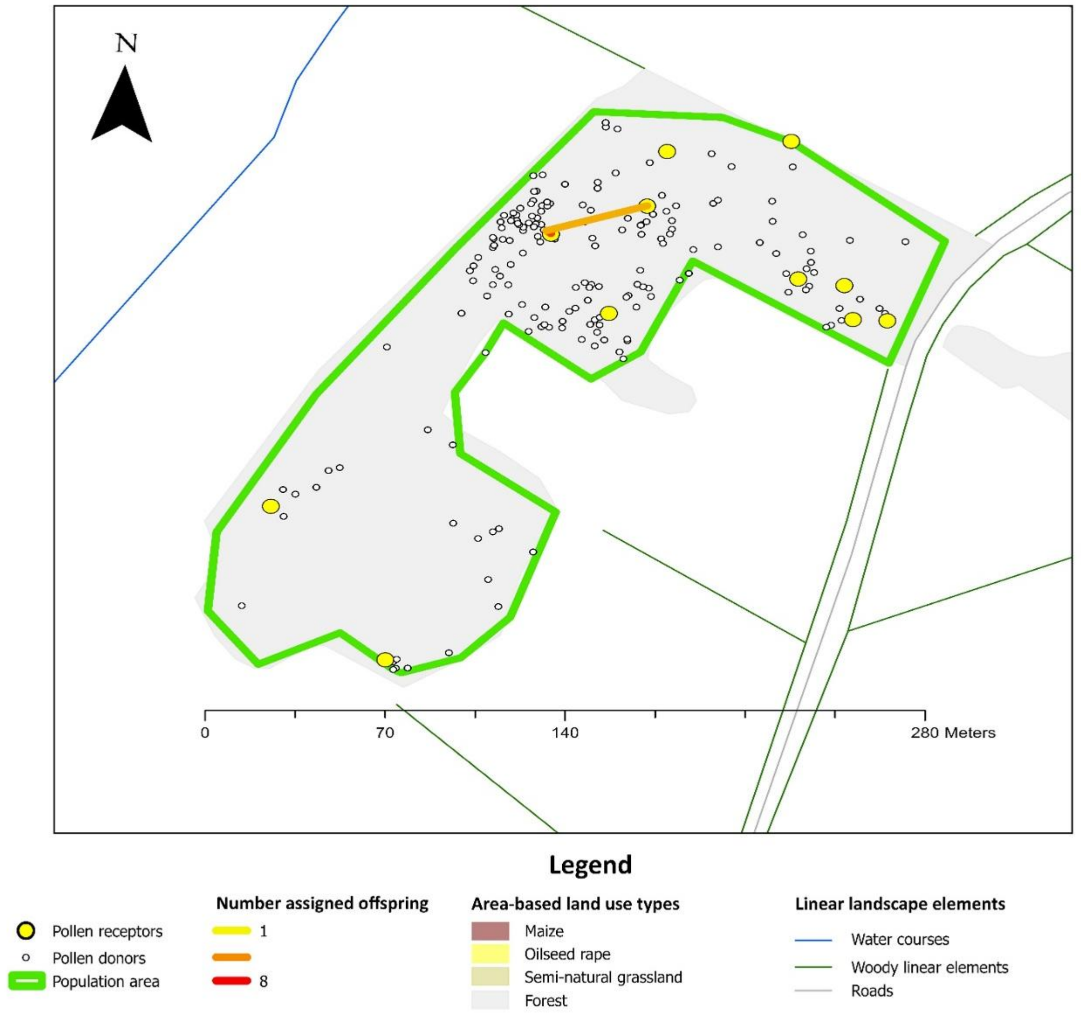

Figure S7.4a: Connection lines between pollen receptors (yellow dots) and identified pollen donors in forest patch F01. The color of the lines indicates the number of assigned offspring individuals. White areas represent land-use types not included in the study, mainly cereals and intensively managed grassland.

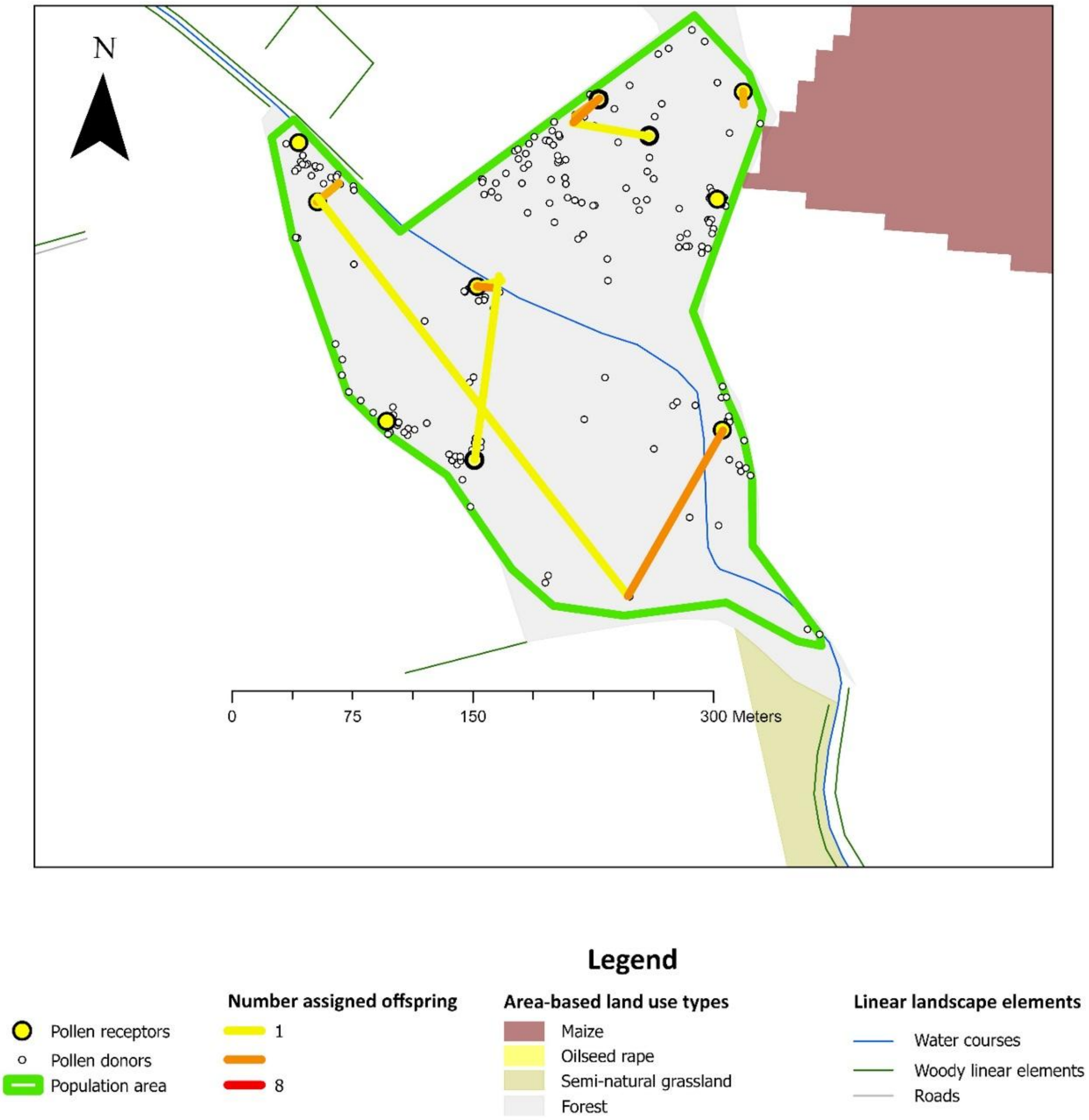

Figure S7.4b: Connection lines between pollen receptors (yellow dots) and identified pollen donors in forest patch F04. The color of the lines indicates the number of assigned offspring individuals. White areas represent land-use types not included in the study, mainly cereals and intensively managed grassland.

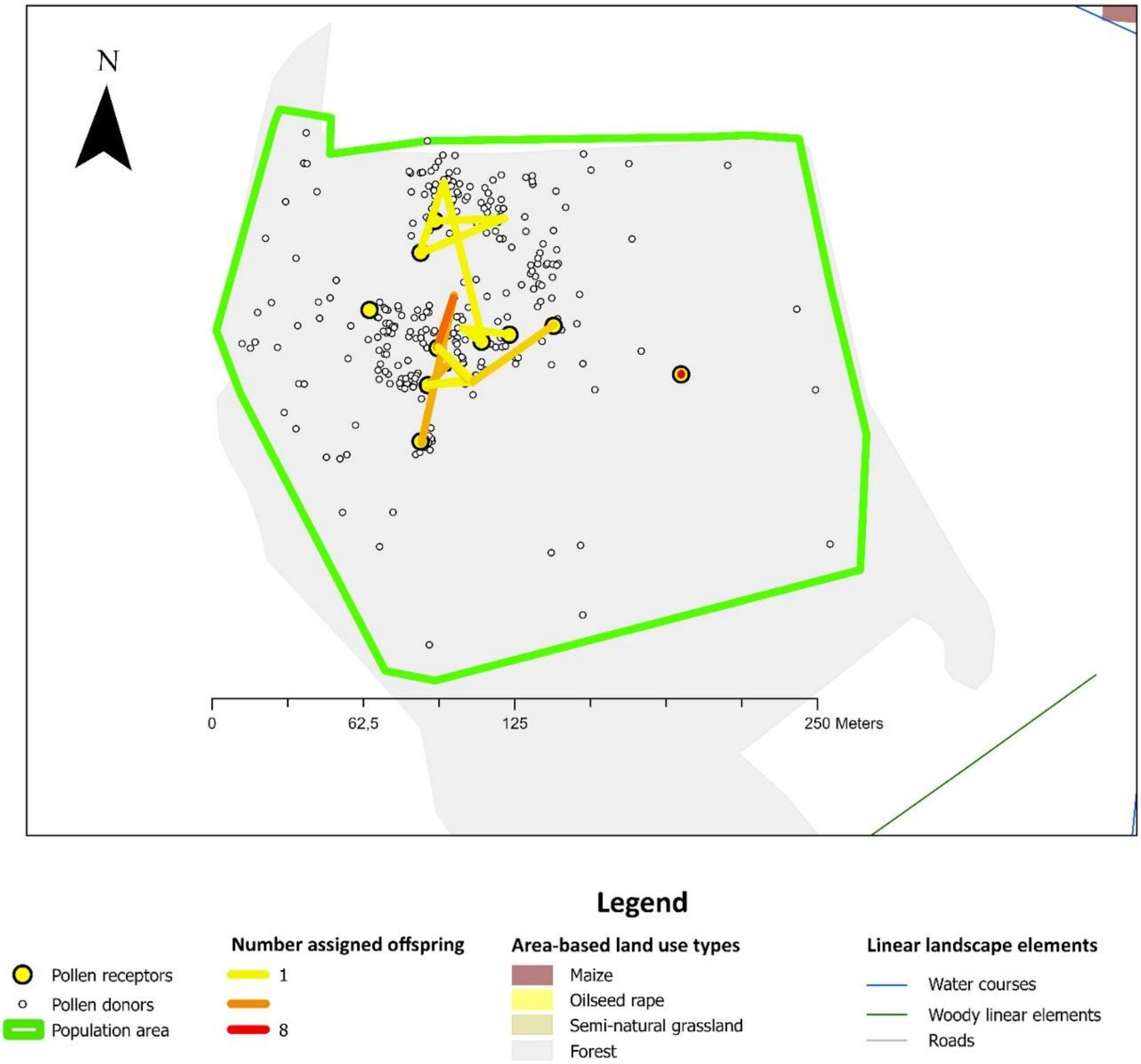

Figure S7.4c: Connection lines between pollen receptors (yellow dots) and identified pollen donors in forest patch F06. The color of the lines indicates the number of assigned offspring individuals. White areas represent land-use types not included in the study, mainly cereals and intensively managed grassland.

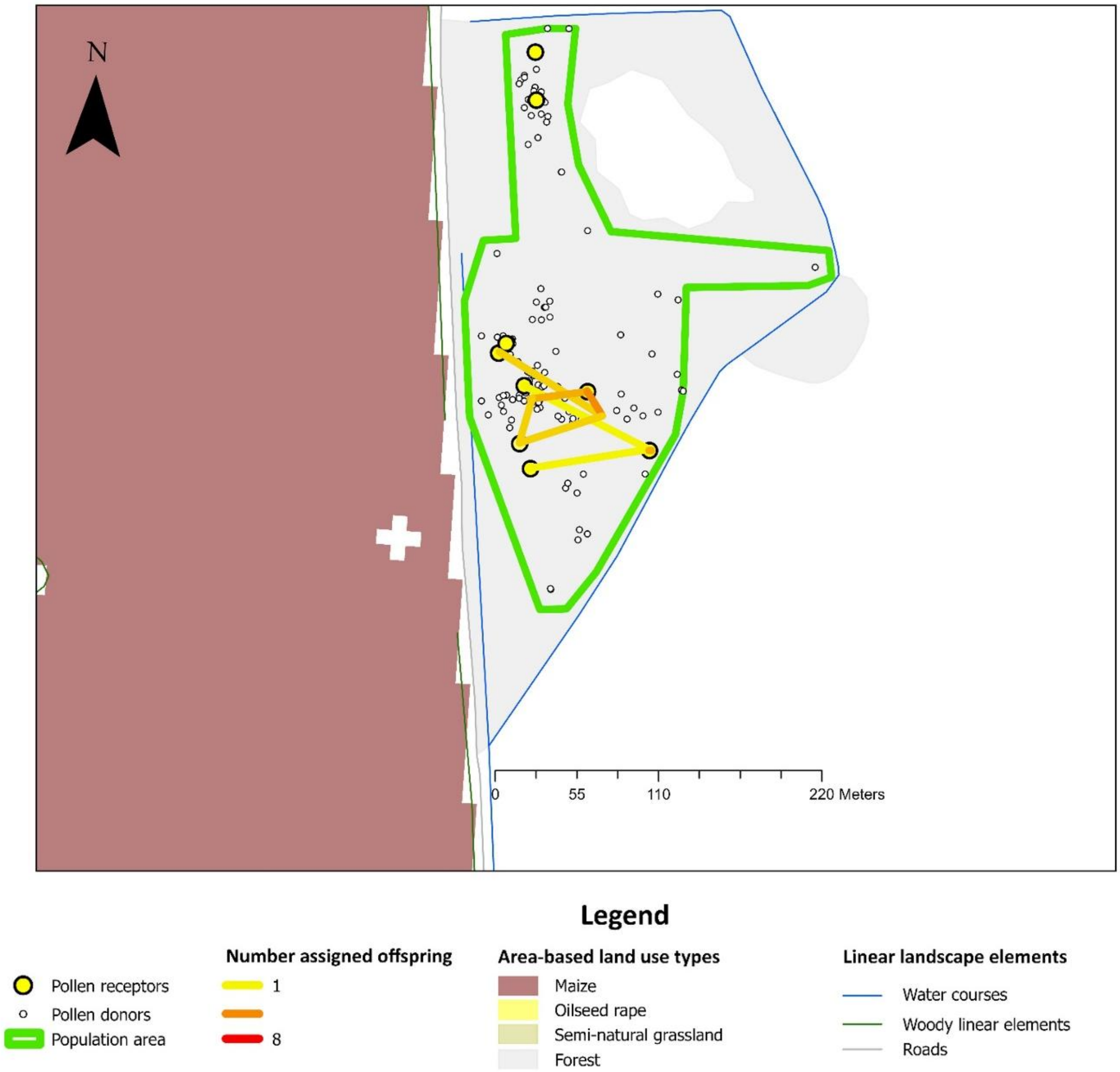

Figure S7.4d: Connection lines between pollen receptors (yellow dots) and identified pollen donors in forest patch F07. The color of the lines indicates the number of assigned offspring individuals. White areas represent land-use types not included in the study, mainly cereals and intensively managed grassland.

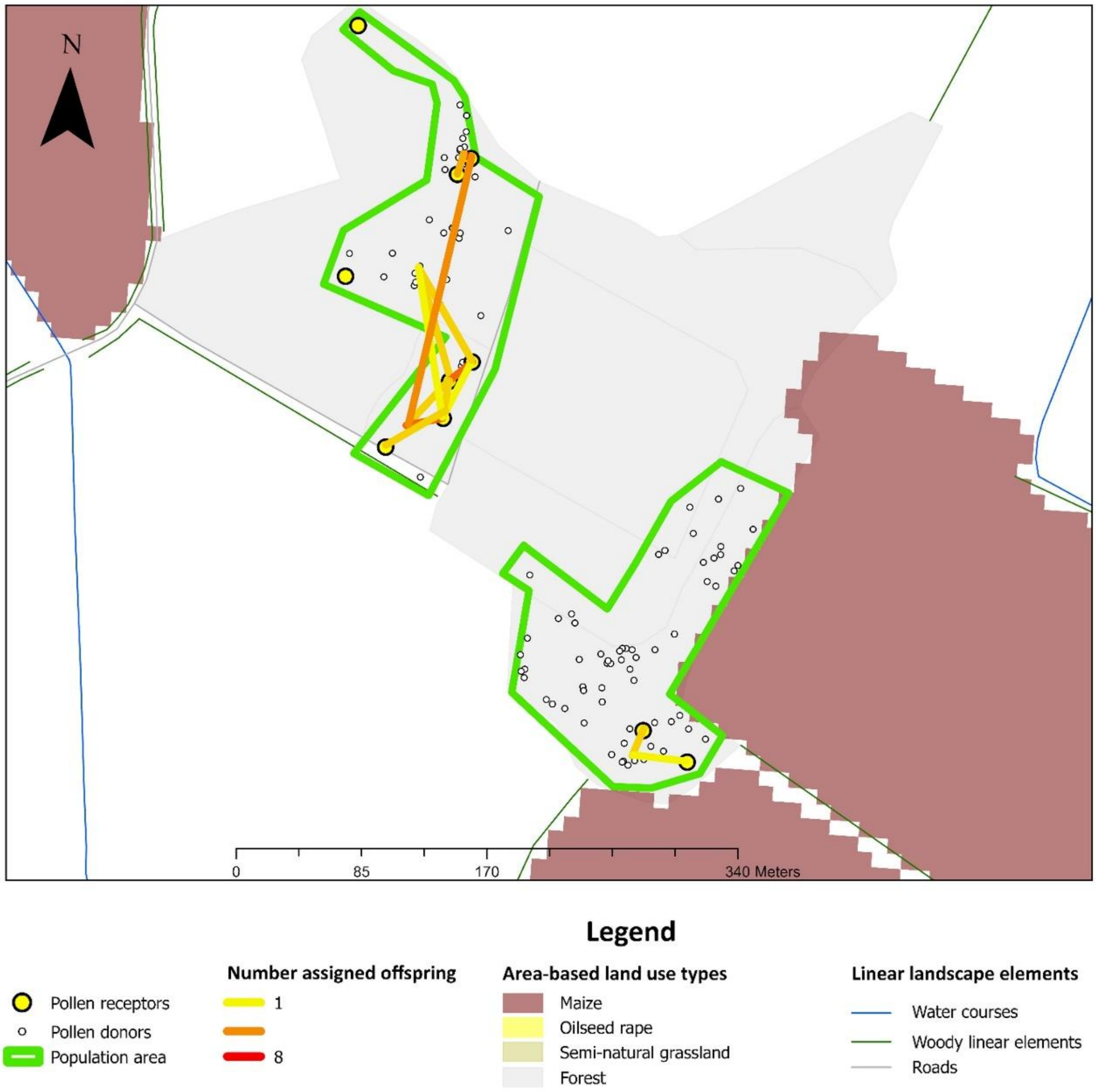

Figure S7.4e: Connection lines between pollen receptors (yellow dots) and identified pollen donors in forest patch F08. The color of the lines indicates the number of assigned offspring individuals. White areas represent land-use types not included in the study, mainly cereals and intensively managed grassland.

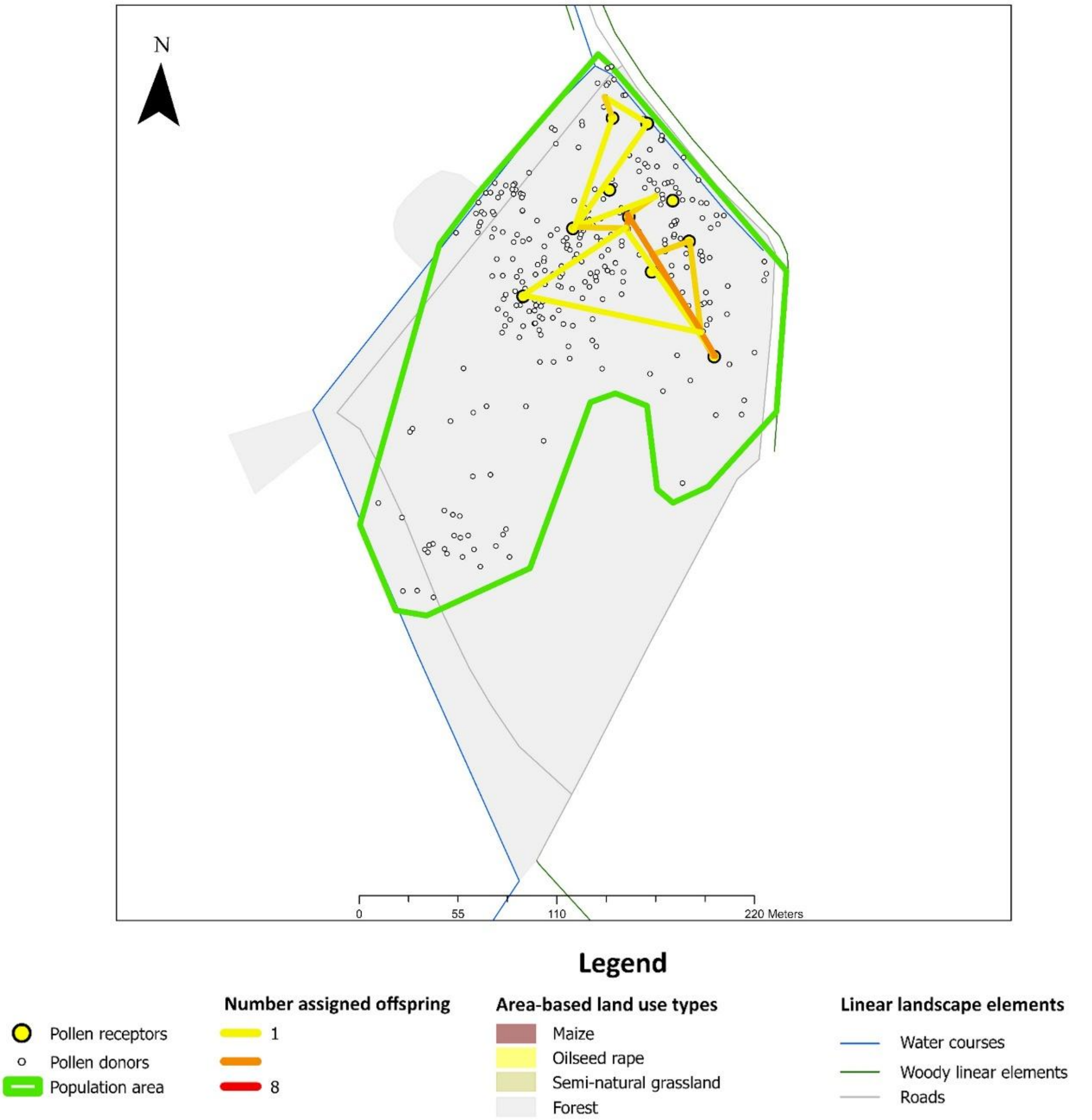

Figure S7.4f: Connection lines between pollen receptors (yellow dots) and identified pollen donors in forest patch F10. The color of the lines indicates the number of assigned offspring individuals. White areas represent land-use types not included in the study, mainly cereals and intensively managed grassland.

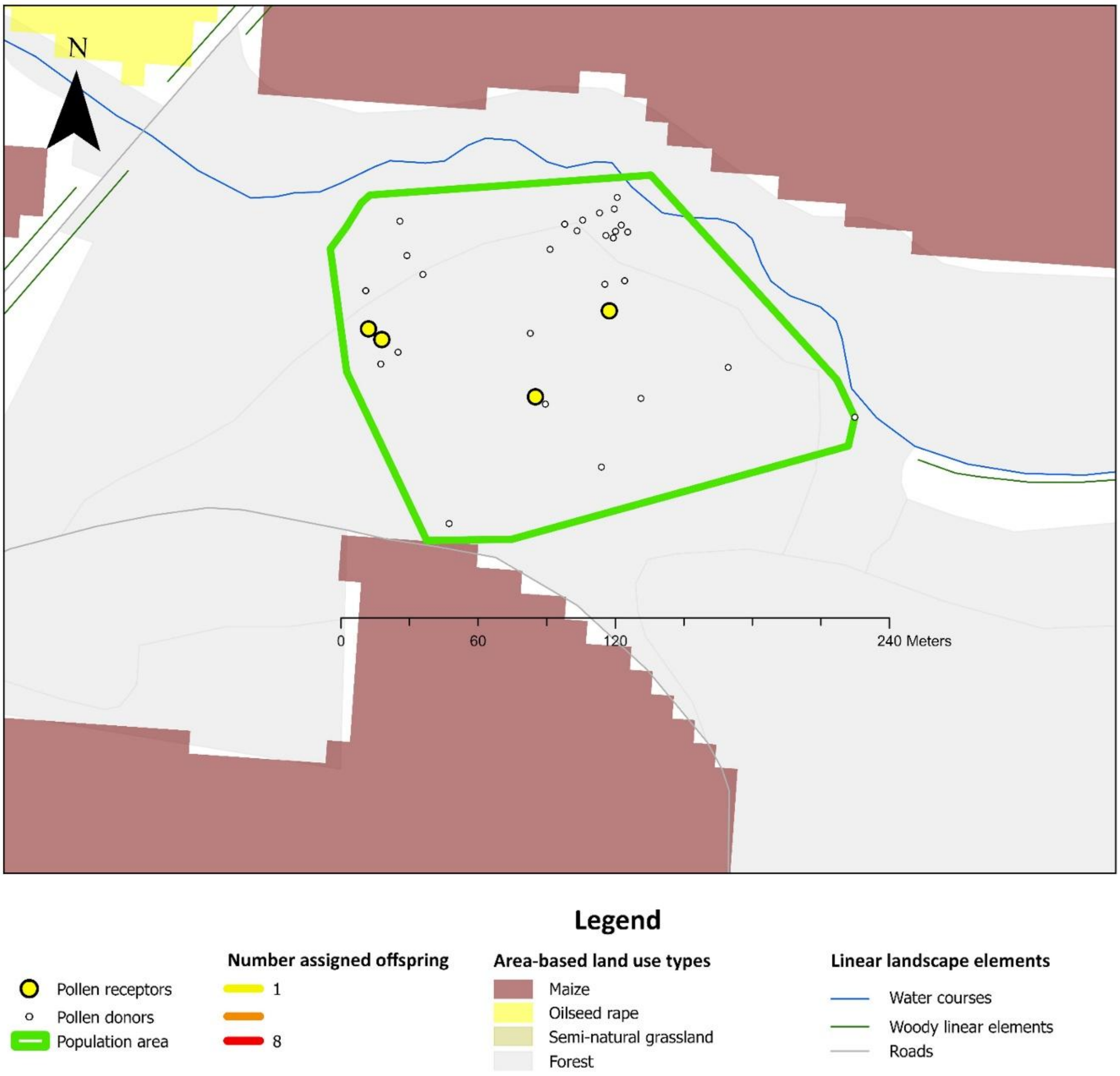

Figure S7.4g: Connection lines between pollen receptors (yellow dots) and identified pollen donors in forest patch F45. The color of the lines indicates the number of assigned offspring individuals. White areas represent land-use types not included in the study, mainly cereals and intensively managed grassland.

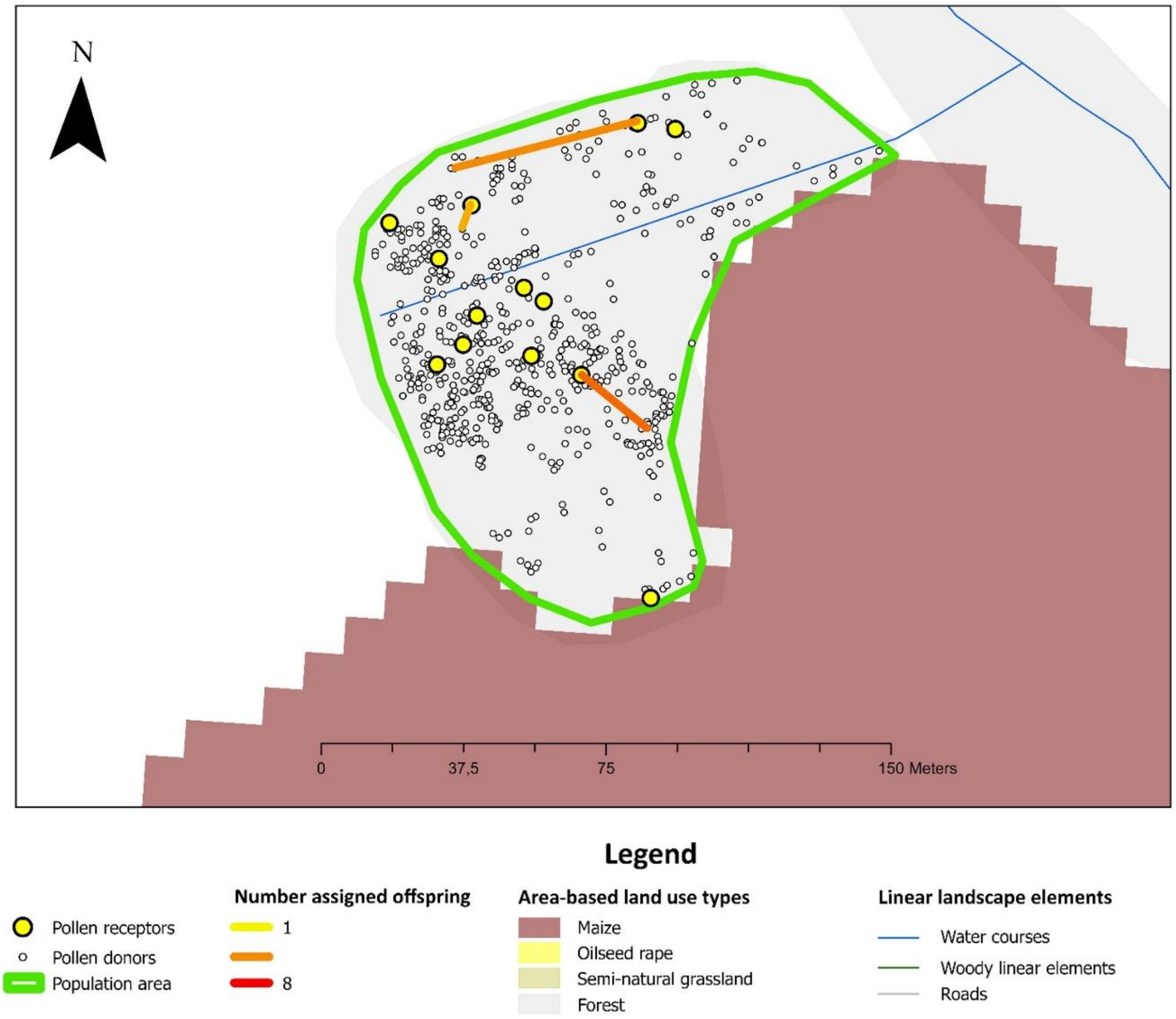

Figure S7.4h: Connection lines between pollen receptors (yellow dots) and identified pollen donors in forest patch F48. The color of the lines indicates the number of assigned offspring individuals. White areas represent land-use types not included in the study, mainly cereals and intensively managed grassland.

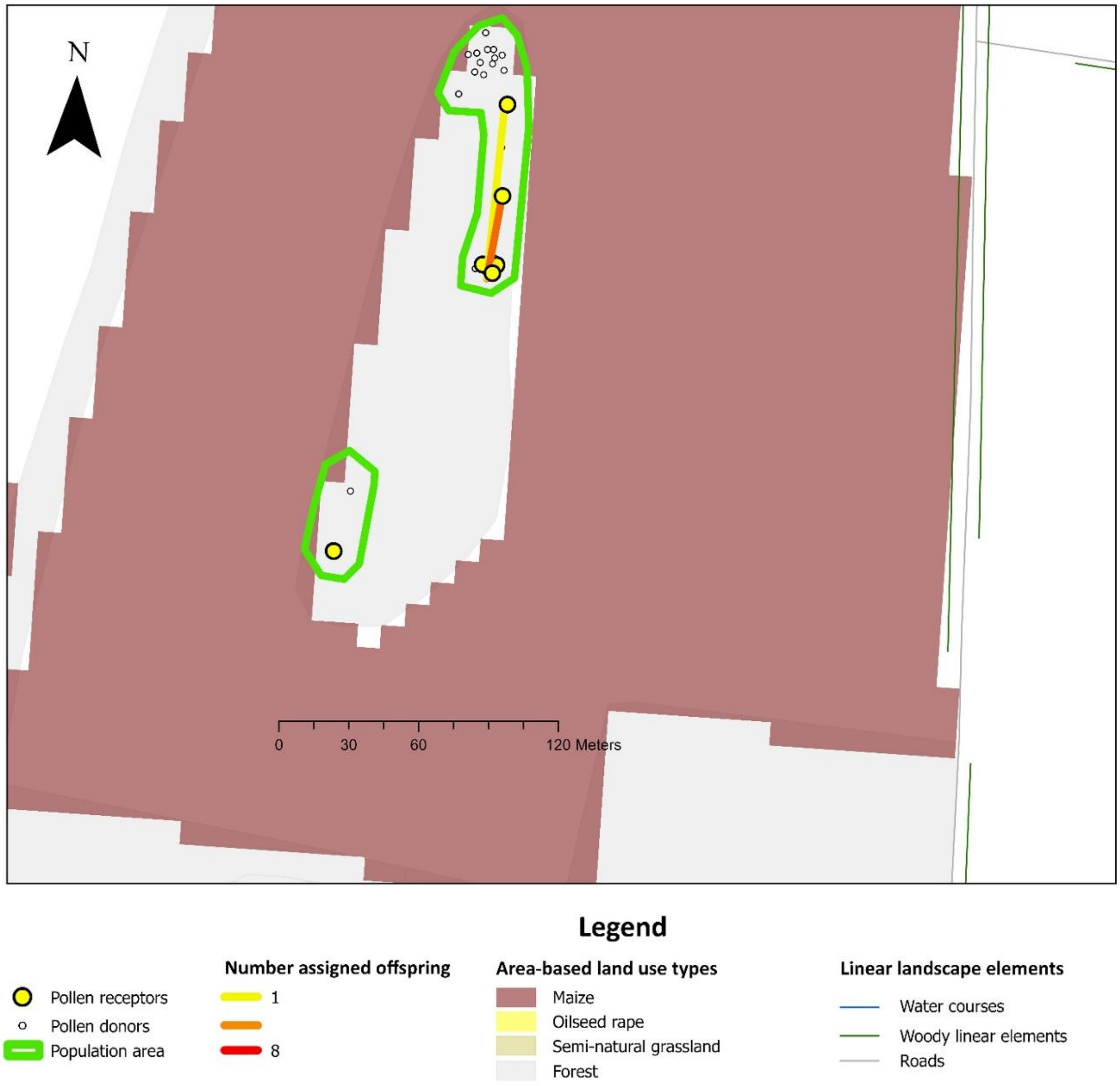

Figure S7.4i: Connection lines between pollen receptors (yellow dots) and identified pollen donors in forest patch F51. The color of the lines indicates the number of assigned offspring individuals. White areas represent land-use types not included in the study, mainly cereals and intensively managed grassland.

Supplement 8: Model outcome H2

Table S8.1: Model outcomes of the generalized linear mixed models (GLMM) for the conditional effects to testing H2. To test the effects on within-forest patch mating patterns (H2), we used GLMM with a conditional part (Poisson distribution) and a zero-inflation part (See section Data analysis for more details).

	Estimate	Standard error	*z*-statistic	*p*-value
(Intercept)	1.593	0.096	16.567	0.000
Shoot number	-0.048	0.059	-0.811	0.418
Distance_to_forest_edge	-0.057	0.083	-0.691	0.489
Interaction shoot number and distance_to_forest_edge	0.065	0.038	1.704	0.088

Table S8.2: model outcomes of for the generalized linear mixed models (GLMM) for the zero-inflation effects to testing H2. To test the effects on within-forest patch mating patterns (H2), we used GLMM with a conditional part (Poisson distribution) and a zero-inflation part (See section Data analysis for more details)

	Estimate	Standard error	*z*-statistic	*p*-value
(Intercept)	4.118	0.287	14.344	0.000
Shoot number	-0.262	0.0938	-2.796	0.005
Distance_to_forest_edge	-0.169	0.176	-0.957	0.338
Interaction shoot number and distance_to_forest_edge	-0.045	0.085	-0.532	0.594

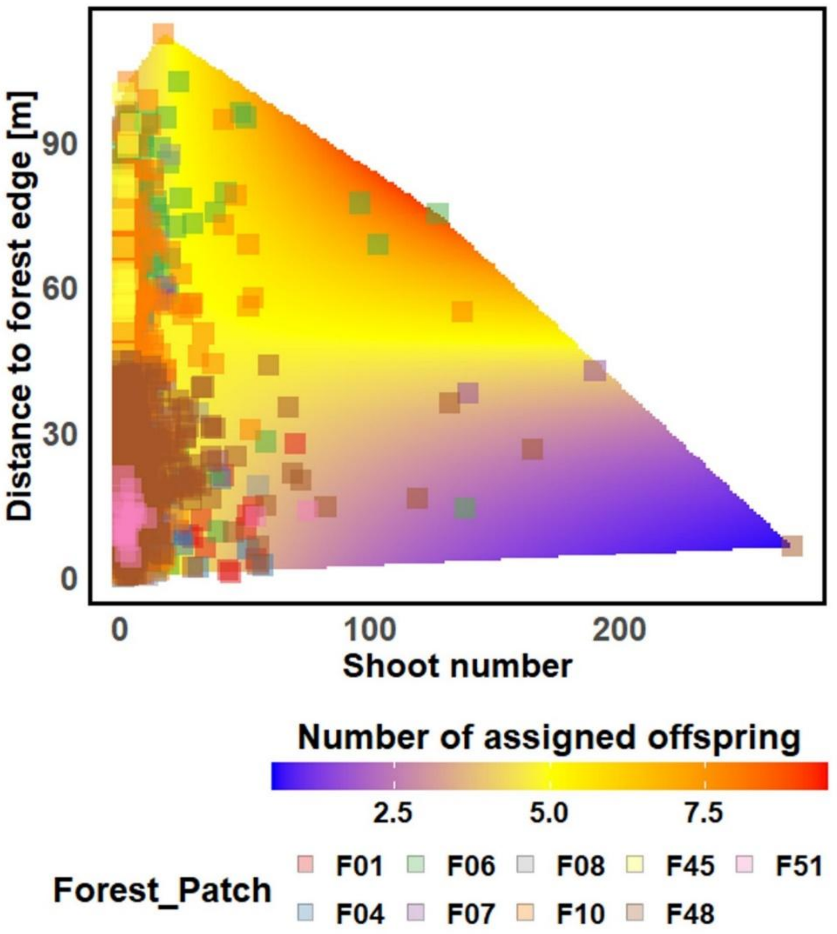

Figure S8.1: Interaction effect between shoot cluster size (shoot number) and distance to the forest edge on the number of assigned offspring (color gradient from blue to red). Colored squares represent the residuals, with colors indicating the forest patch from which the pollen receptors originated.

Supplement 9: Collinearity among included variables

Table S9.1: Test results for collinearity (Pearson correlation) among all landscape metrics with a p < 0.15 in single-metric models for PF_{within} in the 50 m buffer radius. Abbreviations are explained in Table 1.

L_ROAD_50	-0.20	
O:P_ ROAD_50	-0.07	0.23
	MAIZE_50	L_ROAD_50

Table S9.2: Test results for collinearity (Pearson correlation) among all landscape metrics with a p < 0.15 in single-metric models for A_r in the 50 m buffer radius. Abbreviations are explained in Table 1.

L_WATER_50	-0.14
	MAIZE_50

Table S9.3: Test results for collinearity (Pearson correlation) among all landscape metrics with a p < 0.15 in single-metric models for PF_{within} in the 250 m buffer radius. Abbreviations are explained in Table 1.

L_ROAD_250	-0.20	
N_POPULATIONS_250	-0.33	0.17
	MAIZE_250	L_ROAD_250

Figure S9.4: Test results for collinearity (Pearson correlation) among all landscape metrics with a p < 0.15 in single-metric models for A_r in the 250 m buffer radius. Abbreviations are explained in Table 1.

O:P_ WATER_250	-0.17
	L_WATER_250

Table S9.5: Test results for collinearity (Pearson correlation) among all landscape metrics with a $p < 0.15$ in single-metric models for PF_{within} cond part in the 1000 m buffer radius. Red colors indicate a correlation of |r| ≥ 0.7. Abbreviations are explained in Table 1.

	SEMNATGRASS_1000	N_POPULATIONS _1000	MAIZE_1000	L_WOOD_1000	L_ROAD_1000	L_WATER_1000	O:P_WATER_1000	O:P_WOOD_1000
N_POPULATIONS_1000	0.57							
MAIZE_1000	-0.34	-0.15						
L_WOOD_1000	-0.31	-0.38	-0.54					
L_ROAD_1000	-0.33	-0.38	0.60	-0.13				
L_WATER_1000	0.52	0.63	-0.56	0.23	-0.58			
O:P_WATER_1000	-0.26	0.21	0.43	0.01	0.17	0.07		
O:P_ WOOD_1000	0.25	0.36	0.39	-0.40	0.48	0.11	0.37	
RAPESEED_1000	0.24	0.04	0.32	-0.74	0.32	-0.40	-0.13	0.28

Table S9.6: Test results for collinearity (Pearson correlation) among all landscape metrics with a $p < 0.15$ in single-metric models for PF_{within} zero-inflation part in the 1000 m buffer radius. Red colors indicate a Pearson correlation of |r| ≥ 0.7. Abbreviations are explained in Table 1.

	L_WATER_1000	O:P_ WATER_1000	L_ROAD_1000	RAPESEED_1000	L_WOOD_1000	O:P_ WOOD_1000
O:P_WATER_1000	0.07					
L_ROAD_1000	-0.58	0.17				
RAPESEED_1000	-0.40	-0.13	0.32			
L_WOOD_1000	0.23	0.01	-0.13	-0.74		
O:P_WOOD_1000	0.11	0.37	0.48	0.28	-0.40	

Table S9.7: Test results for collinearity (Pearson correlation) among all landscape metrics with a $p < 0.15$ in single-metric models for A_r in the 1000 m buffer radius. Abbreviations are explained in Table 1.

N_ POPULATIONS _1000	-0.15			
SEMNATGRASS_1000	-0.34	0.57		
L_ROAD_1000	0.60	-0.38	-0.33	
	MAIZE_1000	N_ POPULATIONS _1000	SEMNATGRASS_1000	L_ROAD_1000

Supplement 10: Outcome of Model averaging for H3 and H4

Table S10.1: Overview of model averaging. Abbreviations are explained in Table 1. Effects on A_r were modeled with LMM and on PF_{within} with GLMM with a conditional (cond) and zero-inflation part (zi). Shown are the total number of tested models and the number of models with $\Delta AICc$ < 2.

	Buffer radius	Variables from single metric models	Total number of models	Number of models with $\Delta AICc$ < 2
PF_{within}	50	Cond:<br>• MAIZE<br>• L_ROAD<br>• O:P_ROAD<br>ZI:<br>---	9	1
A_r	50	• MAIZE<br>• L_WATER	4	2
PF_{within}	250	Cond:<br>• MAIZE<br>• L_ROAD<br>ZI:<br>• N_POPULATIONS	16	1
A_r	250	• L_WATER<br>• O:P_ WATER	3	1
PF_{within}	1000	Cond:<br>• MAIZE<br>• SEMNATGRASS<br>• RAPESEED<br>• L_ROAD<br>• L_WATER<br>Zi:<br>• RAPSEED<br>• L_ROAD<br>• L_WATER<br>• L_WOOD<br>• O:P_WOOD<br>• O:P_WATER	1320	14
A_r	1000	• MAIZE<br>• SEMNATGRASS<br>• L_ROAD<br>• N_POPULATIONS	11	3

Table S10.2: The table summarizes the outcome of the single best model for PF_{within} at the 50 m buffer. To test the effects of the landscape metrics on PF_{within}, generalized linear mixed models (GLMM) with a conditional (cond) part and a zero-inflation (zi) part were used (see Data Analysis section for more details).

	Estimate	Standard error	*z*-statistic	*p*-value
MAIZE_50 (cond)	0.384	0.442	0.869	0.385
MAIZE_50^2 (cond)	-0.910	0.355	-2.562	0.010

Table S10.3: The table summarizes the outcome of the model averaging procedure for A_r at the 50 m buffer. To test the effects of the landscape metrics on A_r, linear mixed models (LMM) were used (see Data Analysis section for more details).

	Estimate	Standard error	*z*-statistic	*p*-value
L_WATER_50	0.129	0.039	3.253	0.001
MAIZE_50	-0.077	0.045	1.689	0.091

Table S10.4: The table summarizes the outcome of the model averaging procedure for PF_{within} at the 250 m buffer. To test the effects of the landscape metrics on PF_{within}, generalized linear mixed models (GLMM) with a conditional (cond) part and a zero-inflation (zi) part were used (see Data Analysis section for more details).

	Estimate	Standard error	*z*-statistic	*p*-value
L_ROAD_250 (cond)	0.217	0.101	2.147	0.032
MAIZE_250 (cond)	-0.513	0.132	-3.891	0.0001
MAIZE_250^2 (cond)	-0.618	0.159	-3.89	0.0001
N. Populations 250 (zi)	1.189	0.425	2.794	0.005

Table S10.5: The table summarizes the outcome of the model averaging procedure for A_r at the 250 m buffer. To test the effects of the landscape metrics on A_r, linear mixed models (LMM) were used (see Data Analysis section for more details).

column name	Estimate	Standard error	*t*-statistic	*p*-value
L_WATER_250	-0.058	0.045	-1.268	0.209
O:P_WATER_250	-0.135	0.038	-3.596	0.0006
L_WATER_250 X O:P_WATER_250	-0.152	0.048	-3.141	0.003

Table S10.6: The table summarizes the outcome of the model averaging procedure for PF_{within} at the 1000 m buffer. To test the effects of the landscape metrics on PF_{within}, generalized linear mixed models (GLMM) with a conditional (cond) part and a zero-inflation (zi) part were used (see Data Analysis section for more details).

	Estimate	Standard error	*z*-statistic	*p*-value
MAIZE_1000 (cond)	-0.922	0.385	2.364	0.018
MAIZE_1000^2 (cond)	-0.701	0.309	2.227	0.026
RAPESEED_1000 (cond)	-0.444	0.244	1.795	0.073
RAPESEED_1000^2 (cond)	0.224	0.242	0.909	0.364
SEMNATGRASS_1000 (cond)	-0.128	0.239	0.524	0.600
SEMNATGRASS _1000^2 (cond)	-0.351	0.124	2.77	0.006
L_WATER_1000 (cond)	-0.383	0.378	0.995	0.32
RAPESEED_1000 (zi)	0.694	0.440	1.554	0.120
L_WATER_1000 (zi)	1.987	0.706	2.773	0.006
O:P_WATER_1000 (zi)	-1.916	1.066	1.769	0.077
L_WATER_1000 x O:P_WATER_1000 (zi)	-0.183	0.599	0.300	0.764
L_ROAD_1000 (zi)	0.761	0.492	1.52	0.129
L_WOOD_1000 (zi)	0.021	0.366	0.057	0.955
O:P_WOOD_1000 (zi)	-0.435	0.439	0.977	0.328
L_ WOOD _1000 x O:P_ WOOD _1000 (zi)	-1.446	0.430	3.308	0.001

Table S10.7: The table summarizes the outcome of the model averaging procedure for A_r at the 1000 m buffer. To test the effects of the landscape metrics on A_r, linear mixed models (LMM) were used (see Data Analysis section for more details).

	Estimate	Standard error	*z*-statistic	*p*-value
(Intercept)	2.961	0.045	64.964	0.000
L_ROADS_1000	-0.078	0.042	1.816	0.069
MAIZE_1000	-0.027	0.046	0.578	0.563
MAIZE_1000^2	-0.086	0.029	2.884	0.004
SEMNATGRASS_1000	0.032	0.036	0.851	0.395

Supplement 11: Models used for figures in the main text

Table S11: To illustrate the effects of the landscape metrics, we plotted their effects from the models in which they showed the strongest effects. The table below lists the models on which the specific figures are based, with standardized regression coefficients shown in brackets. To test the effects of the landscape metrics on PF_{within}, generalized linear mixed models (GLMM) with a conditional (cond) part and a zero-inflation (zi) part were used. To test the effects of the landscape metrics on A_r, linear mixed models (LMM) were used.

Figure ID	Model
**Figure 5a**	PF_{within} ~<br>cond: MAIZE_50 [0.38] + MAIZE_50^2 [-0.91]
**Figure 5b**	PF_{within} ~<br>cond: MAIZE_250 [-0.51] + MAIZE_250^2 [-0.62] + L_ROAD [0.22]<br>zi: N_ populations_250 [1.19]
**Figure 5c**	PF_{within} ~<br>cond: MAIZE_1000 [1.25] + MAIZE_1000^2 [-0.82] + L_WATER_1000 [-0.33] + RAPESEED_1000 [-0.62]<br>zi: L_WATER_1000 [2.09] + WATER_1000_O:P [-1.69] + RAPESEED_1000 [0.55] + L_WATER_1000 x WATER_1000_O:P [-0.02]
**Figure 5d**	A_r ~<br>MAIZE_1000[-0.06] + MAIZE_1000^2 [-0.1]
**Figure 5e**	PF_{within} ~<br>cond: MAIZE_1000 [-0.69] + MAIZE_1000^2 [-0.61] + SEMNATGRASS_1000[-0.15] + SEMNATGRASS^2_1000 [-0.36]<br>zi : L_WOOD_1000 [0.02] + WOOD_1000_O:P [-0.24] + L_WOOD_1000 x WOOD_1000_O:P [-1.32]
**Figure 6a**	A_r ~<br>L_WATER_250 [-0.06] + L_WATER_O:P [-0.14] + L_WATER_250: L_WATER_O:P [-0.15]
**Figure 6b**	PF_{within} ~<br>cond: MAIZE_1000 [-0.65] + MAIZE^2_1000 [-0.61] + SEMNATGRASS _1000 [-0.14] + SEMNATGRASS_1000^2 [-0.36]<br>zi L_ROAD_1000 [0.62] + L_WOOD_1000 [0]+ WOOD_1000_O:P [-0.69] + L_WOOD_1000: WOOD_1000_O:P [-1.61]
**Figure 7a**	PF_{within} ~<br>cond: L_ROAD [0.22]_250 + MAIZE_250 [-0.51]+ MAIZE_250^2 [-0.62] +<br>zi: N_populations_250 [1.19]
